# Transcutaneous spinal cord stimulation for functional recovery in individuals with hereditary spastic paraplegia: Protocol for a pilot clinical trial

**DOI:** 10.1371/journal.pone.0359948

**Published:** 2026-10-15

**Authors:** Vivek K. Pandey, Holly M. Elliott, Amara G. Sharp, Emma S. Ballenger, Zamiar Karianne, Parag Gad, Rich Cohn, Aaron G. Taylor, Emanuela Piermarini, Peter W. Baas, Samir P. Patel, Sara S. Salles, Zakari R. Dymock, Michael A. Samaan, Rahul Sachdeva

**Affiliations:** 1 Department of Physical Medicine and Rehabilitation, University of Kentucky, Lexington, United States of America; 2 Department of Kinesiology and Health Promotion, University of Kentucky, Lexington, United States of America; 3 Spinal Cord and Brain Injury Research Center (SCoBIRC), University of Kentucky, Lexington, United States of America; 4 Department of Physiology, University of Kentucky, Lexington, United States of America; 5 SpineX Inc, Los Angeles, California, United States of America; 6 Independent Consultant, Chapel Hill, North Carolina, United States of America; 7 Person with Lived Experience, Lexington, United States of America; 8 Department of Neurobiology and Anatomy, College of Medicine, Drexel University, Philadelphia, United States of America; PLOS: Public Library of Science, UNITED KINGDOM OF GREAT BRITAIN AND NORTHERN IRELAND

## Abstract

**Background:**

Hereditary spastic paraplegia (HSP) is a rare genetic neurodegenerative disorder characterized by progressive lower-extremity spasticity, weakness, and gait impairment resulting from corticospinal tract degeneration. With no disease-modifying therapies available, novel strategies to improve motor function are needed. Transcutaneous spinal cord stimulation (tSCS) is a non-invasive neuromodulation approach that may improve motor function but has not been prospectively evaluated in HSP. This study will assess the safety, feasibility, and preliminary efficacy of repeated tSCS in adults with HSP.

**Methods and analysis:**

This prospective, open-label, single-arm pilot trial will enroll 15 adults with HSP. Participants will receive 16 tSCS sessions over 8 weeks targeting the lower thoracic and upper lumbar vertebral levels, followed by an 8-week follow-up. Outcomes will assess gait performance, spasticity, disease severity, mobility, walking endurance, muscle strength, 3D gait biomechanics, electromyography, and HSP-specific patient-reported measures. Exploratory blood-based neurodegenerative biomarkers and metabolomic and lipidomic profiles will investigate potential biological correlates of treatment response. Assessments will occur at baseline and weeks 4, 8, and 16. Feasibility and safety will be evaluated through recruitment, retention, adherence, and adverse events.

## Introduction

Hereditary spastic paraplegia (HSP) comprises a heterogeneous group of inherited neurodegenerative disorders characterized by progressive lower-extremity spasticity, weakness, hyperreflexia, and gait dysfunction. More than 90 causative genes and numerous disease subtypes have been identified, reflecting substantial genetic and phenotypic heterogeneity [1–3]. Despite this diversity, HSP is characterized by a length-dependent axonopathy affecting the corticospinal tracts and dorsal column pathways, resulting in progressive motor and mobility impairment [4]. Clinical manifestations range from pure forms dominated by lower-extremity spasticity and weakness to complex phenotypes involving cerebellar dysfunction, peripheral neuropathy, seizures, cognitive impairment, optic neuropathy, and other neurological features [3,4]. Bladder and bowel dysfunction, fatigue, pain, and reduced quality of life are also common [5,6], while cognitive impairment and dementia occur in selected subtypes, including SPG4 and SPG11 [7,8].

Although rare, HSP imposes substantial individual and socioeconomic burden. Progressive gait impairment can lead to falls, loss of independence, reduced social and occupational participation, and increasing reliance on assistive devices and caregiver support [9]. Annual healthcare and societal costs have been estimated to exceed €27,000 per affected individual [10].

Despite advances in understanding HSP pathophysiology, treatment remains largely symptomatic. Current interventions include anti-spasticity medications, botulinum toxin injections, physical therapy, orthotic management, and assistive technologies [11]. These approaches may improve symptoms and function but do not prevent progressive mobility decline. For severe or refractory symptoms, intrathecal baclofen, SDR, or orthopedic surgery may be considered; however, their invasiveness and associated surgical or device-related risks limit broader applicability [12]. Thus, therapeutic strategies capable of providing durable improvements in motor function and mobility are needed.

Neuromodulation of spinal sensorimotor networks has emerged as a promising strategy for impaired motor control. In HSP, two case reports demonstrated improvements in gait, lower-extremity strength, and spasticity with epidural spinal cord stimulation (eSCS), suggesting that spinal circuits remain responsive to neuromodulation despite progressive upper motor neuron degeneration [13,14]. However, eSCS requires surgical implantation. Transcutaneous spinal cord stimulation (tSCS) provides a noninvasive alternative that recruits many of the same spinal neural pathways [15] and has demonstrated improvements in motor and autonomic function in individuals with spinal cord injury [16–19] and other neurological populations, including cerebral palsy [19–22]. tSCS is thought to activate large-diameter dorsal root afferents, modulating spinal interneuronal circuits, sensorimotor integration, residual descending drive, and motor neuron excitability [23,24]. These mechanisms are particularly relevant to HSP, where impaired descending control and abnormal spinal excitability contribute to motor dysfunction and spasticity.

Despite this rationale, no prospective clinical study has systematically evaluated tSCS in HSP. Furthermore, treatment responses have not been characterized using an integrated framework combining clinical outcomes with gait biomechanics, muscle activation, autonomic function, and blood-based molecular measures. Such multimodal assessment may help characterize functional responses to tSCS and identify physiological and biological correlates of treatment response.

The present pilot study will evaluate the safety, feasibility, and preliminary efficacy of an 8-week tSCS intervention in adults with HSP. Clinical and functional assessments will be complemented by quantitative gait biomechanics, muscle strength, disease-specific patient-reported and autonomic outcomes, and exploratory metabolomic, lipidomic, and neurodegenerative biomarker analyses. This integrated approach will provide preliminary clinical and mechanistic evidence to inform future controlled trials and precision neuromodulation strategies for HSP.

## Methods and analysis

### Study design

This study is a prospective, single-arm, open-label pilot-clinical trial designed to evaluate the feasibility, safety and preliminary efficacy of tSCS in individuals with HSP, while establishing key feasibility metrics and generating preliminary estimates of treatment effects to inform the design of future controlled clinical trials. The intervention comprises 16 non-invasive spinal cord stimulation sessions delivered twice weekly over 8 weeks. Participants will then enter an 8-week follow-up phase to assess the persistence of treatment-related effects. This study will be conducted in three phases. Phase 1 will consist of baseline and acute assessments, including eligibility screening, informed consent, clinical characterization, and collection of baseline functional, biomechanical, patient-reported, metabolomics and biomarker measures. Phase 2 will comprise the intervention period, during which participants will receive 16 sessions of tSCS for 8 weeks (2 times per week). Phase 3 will consist of an 8-week post-intervention follow-up period designed to evaluate the persistence of neuromodulatory effects following cessation of stimulation. Outcome assessments will be performed at week 1 (baseline and acute), week 4 (mid-intervention), week 8 (post-intervention) and week 16 (follow-up). Assessments will encompass multiple domains of function, including functional gait performance, lower-extremity spasticity, quadriceps muscle strength, patient-reported outcomes, quantitative gait biomechanics, LUT and bowel function and exploratory blood-based metabolomic and biomarker analyses. This multidimensional assessment framework is intended to characterize both the functional and molecular effects of tSCS in individuals with HSP.

The study protocol was developed in accordance with the Standard Protocol Items: Recommendations for Interventional Trials (SPIRIT) guidelines and is summarized in Fig 1 [25]. The protocol received approval from the University of Kentucky Institutional Review Board (IRB #109294) on January 21, 2026, and the trial was subsequently registered on ClinicalTrials.gov (NCT07417943).

**Fig 1:**
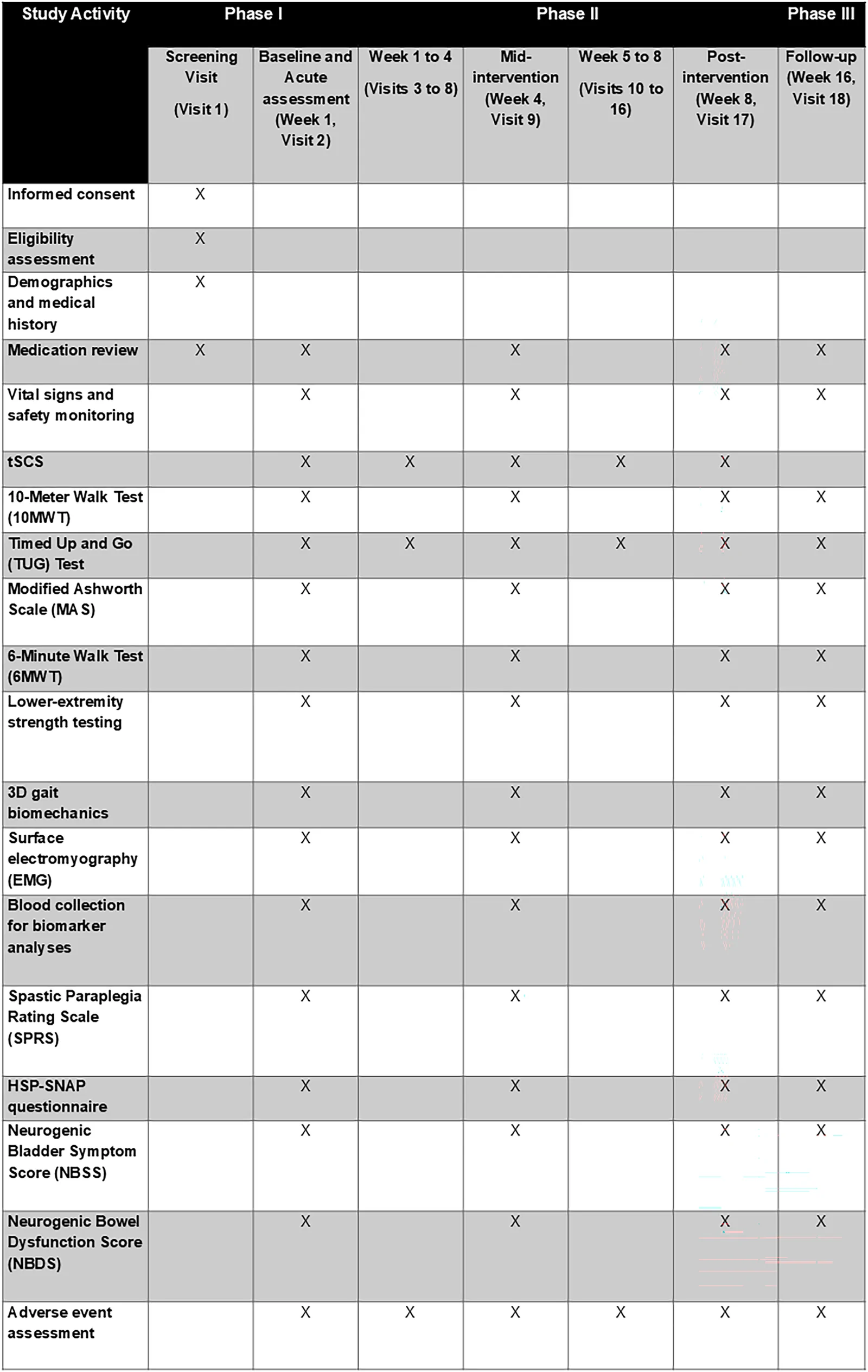
SPIRIT-compliant schedule of participant enrolment, intervention, and outcome assessments.

**Fig 2:**
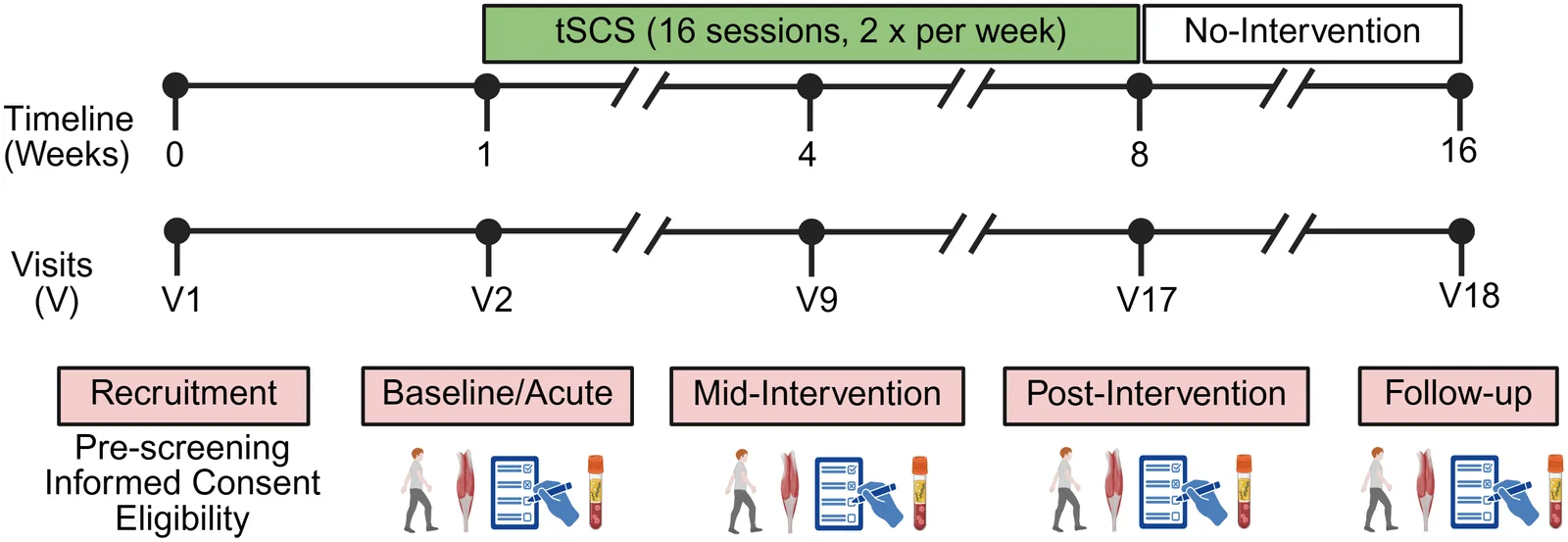
Study timeline and assessment schedule. Following recruitment, pre-screening, informed consent, and eligibility confirmation, participants will undergo baseline assessments during Week 1. After the first tSCS session, an acute assessment will be performed to evaluate the immediate effects of stimulation. Participants will then complete the remaining 15 tSCS sessions, for a total of 16 sessions delivered twice weekly over 8 weeks. Outcome measures will be obtained at baseline and acute (Week 1), mid-intervention (Week 4), post-intervention (Week 8), and follow-up (Week 16), with no intervention administered during the follow-up period. Primary outcome measures include the 10-Meter Walk Test (10MWT), Timed Up and Go (TUG), Modified Ashworth Scale (MAS), and Spastic Paraplegia Rating Scale (SPRS). Secondary outcome measures include the 6-Minute Walk Test (6MWT), 3D gait analysis, electromyography (EMG), isometric quadriceps muscle strength testing, and HSP-SNAP. Ancillary outcome measures include lower urinary tract and bowel function assessments, plasma biomarker profiling, and metabolomic analyses.

### Study population

This pilot study aims to enroll 15 adults with genetically confirmed or clinically diagnosed HSP at the University of Kentucky in Lexington, Kentucky, USA. Potential participants will undergo an initial screening to determine eligibility. Individuals meeting preliminary eligibility criteria will be invited to complete a screening visit during which study procedures, potential risks, and participant responsibilities will be reviewed. Written informed consent will be obtained prior to the initiation of any study-related procedures. The target sample size of 15 participants was selected based on the exploratory nature of the study and the rarity of HSP. As a pilot investigation, the primary objective is to evaluate feasibility, safety, participant retention, and preliminary treatment effects associated with tSCS, which is crucial to inform the design and sample size calculations of future controlled clinical trials.

Recruitment is expected to be completed by August 2027, with final follow-up assessments and data collection completed by December 2027. Data analysis is expected to be completed by January 2028, with study results available thereafter.

### Inclusion criteria

Participants must meet the following criteria:

- Age ≥ 18 years.
- Genetically confirmed or clinically diagnosed HSP.
- Stable medications for spasticity and other neurological symptoms for at least 4 weeks prior to enrollment.
- Ability to participate in study visits and assessments with or without assistive devices.
- If ambulatory, ability to walk at least 10 meters with or without an assistive device.
- Capacity to provide informed consent, comply with study procedures, and communicate in English.

### Exclusion criteria

Participants meeting any of the following criteria will be excluded:

- Presence of implanted electronic devices, including cardiac pacemakers, deep brain stimulators, spinal cord stimulators, or intrathecal pumps.
- Severe cardiopulmonary disease that would make participation unsafe.
- Open skin lesions or severe dermatological conditions at intended electrode placement sites.
- Pregnancy or plans to become pregnant during the intervention period.
- Recent lower-extremity surgery that may affect mobility outcomes.
- Inability to participate in basic movement or mobility assessments, even with customary mobility aids. Wheelchair users and individuals using assistive devices will be eligible provided that assessments can be completed using their usual mobility strategies.
- Cognitive or psychiatric conditions that impair the ability to provide informed consent or comply with study procedures.
- Active or ongoing urinary tract infection at the time of enrollment.
- History of Epilepsy.
- Participation in another interventional clinical trial that could influence mobility or spasticity outcomes during the study period.
- Changes within the previous 4 weeks in medications or treatments affecting spasticity or movement, including baclofen, tizanidine, or botulinum toxin injections.
- Anticipated initiation or modification of treatments affecting mobility or spasticity during the study period.
- Any condition that, in the opinion of the investigators, would increase study risk or confound interpretation of study outcomes.

### Intervention

Participants will receive an 8-week tSCS intervention, consisting of 16 sessions delivered twice weekly (Fig 2). Each stimulation session will be 60 minutes, with a total visit duration of approximately 90 minutes, including preparation, stimulation delivery, and safety monitoring. tSCS will be delivered using a noninvasive spinal neuromodulation system (xStep^TM^, SpineX Inc., Northridge, California, USA). Two self-adhesive round electrodes positioned along the midline over the lower thoracic (T11-T12) and/or upper lumbar (L1-L2) vertebral levels will serve as cathodes, while two rectangular electrodes placed bilaterally over the iliac crests will serve as anodes (Fig 3).

**Fig 3:**
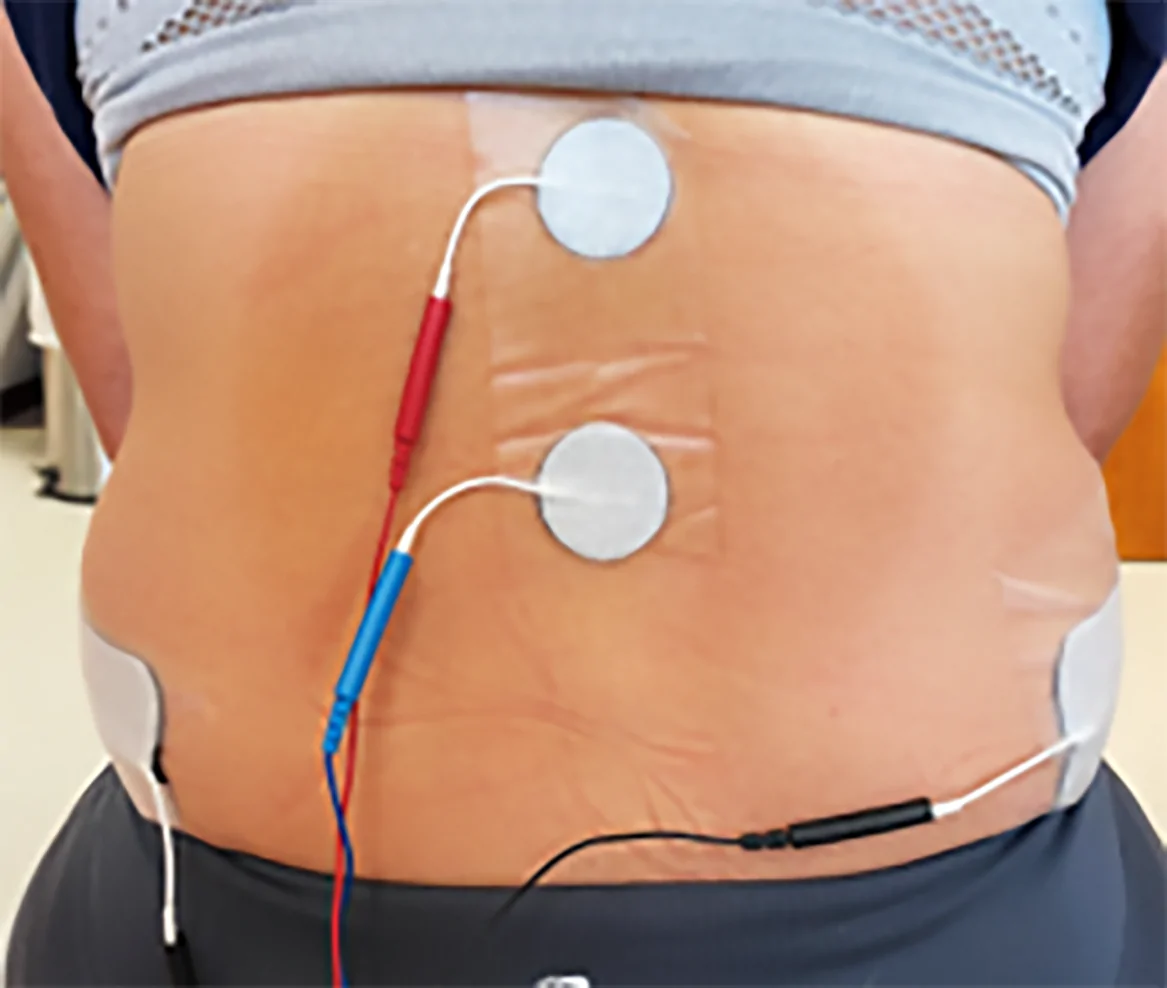
Placement of stimulation electrodes over the lower thoracic and upper lumbar spinal levels.

Stimulation will be delivered as a high-frequency pulsed current using frequencies within a predefined range of approximately 1–90 Hz in combination with a carrier frequency [10 kHz] to enhance participant comfort [26]. Current amplitude will be initiated at approximately 10 mA and will be progressively increased in small increments until a comfortable and tolerable stimulation intensity is achieved, remaining below the threshold required to elicit visible muscle contractions. Stimulation parameters will be individualized at each visit according to participant tolerance and clinical response.

Prior to each session, the skin at electrode placement sites will be inspected. If excessive hair is present, it will be clipped using an electric trimmer to ensure optimal electrode contact and minimize skin-electrode impedance. Participants will be continuously monitored throughout stimulation for discomfort, autonomic symptoms, skin irritation, and other adverse events. Depending on functional status, participants will receive tSCS while sitting, standing, or performing mobility-related tasks.

## Outcome measures

A multidimensional outcome assessment framework (Fig 2) will be used to evaluate the effects of tSCS on motor function, mobility, spasticity, patient-reported symptoms, autonomic function, circulating metabolites and biomarkers of disease severity as well as of treatment response (Table 1).

**Table 1:** Outcome measures at Baseline, Acute (Week 1), mid (Week 4), post intervention (Week 8) and during follow up (Week 16).

Domain	Outcome measure	Category
Walking speed	10-Meter Walk Test (10MWT)	Primary
Functional mobility	Timed Up and Go (TUG)	Primary
Spasticity	Modified Ashworth Scale (MAS)	Primary
Disease severity	Spastic Paraplegia Rating Scale (SPRS) Questionnaire	Primary
Walking endurance	6-Minute Walk Test (6MWT)	Secondary
Muscle strength	Isokinetic Dynamometry (Biodex)	Secondary
Neuromuscular activity	Surface electromyography (EMG)	Secondary
Gait biomechanics	Three Dimensional (3D) Gait Analysis	Secondary
Symptom burden	Hereditary Spastic Paraplegia-Self Notion and Perception (HSP-SNAP) Questionnaire	Secondary
Lower urinary tract function	Neurogenic Bladder Symptom Score (NBSS) Questionnaire	Ancillary
Bowel function	Neurogenic Bowel Dysfunction Score (NBDS) Questionnaire	Ancillary
Biomarker profile	Plasma Biomarkers (e.g., Neurodegeneration, injury and inflammation)	Exploratory
Metabolic profile	Plasma Metabolomic Analysis (Carbohydrate, Protein, and Lipid Metabolites)	Exploratory

adjustments will be documented. For participants unable to complete the 10MWT, the reason for non-completion will be recorded.

## Primary outcomes

The primary outcomes (Table 1) of this study will be changes in functional gait performance and lower-extremity spasticity following the 8-week tSCS intervention.

### 10-Meter Walk Test (10MWT)

Walking performance will be assessed using the 10-Meter Walk Test (10MWT), a reliable and widely used measure of gait performance in neurological populations [27–30]. Participants will complete 2–3 trials at a comfortable walking speed, and the average time across trials will be used for analysis [28,29]. To minimize the influence of acceleration and deceleration during comfortable walking, timing will be recorded over the central 6 m of the walkway (2 – 8m timing zone) (Fig 4A). Gait speed (m/s) will be calculated by dividing the timed distance by the average completion time. Participants will be permitted to use their customary assistive devices, orthoses, and footwear, which will be documented and maintained consistently across assessment visits whenever possible. In addition to gait speed, qualitative observations including loss of balance, near falls, uneven step length, scissoring gait, excessive spasticity, fatigue, pain, and device

**Fig 4:**
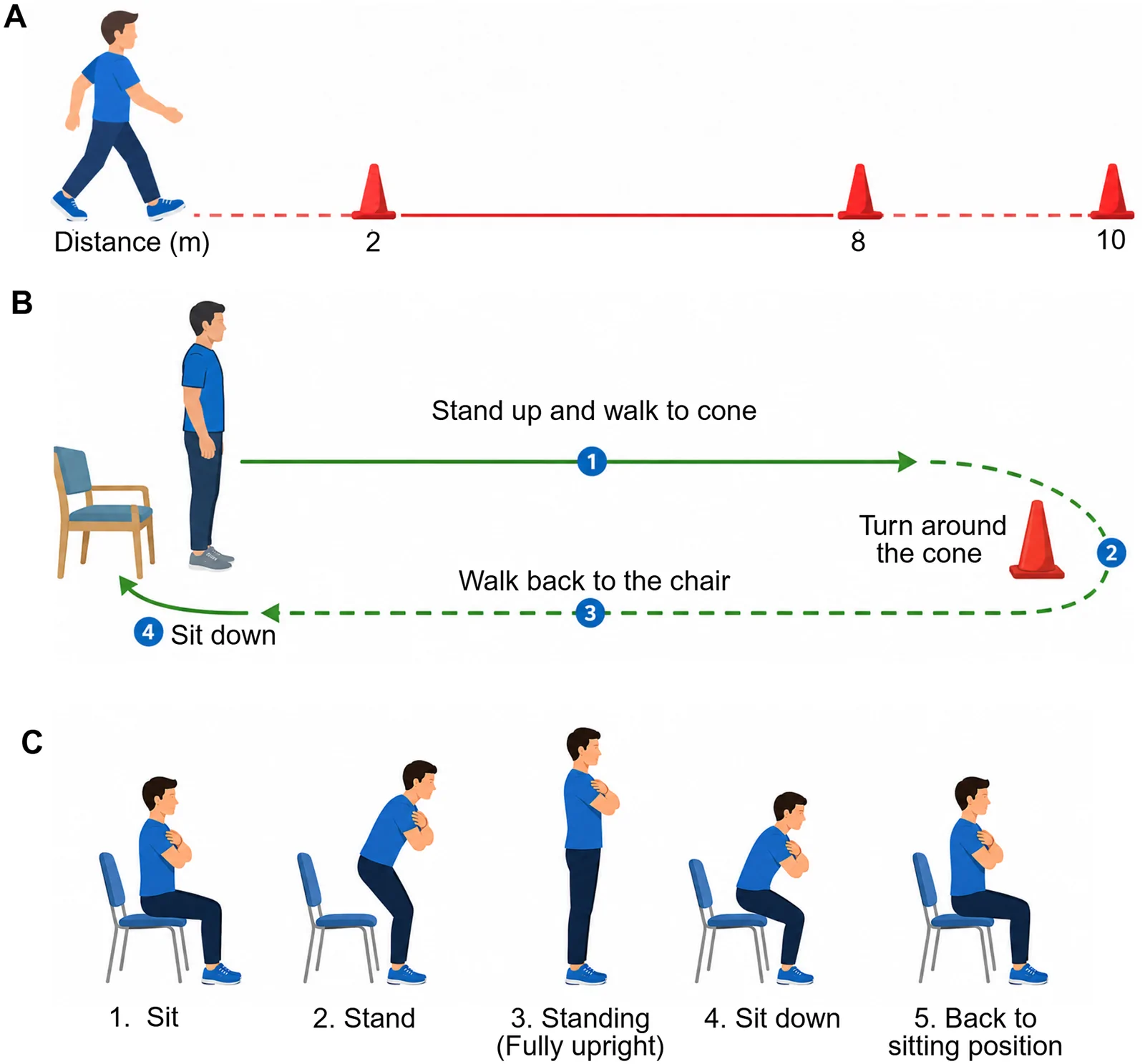
Schematic representation of the functional mobility assessments that will be performed in this study. **(A)** Ten-Meter Walk Test (10MWT): participants will walk along a 10-m walkway, and gait speed will be calculated over the central 6-meters section between the 2-meters and 8-meters markers to minimize acceleration and deceleration effects. **(B)** Timed Up and Go (TUG) test: participants will rise from a chair, walk to a cone, turn around, return to the chair, and sit down; the total time required to complete the task will be recorded. **(C)** Sit-to-Stand (STS) test: participants will complete repeated sit-to-stand cycles from a standard chair as quickly as possible. Performance characteristics, including time to completion, use of armrests, and balance during the task, will be observed and recorded. The illustrated sequences depict the procedures that will be used for each assessment.

### Timed Up and Go (TUG) Test

Functional mobility will be assessed using the Timed Up and Go (TUG) test, a widely used and reliable measure of dynamic balance, transitional movements, and overall mobility performance in individuals with neurological disorders [31]. TUG evaluates multiple functional tasks commonly affected in HSP, including sit-to-stand transfer, gait initiation, walking, turning, and stand-to-sit transition. Participants will begin seated in a standard armchair and, upon instruction, will stand, walk 3 meters at a maximum safe pace, turn 180°, return to the chair, take another turn and sit down (Fig 4B). The total time from initiating the rise from the chair to returning to a seated position after completing the walk will be recorded using a stopwatch. Participants will be permitted to use their customary assistive devices, orthoses, and footwear, which will be documented across assessment visits whenever possible. Three trials will be performed, with adequate rest provided between trials to minimize fatigue. The mean completion time across the three trials will be used for analysis. In addition to total completion time, qualitative observations including gait instability, balance loss, turning difficulty, use of upper-extremity support, and level of assistance required will be documented. For participants unable to complete the test independently, the reason for non-completion will be recorded.

### Modified Ashworth Scale (MAS)

Lower-extremity spasticity will be assessed using the Modified Ashworth Scale (MAS), a widely used clinically validated measure of resistance to passive movement in individuals with upper motor neuron disorders. The MAS grades muscle tone on a 6-point ordinal scale ranging from 0 (no increase in muscle tone) to 4 (affected part rigid in flexion or extension), with an additional grade of 1+ representing a catch followed by minimal resistance through less than half of the range of motion [32–34]. Spasticity assessments will be performed bilaterally by trained study personnel using standardized positioning and movement procedures. Muscle groups evaluated will include the hip adductors, hip flexors, knee extensors (quadriceps), knee flexors (hamstrings), and ankle plantarflexors (gastrocnemius-soleus complex). Assessment of ankle dorsiflexors will be performed when clinically indicated. In addition to MAS scores, the presence of clonus or muscle spasms, pain during passive movement, range-of-motion limitations, and contractures affecting assessment will be documented. For analysis, individual muscle-group scores and composite lower-extremity spasticity scores will be calculated to characterize changes in spasticity severity over the course of the intervention.

### Spastic Paraplegia Rating Scale (SPRS)

Disease severity will be assessed using the Spastic Paraplegia Rating Scale (SPRS), a validated HSP-specific clinical outcome measure [35]. The SPRS consists of 13 items assessing gait and mobility, transfers, lower-extremity spasticity and weakness, contractures, pain, and bladder and bowel dysfunction [35]. Transfer and lower-extremity function will include the sit-to-stand assessment (Fig 4C). Items are scored on a 5-point scale and summed to generate a total score of 0–52, with higher scores indicating greater disease severity. Assessments will be administered by trained study personnel using standardized procedures. Total SPRS scores will be used to quantify overall disease severity and to evaluate functional changes in response to the tSCS intervention.

## Secondary outcomes

### Six-Minute Walk Test (6MWT)

Walking endurance will be assessed using the Six-Minute Walk Test (6MWT), a validated measure of submaximal exercise capacity widely used in neurological and rehabilitation populations [36]. Participants will walk as far as possible for six minutes at a self-selected pace along a standardized indoor walkway and may slow down or rest as needed. Total distance walked (m) will be measured, with the number and duration of rest periods and reasons for early termination documented when applicable. Fatigue, pain, spasticity-related limitations, gait instability, and balance impairments will also be recorded.

### Isometric knee strength assessment

Lower-extremity muscle strength will be assessed using a Biodex System 4 Pro isokinetic dynamometer (Biodex Medical Systems, Shirley, NY, USA). Bilateral isometric knee extension and flexion strength will be evaluated using established testing procedures [37–39]. Participants will be secured to the dynamometer chair using the waist, contralateral thigh, and chest straps to isolate movement of the test limb [38]. Isometric knee extension and flexion strength testing will be performed while the participant is seated at 85° of hip flexion and 90° of knee flexion with the knee joint center aligned with the rotational axis on the dynamometer. Following two submaximal warm-up trials (performed at 50 and 75% effort, respectively), participants will perform three 7-second maximal voluntary isometric contractions (MVICs) for both knee extension and knee flexion. A minimum 30-second rest period will be provided between contractions to minimize fatigue. Participants will be instructed to exert maximal effort throughout each contraction while receiving standardized verbal encouragement from study personnel. Peak torque values (Nm) generated during knee extension and knee flexion will be recorded for each limb and used as primary indicators of lower-extremity muscle strength. Self-reported knee pain will be assessed during each MVIC using an 11-point numerical rating scale (0–10) [40].

### Three-dimensional gait analysis with wireless surface electromyography

Quantitative gait biomechanics will be assessed using an 11-camera three-dimensional motion capture system (Motion Analysis Corp., Santa Rosa, CA), synchronized wireless surface electromyography (EMG; Delsys Trigno, Delsys Inc., Boston, MA), two in-ground force plates (Bertec Corp., Columbus, OH), and timing gates (Brower Timing Systems, Draper, UT). Marker trajectories, ground reaction forces, and muscle activity will be collected synchronously using established procedures (Fig 5A-5B) [39,41]. Standardized footwear will be used to minimize footwear-related effects on joint kinetics [42]. Participants will walk at a comfortable, self-selected speed established during acclimation, with subsequent trials maintained within ±5% of this speed. A minimum of five successful trials, defined by consistent walking speed and a clean force-plate strike, will be collected. Spatiotemporal parameters and lower-extremity joint kinematics and kinetics will be derived from the motion capture and force-plate data. Wireless surface EMG sensors will be placed bilaterally over the vastus lateralis, vastus medialis, rectus femoris, medial gastrocnemius, and lateral gastrocnemius according to SENIAM guidelines [43]. A 5-second resting trial and maximal voluntary isometric contractions (MVICs) of the quadriceps and gastrocnemii will be obtained to normalize gait EMG activity. Quadriceps MVICs will be performed in a seated position at approximately 90° knee flexion, and gastrocnemius MVICs during a maximal unilateral heel raise with support as needed. EMG data will be processed using previously published methodology [44]. Knee pain will be assessed immediately before and after gait testing using an 11-point scale (0–10) [40].

**Fig 5:**
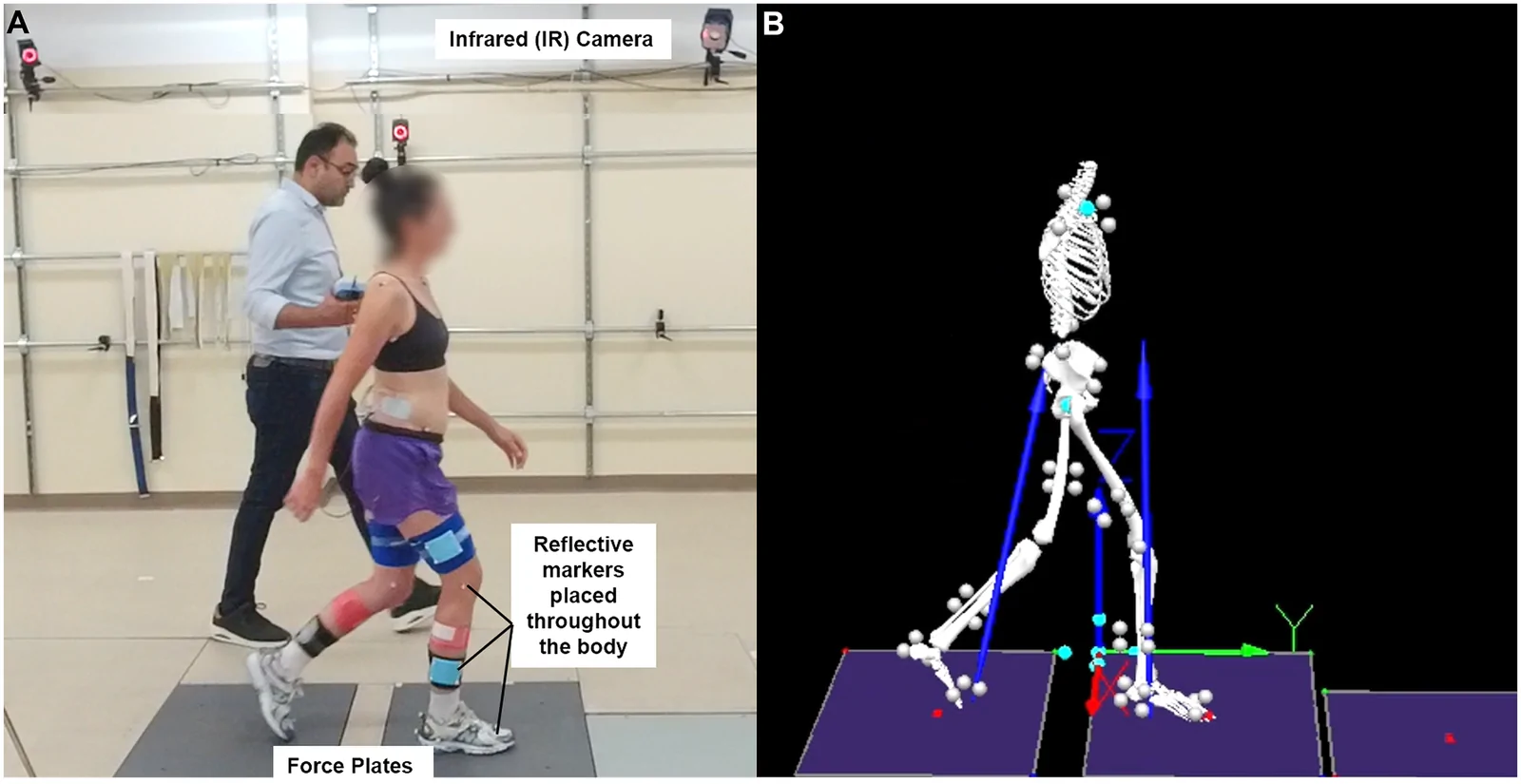
Three-dimensional gait biomechanics assessment. **(A)** Reflective markers positioned on anatomical landmarks are tracked by infrared cameras while the participant walks across embedded force plates. **(B)** Marker trajectories are reconstructed to generate a three-dimensional representation of the participant, enabling quantitative assessment of gait biomechanics.

### Self-notion and perception questionnaire in hereditary spastic paraplegia (HSP-SNAP)

Patient-reported disease burden will be assessed using the Self-Notion and Perception Questionnaire in Hereditary Spastic Paraplegia (HSP-SNAP), a disease-specific measure evaluating walking, stiffness, weakness, fatigue, balance, pain, endurance, and daily activities [45]. Participants will complete the HSP-SNAP at each assessment visit, with responses scored as previously described [45], with higher scores reflecting greater symptom burden and perceived functional impairment. Total scores will be used to evaluate changes in patient-perceived symptoms and functional impact following tSCS.

### Stair climb test

Functional lower-extremity performance will be assessed using a timed Stair Climb Test [46]. Participants will ascend and descend a standardized flight of stairs as quickly and safely as possible, using customary assistive devices, orthoses, or handrail support as needed. A single trial will be performed, with total completion time and any assistance required recorded. Testing conditions will be maintained consistently across assessment visits whenever possible.

## Ancillary assessments

### Neurogenic Bladder Symptom Score (NBSS)

Lower urinary tract (LUT) function will be assessed using the NBSS, a validated patient-reported measure of neurogenic urinary symptoms and their impact on quality of life [47,48]. The NBSS assesses urinary incontinence, storage and voiding symptoms, consequences of LUT dysfunction, and bladder-related quality of life. Responses will be scored using established guidelines, with higher scores indicating greater symptom burden. Total and domain-specific scores will be used to evaluate changes in LUT symptoms and explore potential autonomic effects of tSCS.

### Neurogenic Bowel Dysfunction Score (NBDS)

Bowel function will be assessed using the NBDS, a validated patient-reported measure of neurogenic bowel symptom severity [49]. The NBDS assesses bowel movement frequency, duration of bowel care, medication use, digital evacuation, fecal incontinence, autonomic symptoms, bowel-related complications, and satisfaction with bowel management. Responses will be scored using established procedures, with higher scores indicating greater dysfunction. Total scores will be used to evaluate changes in bowel symptoms over the study period and explore potential effects of tSCS on autonomic function.

## Exploratory assessments

### Biomarker and metabolomic profiling

Metabolomic and lipidomic profiling are particularly relevant to HSP because many HSP-causing genes encode proteins involved in lipid biosynthesis, membrane trafficking, and organelle dynamics [50–53]. Disruption of these pathways may impair membrane integrity and energy homeostasis in the long corticospinal axons primarily affected in HSP, contributing to the characteristic length-dependent axonopathy [51,52]. HSP is increasingly recognized as involving altered lipid homeostasis, mitochondrial dysfunction, impaired cellular metabolism, and age-related biological changes [54]. To investigate these processes and their potential relationship with treatment response, approximately 10 mL of venous blood will be collected at baseline (Week 1), mid-intervention (Week 4), post-intervention (Week 8), and follow-up (Week 16). Samples will be processed within 60 minutes by centrifugation at 1000 × g for 10 minutes at 4 °C, aliquoted, and stored at −80 °C until analysis. Plasma will undergo targeted metabolomic and lipidomic profiling using liquid chromatography-tandem mass spectrometry (LC-MS/MS), encompassing pathways related to energy metabolism, amino acid and nucleotide metabolism, redox balance, mitochondrial function, and lipid homeostasis. Exploratory analyses will examine associations between metabolic signatures, disease severity, functional impairment, and responsiveness to tSCS.

Complementary protein biomarkers will assess axonal and neuronal injury, glial activation, and neuroinflammation. These will include neurofilament light chain (NfL), the most extensively studied fluid biomarker in HSP to date [55]; brain-derived tau (BD-tau) and related tau species as markers of neuronal injury [56]; and glial fibrillary acidic protein (GFAP), soluble triggering receptor expressed on myeloid cells 2 (sTREM2), and selected inflammatory cytokines (e.g., IL-1β, IL-2, IL-6, and TNF-α) [57–59]. Big tau, a high-molecular-weight tau isoform enriched in long-projecting neurons, including those of the autonomic nervous system [54,57], will also be explored. Its extended projection domain has been proposed to support axonal transport and resistance to protein aggregation [57], suggesting potential utility as a complementary marker of axonal integrity. Collectively, these exploratory, hypothesis-generating analyses will examine relationships between molecular profiles and gait, spasticity, muscle strength, disease severity, autonomic dysfunction, patient-reported outcomes, and response to tSCS.

## Statistical analysis

All analyses will be conducted using GraphPad Prism. Participant characteristics, feasibility, adherence, retention, and safety will be summarized descriptively using means and standard deviations or medians and interquartile ranges for continuous variables, and frequencies and percentages for categorical variables. Clinical, functional, patient-reported, biomechanical, and biomarker outcomes will be evaluated using within-subject analyses. Acute stimulation effects (OFF vs ON) will be assessed using paired t-tests or Wilcoxon signed-rank tests, as appropriate. Longitudinal changes across baseline, mid-intervention, post-intervention, and follow-up will be evaluated using mixed-effects models, with time as a fixed effect and participant as a random effect. For outcomes assessed under both stimulation conditions, stimulation condition (OFF vs ON) and the stimulation-by-time interaction will also be included as fixed effects. Effect sizes and 95% confidence intervals (CI) will be reported. The Modified Ashworth Scale (MAS) will be evaluated using composite and muscle-specific approaches. Exploratory treatment response will be defined a priori as an improvement of ≥0.1 m/s in 10MWT gait speed and/or a ≥ 1-point reduction in MAS score. Responder rates and potential associations with baseline clinical characteristics will be explored descriptively and using univariate analyses, given the pilot sample size. Exploratory biomarker analyses will assess associations between changes in metabolomic, lipidomic, and protein biomarkers and clinical outcomes using Pearson or Spearman correlations, as appropriate. Missing data will be summarized by outcome and time point. Statistical significance will be set at α = 0.05, with interpretation emphasizing effect sizes, 95% CI, feasibility, and biological plausibility.

## Safety monitoring and adverse events

Participant safety will be monitored throughout the study. Before each stimulation session, changes in medical status, medications, or health conditions will be assessed, and skin integrity at electrode sites will be examined before and after stimulation. Participants will be monitored for discomfort, skin irritation, autonomic symptoms, dizziness, fatigue, pain, and other adverse effects. All adverse events (AEs) will be documented and evaluated by the study investigators. If necessary, stimulation may be adjusted, temporarily suspended, or discontinued. Anticipated risks are minimal and include transient skin irritation, electrode-site discomfort, muscle soreness, fatigue, or sensory changes. Unanticipated problems will be reported according to institutional and regulatory requirements. Safety outcomes will be summarized by AE frequency and severity, intervention adherence, session completion, and study retention.

## Data management

All study data will be collected using standardized case report forms and stored in secure, password-protected institutional databases. Participants will be assigned unique study identification numbers, with identifiable information stored separately and accessible only to authorized study personnel. Data quality will be maintained through routine review and verification of data entry. Biological samples will be coded and handled according to institutional procedures. Video recordings and photographs captured during assessments will be securely stored, and any images or videos used for presentations or publications will be de-identified by masking facial features and other identifying information, as appropriate. De-identified data and biospecimens may be shared with collaborators in accordance with IRB approval and participant consent.

## Ethics and dissemination

The study was approved by the University of Kentucky Institutional Review Board (IRB #109294) and registered at ClinicalTrials.gov (NCT07417943). Written informed consent will be obtained before study procedures, and the study will be conducted in accordance with the Declaration of Helsinki and applicable guidelines. Protocol modifications will undergo IRB review as required, and participant confidentiality will be maintained. Findings generated from the research conducted under this protocol will be disseminated through scientific meetings and peer-reviewed publications, with de-identified data shared in accordance with institutional policies and participant consent. Results will inform future controlled trials evaluating tSCS efficacy and mechanisms in HSP.

## Discussion

HSP remains a challenging neurodegenerative disorder for which treatment is largely limited to symptomatic management. Although pharmacological and rehabilitation interventions may improve spasticity and mobility, no currently available therapy alters disease progression [60]. Spinal neuromodulation has emerged as a promising strategy for neurological conditions characterized by impaired motor control and altered spinal excitability. By modulating spinal sensorimotor networks, tSCS may enhance the responsiveness of interneuronal and motor networks to residual descending inputs while reducing abnormal motor neuron excitability associated with spasticity, thereby improving motor control and mobility.

To our knowledge, this will be the first prospective clinical trial evaluating tSCS in individuals with HSP. Although preliminary evidence supports the feasibility of eSCS in HSP [13,14], the need for surgical implantation limits its broader clinical application. This study evaluates noninvasive tSCS using a multimodal framework integrating clinical, patient-reported, biomechanical, electrophysiological, and molecular outcomes. Quantitative gait analysis, EMG, strength testing, and autonomic assessments may provide objective measures of functional response beyond conventional clinical outcomes. Exploratory metabolomic, lipidomic, and neurodegenerative biomarker analyses will further investigate biological correlates of treatment response. Because HSP pathophysiology involves lipid metabolism, intracellular trafficking, and mitochondrial function [51,52,54,61], these analyses may capture biological changes not reflected by clinical measures alone. Integration of demographic, clinical, biomechanical, and molecular data may also help identify factors associated with tSCS responsiveness and inform future precision-neuromodulation strategies.

As a pilot study, the modest sample size and absence of a sham-controlled group will limit statistical power and causal inference, while the clinical and genetic heterogeneity of HSP may contribute to variability in treatment response. Nevertheless, the study will provide preliminary estimates of safety, feasibility, and treatment effects to inform larger controlled investigations.

## Supporting information

S1 Checklist SPIRIT checklist. Completed SPIRIT checklist for the study protocol.
(DOCX)

S1 Protocol Approved study protocol. Study protocol approved by the University of Kentucky Institutional Review Board.
(PDF)
