# Supplementary material for: Transcutaneous spinal cord stimulation for functional recovery in individuals with hereditary spastic paraplegia: Protocol for a pilot clinical trial
Source: PLoS One. 2026 Oct 15;21(10):e0359948. doi: 10.1371/journal.pone.0359948 (PMC13671537; doi:10.1371/journal.pone.0359948)
Supplement: Supplementary file 1 [file pone.0359948.s002.pdf]

IRB Approval
1/12/2026
IRB # 109294
IRB1

Protocol #: 109294
Short Title: Neuromodulation to Enhance Motor Function in HSP
Submitted Date: 12/23/25

PROTOCOL TYPE (VERSION 5)

0 unresolved comment(s)

IMPORTANT NOTE:

If you accidentally select the wrong IRB type or "Protocol Process Type" while your Initial Review (IR) application is in draft form (unsubmitted), you may change your selections. Please contact the Office of Research Integrity (ORI) at 859-257-9428, IRBsubmission@uky.edu, or request a consult to resolve any questions regarding your selections *prior* to submitting your Initial Review application.

If your **submitted IR application has been returned to you for requested revisions or additional information,** to streamline the review process **do not make changes** to your selections here **unless instructed to do so by the ORI/IRB.**

Changes to this section cannot be made after initial approval has been issued (the option is not available for MR or CR).

For guidance, see:

- Which IRB should review my research?
- Which Protocol Process Type?
- "Getting Started"

Which IRB

(•) Medical ( ) NonMedical

Protocol Process Type

( ) Exemption
( ) Expedited (Must be risk level 1)
(•) Full

The revised Common Rule expanded exemption certification category 4 for certain secondary research with identifiable information or biospecimens. The regulations no longer require the information or biospecimens to be existing. For more information see the Exemption Categories Tool.

PROJECT INFORMATION

0 unresolved comment(s)

Title of Project: (Use the exact title listed in the grant/contract application, if applicable).
If your research investigates any aspect of COVID-19, please include "COVID19" at the beginning of your Project Title and Short Title

Noninvasive Spinal Cord Neuromodulation to Enhance Motor Function in
Individuals with Hereditary Spastic Paraplegia

Short Title Description
Please use a few key words to easily identify your study - this text will be displayed in the Dashboard listing for your study.

Neuromodulation to Enhance Motor Function in HSP

Anticipated Ending Date of Research Project: 11/30/2027

Maximum number of human subjects (or records/specimens to be reviewed) 15

After approval, will the study be open to enrollment of new subjects or new data/specimen collection? (•) Yes ( ) No

Are you requesting that the UK IRB serve as the lead IRB for a multi-site study, **OR** that the UK IRB defer review to another IRB? [Click here for "IRB Reliance" help]
(•) Yes ( ) No

If "Yes," before completing your IRB application, fill out the Reliance Request Form and submit it to irbreliance@uky.edu.

PI CONTACT INFORMATION

0 unresolved comment(s)

Principal Investigator (PI) role for E-IRB access
The PI is a UK-affiliated individual holding primary responsibility on the research project with the following permissions on the E-IRB application:

1. Read;
2. write/edit;
3. receive communications; and
4. submit to the IRB (IR, CR, MR, Other Review*).

Non-UK individuals ("external") are not eligible for the role of Principal Investigator and cannot create a new application, nor will they be listed in the "Change Principal Investigator" search tool.

If research is being submitted to or supported by an extramural funding agency such as NIH, a private foundation or a pharmaceutical/manufacturing company, the PI listed on the grant application or the drug protocol must be listed as PI here.

Please fill in any blank fields with the appropriate contact information (gray shaded fields are not editable). Required fields left blank will be highlighted in pink after you click "Save".

To change home and work addresses, go to myUK and update using the Employee Self Service (ESS) portal. If name has changed, the individual with the name change will need to submit a 'Name Change Form' to the Human Resources Benefits Office for entering into SAP. The new name will need to be associated with the individual's Link Blue ID in SAP before the change is reflected in E-IRB. Contact the HR Benefits Office for additional information.

The Principal Investigator's (PI) contact information is filled in automatically based on who logged in to create the application.

If you are not the Principal Investigator, do NOT add yourself as study personnel.
To change the PI contact information on an application in Researcher edit status:

- click "Change Principal Investigator";
- search for the PI's name using the search feature;
- click "Select" by the name of the Principal Investigator, then "Save Contact Information".

You will automatically be added as study personnel with editing permissions to continue editing the application.

Change Principal Investigator:

First Name:	Rahul	Room# & Bldg:	800 Rose Street
Last Name:	Sachdeva	Speed Sort#:	40356
Middle Name			
Department:	Physical Medicine & Rehab - …	Dept Code:	7H951
PI's Employee/Student ID#:	12833750	Rank:	Assistant Professor
PI's Telephone #:	8592574888	Degree:	PhD
PI's e-mail address:	rahulsachdeva@uky.edu	PI's FAX Number:	
PI is R.N.	○ Yes ● No	HSP Trained:	Yes
		HSP Trained Date:	2/10/2025
		RCR Trained:	Yes

Do you, the PI/researcher, have a significant financial interest related to your responsibilities at the University of Kentucky (that requires disclosure per the UK administrative regulation 7:2)?

○ Yes ● No

RISK LEVEL

0 unresolved comment(s)

Indicate which of the categories listed below accurately describes this protocol

- ○ (Risk Level 1) Not greater than minimal risk
- ◉ (Risk Level 2) Greater than minimal risk, but presenting the prospect of direct benefit to individual subjects
- ○ (Risk Level 3) Greater than minimal risk, no prospect of direct benefit to individual subjects, but likely to yield generalizable knowledge about the subject's disorder or condition.
- ○ (Risk Level 4) Research not otherwise approvable which presents an opportunity to understand, prevent, or alleviate a serious problem affecting the health or welfare of subjects.

*"Minimal risk" means that the probability and magnitude of harm or discomfort anticipated in the research are not greater in and of themselves from those ordinarily encountered in daily life or during the performance of routine physical or psychological examination or tests.

Refer to UK's guidance document on assessing the research risk for additional information.

SUBJECT DEMOGRAPHICS **0 unresolved comment(s)**

Age level of human subjects: (i.e., 6 mths.; 2yrs., etc..) 18yrs. to above

Study Population:

Describe the characteristics of the subject population, including age range, gender, ethnic background and health status. Identify the criteria for inclusion and exclusion.

Provide the following information:

- A description of the subject selection criteria and rationale for selection in terms of the scientific objectives and proposed study design;
- A compelling rationale for proposed exclusion of any sex/gender or racial/ethnic group;
- Justification for the inclusion of vulnerable groups such as children, prisoners, adults with impaired consent capacity, or others who may be vulnerable to coercion or undue influence.

Please consider this FDA Guidance on Enrollment of Participants from Underrepresented Populations in Clinical Studies

This pilot study will target recruiting 15 individuals with genetically confirmed or clinically diagnosed HSP.
Inclusion Criteria:
• Age 18 years or above.
• Clinical diagnosis of hereditary spastic paraplegia (genetic confirmation if available).
• Stable medications for spasticity and other neurologic symptoms for =4 weeks prior to enrollment.
• Able to participate in study visits and assessments with or without assistive devices.
• If ambulatory: able to walk at least 10 meters with or without an assistive device.
• If wheelchair user: able to perform seated mobility tasks and transfers required for assessments.
• Capacity to provide informed consent and follow study procedures, with an ability to communicate and understand instructions in English.
Exclusion Criteria:
• Implanted electronic devices (e.g., pacemaker, deep brain stimulator, intrathecal pumps).
• Severe cardiopulmonary disease that would make participation unsafe.
• Open skin lesions or severe dermatologic conditions at electrode sites.
• Pregnancy or plans to become pregnant during the intervention period.
• Diagnosed with Primary Lateral Sclerosis (PLS) or another neurological condition that affects walking, such as stroke, multiple sclerosis (MS), or a recent surgery on legs.
• Unable to participate in basic movement or mobility assessments, even with their usual mobility device (such as a wheelchair, walker, or cane).
Note: People who use wheelchairs or other mobility aids can participate if they can complete the study's mobility assessments in their usual way.
• Cognitive or psychiatric conditions that make it difficult to give informed consent or follow study instructions.
• Diagnosed with Urinary Tract Infection (UTI), either acute or ongoing, before or at the time of study enrollment.
• Diagnosed with epilepsy.
• Participation in another interventional clinical trial that could affect mobility or spasticity during the study.
• A recent change (within the last 4 weeks) in medications or treatments that affect spasticity or movement (for example: baclofen, tizanidine, botulinum toxin injections).
• Expect to start or change treatments for spasticity or mobility during the study period.
• Any condition judged by the investigator to pose excess risk or confound outcomes.

Attachments

Indicate the targeted/planned enrollment of the following members of minority groups and their subpopulations. Possible demographic sources: Kentucky State Census, Kentucky Race/Ethnic Table, Kentucky Population Data.
(Please note: The IRB will expect this information to be reported at Continuation Review time for Pre-2019 FDA-regulated Expedited review and Full review applications):

Participant Demographics

	Cisgender Man	Cisgender Woman	TGNB/TGE	Unknown/Not Reported
American Indian/Alaskan Native:				
Asian:				
Black/African American:				
Latinx:				
Native Hawaiian/Pacific Islander:				
White:				
American Arab/Middle Eastern/North African:				
Indigenous People Around the World:				

More than One Race:				
Unknown or Not Reported:				

If unknown, please explain why:

Demographic information will be updated at the continuation review

Indicate the categories of subjects and controls to be included in the study. You may be required to complete additional forms depending on the subject categories which apply to your research. If the study does not involve direct intervention or direct interaction with subjects, (e.g., record-review research, outcomes registries), do not check populations which the research does not specifically target. For example: a large record review of a diverse population may incidentally include a prisoner or an international citizen, but you should not check those categories if the focus of the study has nothing to do with that status.

Check All That Apply (at least one item must be selected)

- [ ] Children (individuals under age 18)
- [ ] Wards of the State (Children)
- [ ] Emancipated Minors
- [ ] Students
- [ ] College of Medicine Students
- [ ] UK Medical Center Residents or House Officers
- [ ] Impaired Consent Capacity Adults
- [ ] Pregnant Women/Neonates/Fetal Material
- [ ] Prisoners
- [ ] Non-English Speaking (translated long or short form)
- [ ] International Citizens
- [x] Normal Volunteers
- [ ] Military Personnel and/or DoD Civilian Employees
- [x] Patients
- [ ] Appalachian Population

ADDITIONAL INFORMATION:

Please visit the IRB Survival Handbook for more information on:

- Children/Emancipated Minors
- Students as Subjects
- Prisoners
- Impaired Consent Capacity Adults
- Economically or Educationally Disadvantaged Persons

Other Resources:

- UKMC Residents or House Officers [see requirement of GME]
- Non-English Speaking [see also the E-IRB Research Description section on this same topic]
- International Citizens [DoD SOP may apply]
- Military Personnel and/or DoD Civilian Employees

Assessment of the potential recruitment of subjects with impaired consent capacity (or likelihood):

☐ Check this box if your study does NOT involve direct intervention or direct interaction with subjects (e.g., record-review research, secondary data analysis). If there is no direct intervention/interaction you will not need to answer the impaired consent capacity questions.

Does this study focus on adult subjects with any conditions that present a high *likelihood* of impaired consent capacity or *fluctuations* in consent capacity? (see examples below)

○ Yes ◉ No

If Yes and you are not filing for exemption certification, go to "Form T", complete the form, and attach it using the button below.

Examples of such conditions include:

- Traumatic brain injury or acquired brain injury
- Severe depressive disorders or Bipolar disorders
- Schizophrenia or other mental disorders that involve serious cognitive disturbances
- Stroke
- Developmental disabilities
- Degenerative dementias
- CNS cancers and other cancers with possible CNS involvement
- Late stage Parkinson's Disease
- Late stage persistent substance dependence
- Ischemic heart disease
- HIV/AIDS
- COPD
- Renal insufficiency
- Diabetes
- Autoimmune or inflammatory disorders
- Chronic non-malignant pain disorders
- Drug effects
- Other acute medical crises

Attachments

INFORMED CONSENT/ASSENT PROCESS/WAIVER

1 unresolved comment(s)

For creating your informed consent attachment(s), please download the most up-to-date version listed in "All Templates" under the APPLICATION LINKS menu on the left, and edit to match your research project.

Additional Resources:

- Informed Consent/Assent Website
- Waiver of Consent vs. Waiver of Signatures
- Sample Repository/Registry/Bank Consent Template

Consent/Assent Tips:

- If you have multiple consent documents, be sure to upload each individually (not all in a combined file).
- If another site is serving as the IRB for the project, attach the form as a “Reliance Consent Form” so the document will not receive a UK IRB approval stamp; the reviewing IRB will need to stamp the consent forms.
- Changes to consent documents (e.g., informed consent form, assent form, cover letter, etc...) should be reflected in a 'tracked changes' version and uploaded separately with the Document Type "Highlighted Changes".
- It is very important that only the documents you wish to have approved by the IRB are attached; DELETE OUTDATED FILES -- previously *approved* versions will still be available in Protocol History.
- Attachments that are assigned a Document Type to which an IRB approval stamp applies will be considered the version(s) to be used for enrolling subjects once IRB approval has been issued.
  Document Types that do NOT get an IRB approval stamp are:
  - "Highlighted Changes",
  - "Phone Script", and
  - "Reliance Consent Form",
  - "Sponsor's Sample Consent Form".

How to Get the Section Check Mark

1. You must:

   a) provide a response in the text box below describing how investigators will obtain consent/assent, and
   b) check the box for at least one of the consent items and/or check mark one of the waivers

2. If applicable attach each corresponding document(s) **as a read-only PDF**.
3. If you no longer need a consent document approved (e.g., closed to enrollment), or, the consent document submitted does not need a stamp for enrolling subjects (e.g., umbrella study, or sub-study), only select "Stamped Consent Doc(s) Not Needed".
4. After making your selection(s) be sure to scroll to the bottom of this section and SAVE your work!

Check All That Apply

- ☐ Informed Consent Form (and/or Parental Permission Form and/or translated short form)
- ☐ Assent Form
- ☐ Cover Letter (for survey/questionnaire research)
- ☐ Phone Script
- ☑ Informed Consent/HIPAA Combined Form
- ☐ Debriefing and/or Permission to Use Data Form
- ☐ Reliance Consent Form
- ☐ Sponsor's sample consent form for Dept. of Health and Human Services (DHHS)-approved protocol
- ☐ Stamped Consent Doc(s) Not Needed

Attachments

Attach Type	File Name
Informed Consent/HIPAA Combined Form	Clean_Informed-Consent-form_SCS-HSP_Sachdeva_Rev1.pdf
InformedConsent-Highlighted/Tracked_Changes	Highlighted_Informed-Consent-form_SCS-HSP_Sachdeva_Rev1.pdf

Informed Consent Process:

Using active voice, in the text box below, describe how investigators will obtain consent/assent. Include:

- the circumstances under which consent will be sought and obtained
- the timing of the consent process (including any waiting period between providing information and obtaining consent)
- who will seek consent
- how you will minimize the possibility of coercion or undue influence
- the method used for documenting consent
- if applicable, who is authorized to provide permission or consent on behalf of the subject
- if applicable, specific instruments or techniques to assess and confirm potential subjects' understanding of the information

Will electronic consent form/process be utilized on-site or remotely for this study?

(•) Yes ( ) No

If yes, in addition to addressing the above bullet points, describe the e-consent method and platform, including any hyperlinks, videos, or enhancements used to convey information, if applicable. Attach a representation of the e-consent with signature fields. For guidance, see the ORI E-Consent web page.

Note: all individuals authorized to obtain informed consent should be designated as such in the E-IRB "Study Personnel" section of this application.

Special considerations may include:

- Obtaining consent/assent for special populations such as children, prisoners, or people with impaired decisional capacity
- *Research Involving Emancipated Individuals*
  If you plan to enroll some or all prospective subjects as emancipated, consult with UK legal counsel **prior to submitting this application to the IRB**. Include research legal counsel's recommendations in the "Additional Information" section as a separate document.
- *Research Involving Non-English Speaking Subjects*
  For information on inclusion of non-English speaking subjects, or subjects from a foreign culture, see IRB Application Instructions for Recruiting Non-English Speaking Participants or Participants from a Foreign Culture.
- *Research Repositories*
  If the purpose of this submission is to establish a research repository describe the informed consent process. For guidance regarding consent issues, process approaches, and sample language see the Sample Repository/Registry/Bank Consent Template.

Each prospective participant will be given adequate time to read the informed consent document and discuss the study with the research team. A member of the study team will review the consent form with the participant, answer all study-related questions, and confirm understanding of study procedures, risks, benefits, and the voluntary nature of participation. Written informed consent will be obtained prior to performing any study-specific procedures. After the consent form is signed, a copy of the signed informed consent document will be provided to the participant.

Participants may complete the informed consent process in one of two ways:
(1) by attending an in-person visit, during which the consent form will be reviewed and signed, or
(2) by receiving the IRB-approved consent form via email. Participants who receive the consent form electronically will be given sufficient time to review the document, ask questions of the Principal Investigator or study personnel, and may return the signed consent form via email or bring the signed copy to their first baseline assessment visit (visit 2). This option is intended to reduce the number of required study visits for participants who live at a greater distance from the study site.
Written informed consent will be obtained prior to the initiation of any study-specific procedures. After the consent form is signed, a copy of the signed informed consent document will be provided to the participant.

To minimize the possibility of coercion or undue influence, only individuals explicitly designated in the E-IRB "Study Personnel" section as authorized to obtain informed consent will conduct the consent process.
Specifically, consent will be obtained only by:
Dr. Rahul Sachdeva
Dr. Sara Salles
Dr. Zakari Dymock
Dr. Susan McDowell
Dr. Vivek Pandey
Ms. Makyra Ross

The consent discussion will occur in a private setting (or other appropriate confidential location), and participants will be reminded that participation is voluntary, they may decline to participate or withdraw at any time without penalty or loss of benefits to which they are otherwise entitled, and they are free to ask questions at any time.
Participants will be provided the Principal Investigator's contact information during the consent process and will also be informed they may contact the University of Kentucky Office of Research Integrity (ORI) at 859-257-9428 or toll free at 1-866- 400-9428 with any concerns.

[ ] Request for Waiver of Informed Consent Process

If you are requesting IRB approval to waive the requirement for the informed consent process, or to alter some or all of the elements of informed consent, complete, Section 1 and Section 2 below.

Note: The IRB does not approve waiver or alteration of the consent process for greater than minimal risk research, except for planned emergency/acute care research as provided under FDA regulations. Contact ORI for regulations that apply to single emergency use waiver or acute care research waiver (859-257-9428).

SECTION 1.

Check the appropriate item:

☐ I am requesting a waiver of the requirement for the informed consent process.

☐ I am requesting an alteration of the informed consent process.

If you checked the box for this item, describe which elements of consent will be altered and/or omitted, and justify the alteration.

SECTION 2.

Explain how each condition applies to your research.

a) The research involves no more than minimal risk to the subject.

b) The rights and welfare of subjects will not be adversely affected.

c) The research could not practicably be carried out without the requested waiver or alteration.

d) Whenever possible, the subjects or legally authorized representatives will be provided with additional pertinent information after they have participated in the study.

e) If the research involves using or accessing identifiable private information or identifiable biospecimens, the research could not practicably be carried out without using such information or biospecimens in an identifiable format.

- Private information/specimens are "identifiable" if the investigator may ascertain the identity of the subject or if identifiers are associated with the information (e.g., medical records). This could be any of the 18 HIPAA identifiers including dates of service.
- If not using identifiable private information or identifiable biospecimens, insert N/A below.

Request for Waiver of Signatures

If you are requesting IRB approval to waive the requirement for signatures on informed consent forms, **your research activities must fit into one of three regulatory options**:

1. The only record linking the participant and the research would be the consent document, and the principal risk would be potential harm resulting from a breach of confidentiality (e.g., a study that involves participants who use illegal drugs).
2. The research presents no more than minimal risk to the participant and involves no procedures for which written consent is normally required outside of the research context (e.g., a cover letter on a survey, or a phone script).
3. The participant (or legally authorized representative) is a member of a distinct cultural group or community in which signing forms is not the norm, the research presents no more than minimal risk to the subject, and there is an appropriate alternative mechanism for documenting that informed consent was obtained.

Select the option below that best fits your study.

*If the IRB approves a waiver of signatures, participants must still be provided oral or written information about the study. To ensure you include required elements in your consent document, use the **Cover Letter Template** as a guide. There is an English and a Spanish version.*

Option 1

Describe how your study meets these criteria:

a) The only record linking the participant and the research would be the consent document:

b) The principal risk would be potential harm resulting from a breach of confidentiality (i.e., a study that involves subjects who use illegal drugs).

Under this option, each participant (or legally authorized representative) must be asked whether (s)he wants to sign a consent document; if the participant agrees to sign a consent document, only an IRB approved version should be used.

Option 2

Describe how your study meets these criteria:

a) The research presents no more than minimal risk to the participant:

b) Involves no procedures for which written consent is normally required outside of the research context (i.e. a cover letter on a survey, or a phone script):

Option 3

Describe how your study meets these criteria:

a) The subject (or legally authorized representative) is a member of a distinct cultural group or community in which signing forms is not the norm.

b) The research presents no more than minimal risk to the subject.

c) There is an appropriate alternative mechanism for documenting that informed consent was obtained.

STUDY PERSONNEL **0 unresolved comment(s)**

Do you have study personnel who will be assisting with the research?
After selecting 'Yes' or 'No' you must click the 'Save Study Personnel Information' button.

(•) Yes ( ) No

Manage Study Personnel

Identify other study personnel assisting in research project:

- The individual listed as PI in the 'PI Contact Information' section should NOT be added to this section.
- If the research is required for a University of Kentucky academic program, the faculty advisor is also considered study personnel and should be listed below. ***Residents and students who are PI's are encouraged to designate the faculty advisor or at least one other individual as a contact with an editor role (DP).***
- Role: DP = Editor (individual can view, navigate, and edit the application for any review phase (IR, CR/FR, MR) or 'Other Review", and submit Other Reviews on behalf of the PI.)
- Role: SP = Reader (individual can view and navigate through the currently approved application only.)

To add an individual via the below feature:

- Search for personnel;
- Click "select" by the listing for the person you want to add;
- For each person, specify responsibility in the project, whether authorized to obtain informed consent, AND denote who should receive E-IRB notifications (contact status).

NOTE: Study personnel must complete human subject protection (HSP) and Responsible Conduct of Research (RCR) training before implementing any research procedures. For information about training requirements for study personnel, visit UK's HSP FAQ page, the RCR Home page, or contact ORI at 859-257-9428. If you have documentation of current HSP training other than that acquired through UK CITI, you may submit it to ORI (HSPTrainingSupport@uky.edu) for credit.

Study personnel assisting in research project:

Last Name	First Name	Responsibility In Project	Role	A C	Contact	Degree	StatusFlag	(HSP)	(HSP)Date	(RCR)	Removed?	Last Updated	SFI	Active
Dymock	Zakari	Consultant/Advisor	SP	Y	N	MD	P	Y	02/27/2023	Y	N	12/23/2025	N	Y
Elliott	Holly	Data Collection	SP	N	N		P	Y	05/08/2025	Y	N	01/16/2026	N	Y
Pandey	Vivek	Co-Investigator	DP	Y	Y	PhD	P	Y	06/25/2025	Y	N	12/23/2025	N	Y
Patel	Samirkumar	Co-Investigator	SP	N	N	PhD	P	Y	01/15/2026	Y	N	12/23/2025	N	Y
Ross	Makyra	Data Analysis/Processing	SP	Y	Y		P	Y	06/10/2025	Y	N	12/23/2025	N	Y
Samaan	Michael	Co-Investigator	SP	Y	Y		P	Y	06/03/2024	Y	N	12/23/2025	N	Y
Shahid Salles	Sara	Medical Supervisor	SP	Y	Y	DO	P	Y	04/29/2025	Y	N	12/23/2025	N	Y
Whitaker	Hannah	Data Collection	SP	N	N		P	Y	06/06/2023	Y	N	01/16/2026	N	Y
McDowell	Susan	Consultant/Advisor	SP	Y	N		P	Y	05/08/2024	Y	Y	12/23/2025	N	Y

RESEARCH DESCRIPTION

0 unresolved comment(s)

You may attach a sponsor's protocol pages in the "Additional Information" section and refer to them where necessary in the Research Description. However, each prompt that applies to your study should contain at least a summary paragraph.

Pro Tips:

- **Save your work often to avoid losing data.**
- **Use one of the attachment buttons in this section or under the Additional Information section to include supplemental information with your application. During the document upload process, you will be able to provide a brief description of the attachment.**

Background

Include a brief review of existing literature in the area of your research. You should identify gaps in knowledge that should be addressed and explain how your research will address those gaps or contribute to existing knowledge in this area. For interventional research, search PubMed and ClinicalTrials.gov for duplicative ongoing and completed trials with same condition and intervention(s).

More than 90 gene mutations are known to cause HSP, primarily by disrupting key cellular processes that lead to length-dependent degeneration of the corticospinal tracts and dorsal column axons. In complex forms of HSP, additional pathways such as the spinocerebellar tracts may be affected, contributing to neurological features like ataxia and sensory deficits.
Despite the considerable genetic and clinical heterogeneity of HSP, a unifying hallmark is progressive, debilitating spasticity and weakness of the lower limbs. Urinary urgency is common, and in more complex cases, individuals may also experience fecal incontinence, seizures, cognitive and speech decline, intellectual disability, dementia, muscle wasting, ataxia, and peripheral neuropathy. These symptoms result in worsening gait impairment, frequent falls, growing dependence on assistive devices or caregivers, and loss of independence. Although not life-threatening, HSP significantly impacts quality of life, often leading to unemployment and reduced social participation. While no comprehensive US or global economic evaluations of HSP have been conducted, a recent study in Germany estimated the mean annual cost of illness for individuals with HSP at €27,074 (~$29,800 USD), driven largely by expenses such as home modifications, mobility aids, and informal caregiving. Given an average disease duration exceeding two decades, the estimated lifetime economic burden surpasses $680,000 per patient.
Currently available treatment options only provide symptomatic relief and remain largely ineffective in addressing the underlying cause of disease. Medications such as baclofen, tizanidine, and botulinum toxin can reduce spasticity, but their effectiveness is variable, and side effects such as sedation and muscle weakness are common. Physical and occupational therapy are essential for maintaining mobility and preventing contractures, yet they cannot alter disease progression, and benefits diminish without ongoing intervention. In severe or refractory cases, surgical options like intrathecal baclofen pumps, selective dorsal rhizotomy, or orthopedic procedures may be considered, but these carry risks of infection, device malfunction, and irreversible loss of function. Given the limitations of current standard of care, there is a compelling rationale to explore alternative approaches to improve motor function and reduce spasticity in individuals with HSP.

Objectives

List your research objectives. Please include a summary of intended research objectives in the box below.

Primary Objective: Determine whether 8 weeks of tSCS improves motor function/mobility compared with baseline.
Secondary Objectives:
1. Examine changes in lower-limb strength, spasticity, gait/mobility performance, and patient-reported symptoms.
2. Characterize metabolic/biomarker changes associated with neuromodulation.
3. Assess persistence of effects 8 weeks after the stimulation period.

Study Design

Describe and explain the study design (e.g., observational, secondary analysis, single/double blind, parallel, crossover, deception, etc.).

- *Clinical Research*: Indicate whether subjects will be randomized and whether subjects will receive any placebo.
- *Community-Based Participatory Research*: If you are conducting community-based participatory research (CBPR), describe strategies for involvement of community members in the design and implementation of the study, and dissemination of results from the study.
- *Qualitative research*: Indicate ranges where flexibility is needed, if a fixed interview transcript is not available, describe interview topics including the most sensitive potential questions.
- *Research Repositories*: If the purpose of this submission is to establish a Research Repository (bank, registry) and the material you plan to collect is already available from a commercial supplier, clinical lab, or established IRB approved research repository, provide scientific justification for establishing an additional repository collecting duplicate material. Describe the repository design and operating procedures. For relevant information to include, see the UK Research Biospecimen Bank Guidance or the UK Research Registry Guidance.

Aim 1: To evaluate the effects of noninvasive tSCS on motor function in individuals with HSP.
Open-label, single-arm pilot clinical trial with repeated-measures assessment at baseline (Week 1), mid- intervention (Week 4), post-

intervention (Week 8), and follow-up (Week 16). Participants will undergo 16 tSCS sessions over 8 weeks (2 sessions/week). No sham control is used in this feasibility phase.
Primary outcomes will be the 10-Meter Walk Test (10MWT), which assesses gait speed, and the Modified Ashworth Scale (MAS) to evaluate spasticity. Secondary outcomes will include the 6- Minute Walk Test (6MWT) to assess endurance, the Spastic Paraplegia Rating Scale (SPRS), a clinical measure of disease severity, and HSP- Self Notion and Perception (HSP-SNAP) questionnaire.
Mechanistic outcomes will include three-dimensional (3D) gait analysis and quantitative measurement of lower- limb muscle strength.
Exploratory outcomes will assess LUT and bowel function using the NBSS and Neurogenic Bowel Dysfunction Score (NBDS), along with plasma-based metabolic profiling.
Aim 1a will assess the immediate effects of a single tSCS session. At the second visit, motor and spasticity assessments will be conducted first without stimulation to establish a baseline, and then again during active real- time tSCS to evaluate acute effects. These same assessments will be repeated during week 4 in the middle of the study and at the end of the 8-week intervention to determine whether repeated tSCS enhances the spinal cord's responsiveness to real-time stimulation. Aim 1b will investigate the chronic and sustained effects of repeated tSCS over the 8-week intervention period. Assessments will be conducted in the absence of active stimulation at baseline, at 4 weeks (during the intervention to see the progress, if any), at 8 weeks (post-intervention and the primary efficacy endpoint), and at 16 weeks (8 weeks after the end of the intervention), to determine whether improvements in motor function are maintained over time.
Hypothesis: tSCS will significantly improve motor function in individuals with HSP, and that these effects will be maintained after the intervention.
Impact: This aim will establish preliminary evidence for the efficacy of noninvasive tSCS in improving motor function in individuals with HSP and provide critical proof-of-concept data to justify larger, controlled trials. Additionally, elucidating mechanistic changes through advanced gait analysis and muscle strength quantification will enhance understanding of how tSCS modulates spinal cord circuitry.
Aim 2: To identify predictors of motor response to tSCS in individuals with HSP.
We will develop a clinical prediction rule to identify individuals with HSP most likely to benefit from tSCS. Participants will be classified as responders based on pre-specified criteria (i.e., =0.1 m/s improvement in 10MWT or =1-point decrease in MAS score). Candidate predictors will include demographic and clinical variables such as age, sex, disease duration, genetic subtype, clinical phenotype (pure vs. complex), baseline gait speed, spasticity severity (MAS), lower limb strength, and functional mobility status (e.g., use of assistive devices). We will also evaluate interaction terms (e.g., phenotype × disease duration) to assess effect modification. Initial univariate analyses will evaluate associations between candidate predictors and responder status. Multivariable logistic regression will then be used to derive a prediction model, employing either backward stepwise selection based on Akaike Information Criterion (AIC) or Least Absolute Shrinkage and Selection Operator (LASSO) regularization, depending on the sample size and degree of predictor collinearity. The model's discrimination will be assessed by the area under the receiver operating characteristic curve (AUROC), with calibration evaluated using Hosmer–Lemeshow tests and calibration plots. Internal validation will be performed via 5-fold cross-validation and bootstrapping to ensure model stability.
From the final model, we will derive a preliminary clinical scoring system to stratify individuals by likelihood of response, with the understanding that this will require external validation in future larger studies. The scoring tool will be internally validated and evaluated across clinically meaningful thresholds.
As a complementary exploratory analysis, we will use multivariable linear regression to model the magnitude of change in continuous motor outcomes (e.g., gait speed, SPRS, muscle strength), allowing a more nuanced view of predictors of motor improvement. This continuous modeling will also help identify potential effect modifiers and inform mechanistic hypotheses regarding tSCS response variability.
Hypothesis: Baseline demographic and clinical characteristics will predict individual motor response to tSCS in people with HSP.
Impact: The prediction model will guide future personalized stimulation protocols, refine patient selection criteria, and support targeted trial designs of subgroups most likely to benefit from tSCS therapy.

Attachments

Subject Recruitment Methods & Advertising

Describe how the study team will identify and recruit subjects. Please consider the following items and provide additional information as needed so that the IRB can follow each step of the recruitment process.

- How will the study team identify potential participants?
- Who will first contact the potential subjects, and how?
- Will you use advertisements? If so, how will you distribute those?
- How and where will the research team meet with potential participants?
- If applicable, describe proposed outreach programs for recruiting women, minorities, or disparate populations.
- How you will minimize undue influence in recruitment?
- Attach copies of all recruiting and advertising materials (emails, verbal scripts, flyers, posts, messages, etc.).

For additional information on recruiting and advertising:

- IRB Application Instructions - Advertisements
- PI Guide to Identification and Recruitment of Human Subjects for Research

Potential participants will be identified using multiple complementary sources to ensure feasibility for this rare disease. These sources will include:
1. Clinical records/databases within UK HealthCare/PM&R and Cardinal Hill Rehabilitation Hospital (CHRH), including an established HSP database of >40 individuals maintained through the CHRH catchment area and clinician collaborators.
2. Clinician referral, including referrals from study co-investigators (e.g., Dr. Sara Salles) and other PM&R/Neurology providers who care for individuals with HSP.
3. Community and foundation connections, including individuals who previously expressed interest following study team presentations

to the Spastic Paraplegia Foundation (SPF) community (e.g., SPF Talks webinar).
4. Online and community-based organizations, including HSP advocacy groups and registries, where permitted.

Importantly, recruitment will be inclusive of people with diverse mobility abilities, including those who walk with or without assistive devices and those who use wheelchairs for some or all mobility.

Yes. IRB-approved advertisements (flyers, email scripts, and brief messages) will be used and distributed through:
1. CHRH/UK clinic postings in PM&R and affiliated neurology or rehabilitation spaces.
2. Online channels such as lab/department websites and social media posts approved by IRB.
3. Community-based organizations and registries, including SPF communication outlets and other HSP advocacy groups where allowed.
4. Direct follow-up to prior interested individuals who contacted the lab after SPF Talks or related engagement.

For individuals identified or contacted through neutral or portal messaging, a single follow-up neutral message may be sent if there is no initial response. No further contact will be made if the individual does not respond.

Potential participants will meet with the study team:

1. In person at the UK PM&R Research Lab located within CHRH for screening and informed consent, in a private setting.
2. Remotely (phone/video) for pre-screening and initial questions when appropriate, prior to scheduling an in-person screening visit.
Participants will be given adequate time to review consent documents, ask questions, and decide without pressure.

Because HSP is a rare condition, broad community outreach is essential. To promote equitable enrollment, the team will:
1. Recruit through both clinical systems and community/foundation networks, which helps reach individuals who may not be seen frequently in tertiary centers.
2. Use accessible, lay-language materials and inclusive imagery/language welcoming a wide range of functional abilities, including wheelchair users.
3. Offer flexible scheduling and accessibility supports to reduce barriers for participants with transportation or mobility limitations.
Enrollment will be monitored to ensure no group is unintentionally excluded.

To minimize undue influence:
• All recruitment materials and conversations will emphasize that participation is voluntary and that choosing not to participate will not affect current or future care.
• Initial contact with clinic-identified individuals will be made in a non-pressuring way (e.g., clinician introduction or neutral letter/portal message) and will invite the individual to contact the study team if interested.
• No claims of guaranteed benefit will be made. Recruitment will describe potential benefits and risks in balanced terms.
• compensation is offered and it will be presented as reimbursement for time/effort, not as a benefit, and will be prorated if a participant withdraws early.

Attachments

Attach Type	File Name
Advertising	Recruitment and Advertising materials.pdf
Advertising	HSP Flyer.pdf

Research Procedures

Describe how the research will be conducted.

- What experience will study participants have?
- What will study participants be expected to do?
- How long will the study last?
- Outline the schedule and timing of study procedures.
- Provide visit-by-visit listing of all procedures that will take place.
- Identify all procedures that will be carried out with each group of participants.
- Describe deception and debrief procedures if deception is involved.

Differentiate between procedures that involve standard/routine clinical care and those that will be performed specifically for this research project. List medications that are explicitly forbidden or permitted during study participation.

Participants will take part in a structured, supervised research program at Cardinal Hill Rehabilitation Hospital (CHRH) – Physical Medicine & Rehabilitation (PM&R) Research Lab (The PM&R department and laboratory space is leased by the University of Kentucky and located within CHRH; however, CHRH itself will not be involved in the conduct of this study in any other capacity) and the Human Performance Laboratory/Biodynamics Lab at the UK Campus. After a baseline assessment at the Human Performance Laboratory at the UK Campus, participants will attend twice-weekly sessions of noninvasive transcutaneous spinal cord stimulation (tSCS) for 8 weeks at the PM&R Lab. Assessments at Week 1, 4, 8, and 16 will be conducted at the Human Performance Laboratory/Biodynamics lab at the UK Campus. Each stimulation visit will be monitored by trained research and clinical staff to ensure comfort and safety. Participants will receive stimulation while seated or (if able) during light mobility tasks. The study concludes with an end-of-treatment assessment and a single follow-up assessment visit 8 weeks later.

Participants will participate in a structured, supervised research program at the PM&R Lab and the Human Performance Laboratory/Biodynamics Lab on the UK Campus. After a baseline assessment at the Human Performance Laboratory at the UK Campus, participants will attend twice-weekly sessions of noninvasive transcutaneous spinal cord stimulation (tSCS) for 8 weeks at the PM&R Research Lab located in CHRH. Assessments at Week 1, 4, 8 and 16 will be conducted at the Human Performance Laboratory/Biodynamics Lab at the UK Campus. Each stimulation visit will be monitored by trained research and clinical staff to ensure comfort and safety. Participants will receive stimulation while seated or (if able) during light mobility tasks. The study concludes with an end-of-treatment assessment and a single follow-up assessment visit 8 weeks later.

Participants will be expected to:
• Attend all scheduled lab visits over the 16-week study period.
• Complete baseline, post-treatment, and follow-up assessments of motor function, mobility, strength, and spasticity.
• Participate in 16 stimulation sessions over 8 weeks (2 sessions per week).
• Complete questionnaires about symptoms and daily function.
• Provide blood samples at four timepoints (Week 1, 4, 8, and 16).
• Inform the study team about any changes in health, medications, or adverse events during the study.
Testing and stimulation procedures will be adapted to each participant's mobility level, including people who walk with or without assistive devices and people who use wheelchairs.

Each participant will be in the study for about 16 weeks total:
• The stimulation intervention will be administered over an 8-week (2-month) period. Each stimulation session will last up to 1 hour, for a total of approximately 16 hours of stimulation across the study.
• 8 additional weeks without stimulation, ending with one follow-up assessment visit

Outline the schedule and timing of study procedures.
A. Consent and Screening Visit (Week -1)
On their first visit, the research team will review the consent form with participants, confirm eligibility for the study, and may conduct basic assessments related to walking or mobility (completed in the participant's usual manner, including the use of a mobility device or wheelchair, if applicable).
If participants prefer, the consent process may be completed remotely by emailing the IRB-approved consent form in advance. In such cases, participants will be given adequate time to review the document, and any questions will be addressed by the Principal Investigator or study personnel via phone, email, or Zoom, according to the participant's preference.

B. Stimulation Period and assessments (Weeks 1-8)
Before the study commences, clinicians and the study team will perform baseline assessments that may take up to 4 hours (only 4 sessions with the assessment throughout the study; Week 1, 4, 8, and 16), and may include:
1. Walking or mobility tests (completed in participants' usual way, including with a mobility device or wheelchair if applicable)
2. Muscle strength testing
3. Spasticity evaluations
4. Symptom questionnaires
5. Collect the first blood sample at baseline assessment

Participants will attend two visits per week for 16 total stimulation sessions. Each session lasts about 1.5 hours. During each session:
a. Electrodes will be placed on the skin over the lower back.
b. Participants will receive transcutaneous spinal cord stimulation (tSCS) at a comfortable, non-painful level. Participants may feel

tingling or tapping sensations under the electrodes.
c. Participants may be asked to perform simple movements, seated or standing, depending on their mobility abilities.
d. Provide blood samples on first visit for stimulation during week 1, mid-study at week 4, and last visit for stimulation at week 8.
e. At Week 4 and at the end of the 8-week stimulation period, participants will: repeat walking or mobility tests, strength and spasticity assessments, and complete questionnaires as described above during week 1.

C. Follow-Up Assessment Visit (Week 16)
About 8 weeks after the last stimulation session, participants will return for a final follow-up visit. Participants will repeat the same assessments, walking or mobility tests, strength testing, spasticity measures, questionnaires, and provide the fourth (last) blood draw. This helps researchers learn whether any changes continued after stimulation ended.

Procedures to be carried out with each participant

1. Transcutaneous Spinal Cord Stimulation (tSCS)
Transcutaneous Spinal Cord Stimulation (tSCS) will be delivered using a non-invasive spinal neuromodulation system (TESCoN or SCONE, SpineX Inc., Northridge, California, USA/ ARCEX (Onward Medical, USA). Two self-adhesive round electrodes placed at the midline over the lower thoracic (T11–T12) and/or upper lumbar vertebral levels (L1-L2) will serve as the cathodes, and two rectangular self-adhesive electrodes placed symmetrically over the iliac crests will serve as anodes. Electrode placement will remain the same regardless of the device used. Stimulation will be delivered as high-frequency pulsed current using frequencies within a predefined range (typically 1-90 Hz; with a carrier frequency to make stimulation more comfortable), and session parameters will be adjusted per participant and per visit. Current amplitude will begin at ~10 mA and be increased in small steps to a comfortable, tolerable level below strong muscle contraction or until responses plateau. Skin at electrode sites will be inspected, cleaned, and prepared to reduce impedance before each session. We will use a conducting gel to protect the skin during stimulation. A skin-temperature probe will be positioned adjacent to the cathode to monitor local temperature and reduce the risk of skin irritation or burning. All stimulation sessions will be conducted at the UK PM&R Research Lab, except the 4 sessions coupled with mobility assessments that will be conducted in the Human Performance Laboratory/Biodynamics lab at the UK Campus under the supervision of trained study personnel, with real-time monitoring for discomfort, autonomic symptoms, or adverse events. Participants may complete seated, standing, or mobility tasks during stimulation, depending on their functional level, including those who ambulate with or without assistive devices and those who use wheelchairs.

2. Motor and Mobility Assessments
Assessments will be adapted to participants' mobility level. Ambulatory participants will complete standard walking tests; wheelchair users will complete equivalent seated mobility tasks.
a) 10-Meter Walk Test (or equivalent wheelchair speed test): 10MWT assesses walking speed over a 10-meter walkway at a comfortable and maximum safe pace. Timing occurs between 2 and 8 meters to exclude acceleration/deceleration, with two trials averaged per speed. Assistive devices are allowed. The 10MWT is quick, reliable, and sensitive to gait changes in HSP. Although no formal MCID exists for HSP, small gait speed changes reflect meaningful motor function improvements.
b) 6-Minute Walk Test (or equivalent wheelchair endurance test): evaluates walking endurance by measuring the total distance walked over six minutes. Patients are instructed to walk as far as possible at their own pace, with rests allowed if needed. Distance measurements will be collected using an ActiGraph wearable activity monitor. The total distance reflects functional capacity and stamina, which are often affected in HSP due to progressive spasticity and weakness.
c) Sit-to-stand test: Participants will be asked to perform a sit-to-stand task, whereby they will be told to stand up and sit down onto a stool at their own self-selected pace.
d) Spastic Paraplegia Rating Scale (SPRS) clinician rating: SPRS is a reliable clinician-administered 13-item scale specific to HSP assessing gait, spasticity, strength, and sphincter function. Scores reflect disease severity and progression.
e) HSP-SNAP patient-reported symptom questionnaire: HSP-SNAP is a patient-reported questionnaire rating frequency and severity of HSP symptoms such as stiffness, weakness, imbalance, and pain.55 It captures subjective symptom burden and treatment impact.
f) 3D gait or movement analysis and surface EMG (when applicable): 3D Gait Analysis will be performed using a 14-camera motion capture system, wireless EMG system, and 3 ground force plates. We will track walking speed using timing gates. Wireless EMG sensors will be placed on the vastus lateralis, rectus femoris, and vastus medialis to assess muscle activation during the gait trials. The outcomes will include walking velocity, stance time, joint kinematics (hip, knee, and ankle) and joint kinetics.

All study participants will be asked to wear standardized sneakers during testing to reduce the impact of footwear on gait mechanics. Standardized sneakers will be provided.

All study participants will be asked to wear shorts and a tank top or sports bra for biomechanical testing. This attire will allow the research team to accurately visualize and palpate the anatomical landmarks needed to accurately track segment motion during biomechanical testing.

3. Strength and Spasticity Testing
a) Muscle Strength: Bilateral isometric knee strength will be obtained with three, 5-sec maximal voluntary isometric contractions (MVIC) separated by one-minute rest. EMG is recorded concurrently for normalization with gait data. Knee pain during contractions is recorded on an 11-point visual analog scale.
b) Modified Ashworth Scale (MAS): It's the standard clinical scale to rate muscle spasticity (tightness) in HSP. It measures muscle spasticity by assessing resistance to passive muscle stretch through the range of motion and grades the muscle tone on a 0-4 scale based on the resistance felt.
MAS 0-4 scale:
0: No increased muscle tone
1: Slight increase in tone; catch and release at the end of the range
1+: Slight increase; catch followed by minimal resistance through < half the range

2: More marked increase through most of the range, but the limb still moves easily
3: Considerable increase in tone; passive movement is difficult
4: Limb is rigid in flexion or extension

4. Lower Urinary Tract (LUT) and Bowel Function: will be quantified as an exploratory outcome using NBSS and NBDS questionnaires. NBSS assesses urinary frequency, urgency, incontinence, and catheter use, while NBDS examines bowel movement frequency, constipation, and incontinence.

o Lower Urinary Tract (LUT) Assessments
a) Neurogenic Bladder Symptom Score (NBSS): comprises 23 questions covering 3 domains, including incontinence, storage & voiding, and specific consequences, as well as one question on QoL.
b) The Incontinence-QoL (I-QoL): comprises 10 questions covering 3 domains, including avoidance and limiting behaviour, psychosocial impacts, and social embarrassment, which will be summarised as a total score. All scores, for each domain and a total, will be transformed into a continuous scale value.
o Bowel Dysfunction Assessments

a) Neurogenic bowel dysfunction score (NBD Score) The NBD score is a measure of both constipation and fecal incontinence and was developed for and validated in the SCI population This questionnaire comprises 10 questions focusing on defecation (i.e., frequency, duration, and clinical symptoms), constipation (i.e., use of aiding medication and digital stimulation), fecal incontinence (i.e., frequency, aiding medication, and flatus; and peri-anal skin problems. The consequential NBD score relates to four different neurogenic bowel dysfunction severity levels.
5. Blood Collection and Metabolic/protein profiling
Approximately 10 mL of venous blood samples will be collected at Weeks 1, 4, 8, and 16. Samples will be processed for metabolomic/lipid profiling and biomarker assays (including Big-tau). Targeted Metabolomic Profiling: Individuals with HSP also suffer from significant metabolic dysfunctions, notably in lipid metabolism. Many HSP genes encode proteins essential for lipid metabolism, with disturbances leading to impaired myelination, altered membrane trafficking, and axonal degeneration. Fasting plasma samples collected during the visits 2, 9, 17 and 18 will be analyzed for ~1100 lipid and polar metabolites across pathways including glycolysis, the tricarboxylic acid cycle, amino acid metabolism, and nucleotide biosynthesis [at our CNS Metabolism Center of Biomedical Research Excellence (COBRE) Metabolomics Core]. One plasma aliquot will be shipped to Drexel University for identification of Big-tau as a HSP biomarker (Dr. Emanuela Piermarini). Some analyses may involve genetic-related testing on de-identified samples. Individual research results will not be returned unless an important incidental finding is confirmed and IRB-approved.
6. Statistical Analysis Plan
Descriptive statistics will summarize feasibility (recruitment rate, retention, session adherence) and safety (adverse events). Efficacy outcomes will be analyzed using within-subject comparisons across timepoints (Week 1 vs Week 4 vs Week 8 vs Week 16). Depending on distribution, repeated-measures ANOVA or non-parametric Friedman tests will be used. Effect sizes (Cohen's d or Kendall's W) with 95% CIs will be reported to inform future powered trials. Missing data will be handled using mixed models or last-observation-carried-forward for sensitivity analyses. For Aim 1a, immediate effects of a single tSCS session will be assessed using paired t-tests. For Aim 1b, longitudinal changes across baseline, 8 weeks, and 16 weeks will be evaluated using linear mixed effects models to account for within-subject correlations and missing data. Primary and secondary outcomes (e.g., 10MWT, SPRS, MAS) will be treated as continuous variables. MAS, while ordinal, will be analyzed as continuous in the primary model with sensitivity analyses using nonparametric or ordinal methods. Post hoc comparisons will adjust for multiple testing. For Aim 2, which is exploratory and hypothesis-generating, univariate logistic regression will identify candidate predictors of motor response (defined as =0.1 m/s improvement in 10MWT or =1-point reduction in MAS). Candidate baseline predictors will include demographic and clinical variables such as age, sex, disease duration, genetic subtype, clinical phenotype (pure vs. complex), baseline gait speed, baseline MAS, lower-limb strength, and mobility status/assistive device use. Predictor-response associations will first be screened with univariate logistic regression. A multivariable logistic regression model will then be built using either backward stepwise selection guided by Akaike Information Criterion (AIC) or LASSO regularization, depending on sample size and collinearity. Model performance will be summarized by discrimination (AUROC) and calibration (Hosmer–Lemeshow test and calibration plots). Internal validation will be performed using 5-fold cross-validation and bootstrap resampling to assess stability and optimism. Finally, coefficients from the final model will be translated into a preliminary clinical scoring tool to stratify likelihood of response, acknowledging that external validation in a larger cohort will be required. Analyses will be conducted with a = 0.05, emphasizing estimation over significance testing.

This study does not involve deception. Participants will be fully informed about the purpose, procedures, risks, and voluntary nature of the study during the consent process. Therefore, no deception-related debriefing is required.

Standard/Routine Clinical Care (not part of the study)
Participants will continue their usual clinical care for HSP throughout participation. Routine care may include, but is not limited to:
• Ongoing neurology/rehabilitation follow-up visits
• Physical therapy or occupational therapy prescribed as part of clinical care
• Standard medications for spasticity or symptoms (see medication rules below)
• Use of assistive devices such as braces, canes, walkers, or wheelchairs
• Other clinically indicated evaluations or treatments determined by their treating physicians
These routine care procedures are not required by the research study and will occur whether or not the individual participates.

Research-Specific Procedures (performed only for this project)
All of the following are conducted specifically for research and are not standard care for HSP:
1. tSCS sessions (16 sessions over 8 weeks).
2. Research assessments in Weeks 1, 4, 8, and 16, including:
• walking tests or equivalent wheelchair mobility tests
• endurance testing (6-minute walk or equivalent)

• lower-limb strength testing
• spasticity evaluation (Modified Ashworth Scale)
• clinician rating (Spastic Paraplegia Rating Scale; SPRS)
• participant symptom questionnaires (HSP-SNAP)
• questionnaires to assess bladder and bowel function
• optional 3D gait/movement and surface EMG testing, when applicable
3. Research blood draws (4 total) at Weeks 1, 4, 8, and 16 for metabolomics/biomarkers/genetic-related analyses on de-identified samples.
4. Additional monitoring for adverse events related to stimulation and study testing.

Medication Rules During Participation
Permitted Medications
Participants may continue stable standard-of-care medications, including:
• antispasticity agents such as baclofen, tizanidine, dantrolene, diazepam/clonazepam
• botulinum toxin injections, if already part of routine care
• medications for bladder symptoms, pain, sleep, mood, or other chronic conditions
• vitamins/supplements, if stable
Requirement: All neurologic/spasticity-related medications must be stable for at least 4 weeks before enrollment and participants are asked to avoid dose changes during the 8-week stimulation period, unless medically necessary.

Forbidden / Restricted Medications or Interventions
Participants will be excluded or paused if they initiate or substantially change treatments that could confound outcomes or increase risk, including:
• starting a new antispasticity medication or making major dose changes during the 8-week stimulation phase (unless required for safety/medical care)
• starting or changing neuromodulation therapies, including any implanted or external electrical stimulation targeting the spine or lower limbs
• enrolling in another interventional clinical trial that could affect mobility/spasticity during participation
• new implantation of electrical medical devices (e.g., pacemakers, intrathecal pumps) during the study
If a medically necessary medication change occurs, the research team will document it and determine whether the participant can safely continue, pause, or withdraw, depending on timing and magnitude of the change.

Visit-by-visit listing all procedures that will take place: See attached file

Attachments

Attach Type	File Name
ResearchProcedures	Highlighted Visit by Visit procedures_SCS-HSP_Sachdeva_rev-1.pdf
ResearchProcedures	Clean_Visit by Visit procedures_SCS-HSP_Sachdeva_rev-1.pdf

Data Collection & Research Materials

In this section, please provide the following:

- Describe all sources or methods for obtaining research materials about or from living individuals (such as specimens, records, surveys, interviews, participant observation, etc.), and explain why this information is needed to conduct the study.
- For each source or method described, please list or attach all data to be collected (such as genetic information, interview scripts, survey tools, data collection forms for existing data, etc.).
- If you will conduct a record or chart review, list the beginning and end dates of the records you will view.

1. In-person clinical and functional assessments
• Method: Participants will complete standardized physical and mobility/functional tests at the Human Performance Laboratory/Biodynamics Lab at baseline (Week 1), mid-intervention (week 4), post-intervention (week 8), and follow-up (week 16), with brief safety checks at each stimulation visit.
• Rationale: These measures quantify motor function, mobility, strength, and spasticity to evaluate feasibility, safety, and preliminary efficacy of tSCS in HSP across diverse mobility levels (ambulatory and wheelchair users).

2. Participant-reported questionnaires
• Method: Surveys administered in Weeks 1, 4, 8, and 16.
• Rationale: Captures symptoms and functional impact of HSP from the participant's perspective, complementing clinician-rated and performance outcomes.

3. Biomechanical / neurophysiological recordings (as applicable to mobility level)
• Method: 3D gait/movement analysis and surface EMG collected during mobility tasks at assessment visits.
• Rationale: Provides objective mechanistic/physiologic markers of motor control and changes in neuromuscular activation patterns after tSCS. For wheelchair users, equivalent movement tasks will be used.

4. Venous blood specimens
• Method: Blood draws (~10mL) at Weeks 1, 4, 8, and 16; samples processed and de-identified for laboratory analyses.
• Rationale: Enables metabolomic/lipid profiling and biomarker assays (including Big-tau and related markers) to explore biological correlates of tSCS response and disease state.

All data to be collected:
1. In-person assessments
• Demographics collected at baseline: age, sex, race/ethnicity, disease duration, genetic subtype (if known), phenotype (pure vs. complex), assistive device use/mobility status.
• Mobility/function outcomes:
o 10-Meter Walk Test or wheelchair speed equivalent
o 6-Minute Walk Test or wheelchair endurance equivalent
o Sit-to-Stand test
• Strength measures:
o Lower-limb dynamometry or standardized manual muscle testing
• Spasticity measures:
o Modified Ashworth Scale (MAS)
o Clonus (if present)
• Clinician-rated disease severity:
o Spastic Paraplegia Rating Scale (SPRS)
• Session-level safety/comfort logs:
o adverse events, skin checks, discomfort ratings, stimulation tolerability.
2. Questionnaires
• HSP-SNAP (symptom frequency/severity)
• • NBSS and NBDS assessments for lower urinary tract and bowel function assessment
• Falls history and mobility-confidence items if included in CRFs.
3. Biomechanics / EMG
• 3D kinematics/kinetics (joint angles, spatiotemporal gait parameters) for ambulatory participants
• Equivalent seated mobility/movement kinematics for wheelchair users as applicable
• Surface EMG from lower-limb muscles during tasks (timing, amplitude, co-contraction indices).
4. Blood specimens
• Metabolomic and lipid profiling outputs (de-identified)
• Protein/biomarker assays including Big-tau (de-identified)
• Any genetically related research analyses performed on de-identified samples.

Attachments

Resources

Describe the availability of the resources and adequacy of the facilities that you will use to perform the research. Such resources may include:

- Staffing and personnel, in terms of availability, number, expertise, and experience;
- Computer or other technological resources, mobile or otherwise, required or created during the conduct of the research;
- Psychological, social, or medical services, including equipment needed to protect subjects, medical monitoring, ancillary care, or counseling or social support services that may be required because of research participation;
- Resources for communication with subjects, such as language translation/interpretation services.

The study team includes investigators and staff with complementary clinical, neuromodulation, biomechanics, metabolomics, and data-science expertise, all of whom are available within UK-PM&R research lab and the Human Performance Laboratory/Biodynamics lab at the UK Campus to support recruitment, intervention delivery, assessments, and analysis. The BioDynamics laboratories at the University of Kentucky will be utilized for the gait analyses and strength testing performed in this study.

Staffing and Personnel:
Dr. Rahul Sachdeva (PI) – Assistant Professor, PM&R. Brings ~15 years of experience in neuromodulation and tSCS across neurological populations and will oversee all study activities and safety.
Dr. Michael Abeeb Samaan (Co-Investigator) – Associate Professor and Co-Director of the Human Biodynamics Lab. Provides expertise in 3D gait analysis, EMG, and strength assessment.
Dr. Sara Salles (Co-Investigator) – Professor/Clinician, PM&R. Supports recruitment from the established HSP clinical cohort and performs clinical scales (SPRS, MAS).
Dr. Samirkumar Patel (Co-Investigator) – Assistant Professor and Co-Director of the CNS Metabolism COBRE Metabolomics Core. Leads metabolomics/biomarker workflows.
Dr. Vivek Pandey (Co-Investigator) – Scientist II with ~11 years of experience in molecular biology, neurodegeneration, and data handling, and certified phlebotomist; will manage daily operations, including recruitment, tSCS delivery, biospecimen handling, and data analysis.
Ms. Makyra Ross (Study personnel) – Graduate student with research experience in hereditary spastic paraplegia (HSP) studies conducted in Dr. Sachdeva's laboratory. Assists with daily operations including recruitment, tSCS delivery, biospecimen handling, and data analysis.
Dr. Zakari Dymock (Study personnel) – SCI Physician and Assistant Professor, PM&R. Contributes to recruitment and administers validated clinical scales, including the SPRS and MAS.
Dr. Susan McDowell (Study personnel) – Janet Galloway Carter Endowed Chairperson, Associate Professor, Physical Medicine and Rehabilitation, oversees recruitment of participants, clinical procedures and data analysis.
Ms. Hannah Whitaker (Study personnel) – Clinical Coordinator and certified phlebotomist, will assist with biomechanical data collection and blood sampling/processing.

Ms. Holly Elliott (Study personnel) – Research Engineer, will assist with data collection and processing of motion capture and muscle function testing data.
Biostatistics support – A designated biostatistical consultant (Dr. Rich Cohn) will support the analysis of anonymized data.

Primary site: Dr. Sachdeva's clinical research lab within the PM&R Department at Cardinal Hill Rehabilitation Hospital (CHRH) and Dr. Samaan's Human Performance Laboratory/Biodynamics Lab at the UK campus.
Space and access: The lab is a 1,400-sq-ft dedicated clinical research space, located near the main entrance for easy access for participants using assistive devices or wheelchairs.
Capabilities: The lab is equipped for comprehensive motor, autonomic, and metabolic assessments in individuals with HSP.
• Computer or other technological resources, mobile or otherwise, required or created during the conduct of the research;
• tSCS equipment: Noninvasive neuromodulation delivered with the SCONE system (SpineX Inc.)/ARCEX (Onward Medical), with device support and troubleshooting available through the manufacturer.
• Biomechanics/EMG: Dr. Samaan's Lab provides advanced 3D motion capture, wireless EMG, and force-plate systems to support all proposed biomechanical outcomes.
• Data systems: Research data will be captured on secure, password-protected UK servers/databases with coded identifiers and role-based access, consistent with institutional policy and HIPAA protections (details in confidentiality section).
• Clinical oversight: Stimulation and assessment visits occur under the supervision of principal investigator, Dr. Rahul Sachdeva, trained research personnel, and medical supervisor, with safety screening at each visit.
• Skin and stimulation safety: The team has established procedures such as using gel for safe parameter titration, temperature monitoring, and real-time intervention if discomfort or skin heating occurs.
• Participant contact: The team has established phone/email/secure messaging systems for scheduling, reminders, and follow-ups.
• Accessibility: CHRH/UK facilities are wheelchair-accessible and appropriate for participants with varying mobility limitations.
• Language services (as needed): UK HealthCare interpretation/translation services are available for participants who require communication support in languages other than English (standard institutional resource).

Potential Risks & Benefits

Risks

- Describe any potential risks – including physical, psychological, social, legal, ability to re-identify subjects, or other risks. Assess the seriousness and likelihood of each risk.
- Which risks may affect a subject's willingness to participate in the study?
- Describe likely adverse effects of drugs, biologics, devices or procedures participants may encounter while in the study.
- *Qualitative research* - describe ethical issues that could arise while conducting research in the field and strategies you may use to handle those situations.
- Describe any steps to mitigate these risks.

Benefits

- Describe potential direct benefits to study participants – including diagnostic or therapeutic, physical, psychological or emotional, learning benefits. This cannot include incentives or payments.
- State if there are no direct benefits.
- Describe potential benefits to society and/or general knowledge to be gained.

Describe why potential benefits are reasonable in relation to potential risks. If applicable, justify why risks to vulnerable subjects are reasonable to potential benefits.

Risks:
This study involves noninvasive tSCS, mobility and strength assessments, and collection of venous blood samples. Overall risk is expected to be low to moderate, but side effects and unexpected events may occur. Participants will be monitored at every visit, and procedures will be adjusted or stopped if safety concerns arise.
1. Risks related to tSCS (electrical stimulation through surface electrodes)
tSCS is noninvasive and has been well tolerated in other neurological populations, but may cause the following side effects:
a) Common/expected risks
a. Tingling, buzzing, or tapping sensations during stimulation
b. Mild skin redness or warmth under electrodes
c. Temporary discomfort that resolves when stimulation is adjusted or stopped
b) Occasional risks
a. Transient muscle twitching, cramping, or fatigue during or after sessions
b. Short-term increase in spasticity or soreness following stimulation or associated movement activities
c) Rare but important risks
a. Skin irritation, blistering, or superficial burns beneath electrodes
b. Allergic reaction to adhesive or gel
c. Prolonged pain or spasticity flare lasting longer than 24 hours
Risk mitigation: To reduce skin-related risks, we will use a special gel cushion to protect participants' skin during the stimulation. Moreover, stimulation is delivered using a delayed biphasic waveform to minimize net charge and heating. Electrode sites will be inspected before and after each session, and stimulation intensity will be maintained at a comfortable sub-motor threshold. Participants will be instructed to report burning, sharp pain, dizziness, or any unusual sensation immediately so that stimulation can be paused, modified, or stopped. If a skin condition persists, they will be referred to one of the clinicians listed on the protocol.
2. Risks related to mobility, gait, and strength testing

Testing may include walking tasks for ambulatory participants or equivalent seated mobility tasks for wheelchair users. There are no known side effects from being filmed by high-speed cameras during gait analysis.
a) Common/expected risks
a. Fatigue during or after testing
b. Short-term muscle soreness
b) Occasional risks
a. Lightheadedness
b. Increased stiffness or spasticity
c. Muscle cramping
c) Rare but important risks
a. Loss of balance during ambulatory tasks
b. Falls, which could cause minor or serious injury (e.g., sprain, fracture)
c. Minor strains or sprains during testing
Risk mitigation: All testing will be supervised by trained study staff. Participants may use their usual mobility device (wheelchair, walker, cane, braces). Rest breaks will be offered as needed. For ambulatory testing, staff will guard participants closely and may use gait belts or other supports to reduce fall risk.
3. Risks related to venipuncture (blood draws)
Venous blood draws will be performed four times (Weeks 1, 4, 8, and 16). Risks associated with blood draws are generally slight, but may include:
a) Common/expected risks
a. Brief pain at the needle site
b. Bruising or soreness
b) Occasional risks
a. Bleeding
b. Light-headedness or fainting
c) Rare risks
a. Infection at the needle site
Risk mitigation: Blood drawings will be performed by trained personnel using sterile techniques. Participants who feel faint will be assisted in a seated or supine position and monitored until symptoms resolve.
4. Risks related to pregnancy
The effects of tSCS on an embryo or fetus are unknown. Pregnant individuals are excluded from participation. If a participant becomes pregnant during the study, stimulation will stop immediately and the participant will be withdrawn from the intervention for safety reasons. There may be risks to an embryo or fetus that are currently unforeseeable.
5. Risks to privacy/confidentiality (including genetic/biomarker testing)
There is a small risk of breach of confidentiality, including genetic or biomarker data derived from blood samples. Data will be coded, identifiers stored separately, and access restricted to authorized personnel.
6. Unknown risks
There is always a chance that any medical treatment can harm. The research treatments/procedures in this study are no different. In addition to risks described above, participants may experience previously unknown risks or side effects.

Benefits:
We do not know if the participants will receive personal benefit from taking part in this study. However, some people who have received tSCS in other neurological conditions have experienced improvements in movement, walking ability, coordination, and reductions in muscle stiffness during or after repeated stimulation sessions. We do not know whether people with HSP will experience similar benefits. Even if they do not personally benefit, information learned from this study may help improve future treatment options for people with HSP.
Possible direct benefits may include:
1. improved lower-limb motor control and functional mobility (or wheelchair-based mobility efficiency),
2. reduced muscle stiffness/spasticity,
3. improved walking speed/endurance for ambulatory participants,
4. better ease of movement for participants who use wheelchairs, and
5. potential secondary improvements in autonomic functions and/or quality of life.
These potential benefits are not guaranteed and may vary across participants.

Potential benefits to society and general knowledge:
This study will generate the first clinical evidence on the feasibility, safety, and preliminary efficacy of noninvasive tSCS in HSP, addressing a major unmet need because current treatments are only modestly effective and largely symptomatic.
Results will:
• inform larger controlled trials and clinical trial readiness in HSP,
• advance understanding of how spinal neuromodulation modulates HSP motor circuitry, and
• support development of predictor-based, precision stimulation approaches to identify who is most likely to respond.
Potential benefits are reasonable relative to risks because:
1. High unmet clinical need: HSP causes progressive spasticity and disability, and available treatments offer only limited, temporary relief without altering disease trajectory.
2. Strong scientific rationale and preliminary evidence: Spinal cord neuromodulation has demonstrated promising effects in HSP (via invasive eSCS case reports) and tSCS has repeatedly improved motor and autonomic outcomes in other neurological populations, supporting a likelihood of benefit with a noninvasive approach.
3. Low to moderate, well-characterized risks: tSCS is noninvasive and has a favorable safety profile. Anticipated risks (e.g., transient discomfort, skin irritation, fatigue, and standard blood-draw risks) are monitored each session, and electrode-site heating risk is mitigated by waveform choice and real-time temperature monitoring.

4. No special vulnerable population risks beyond mobility limitation: Participants may have diverse mobility abilities, including wheelchair users, but the procedures are adaptable (seated or assisted tasks) and do not present disproportionate risk to those with greater disability. The potential to improve meaningful function in this underserved group justifies inclusion.

Available Alternative Opportunities/Treatments

Describe alternative treatments or opportunities that might be available to those who choose not to participate in the study, and which offer the subject equal or greater advantages. If applicable, this should include a discussion of the current standard of care treatment(s).

Standard care may include physical therapy, stretching or exercise programs, assistive devices, and medications prescribed to help manage spasticity or mobility difficulties. Participants and their doctors may also discuss other treatment options that are available outside of research. If an individual choose not to participate, they will still receive all the usual care for their condition, and nothing about their medical care will be affected.

Back to Top

Records, Privacy, and Confidentiality

Specify where the data and/or specimens will be stored and how the researcher will ensure the privacy and confidentiality of both. Specify who will have access to the data/specimens and why they need access.

Describe how data will be managed after the study is complete:

- If data/specimens will be maintained, specify whether identifiers will be removed from the maintained information/material.
- If identifiers will not be removed, provide justification for retaining them and describe how you will protect confidentiality.
- If the data/specimens will be destroyed, verify that this will not violate retention policies and will adhere to applicable facility requirements.

If this study will use de-identified data from another source, describe what measures will be taken to ensure that subject identifiers are not given to the investigator.

If applicable, describe procedures for sharing data/specimens with collaborators not affiliated with UK.

For additional considerations:
Return of Research Results or Incidental Research Findings
HIPAA policies
FERPA policies
Procedures for Transfer agreements
Information regarding multi-site studies
NIH Genomic Data Sharing (GDS) Policy
Digital Data

The new data security compliance requirements under 28 CFR Part 202 are in effect as of 10/6/25. For any questions please contact Sara Poll, UK's Research Security Administrator, researchsecurity@uky.edu

Research data (electronic):
All identifiable research data (e.g., consent forms, screening logs, contact information) and coded study data (assessments, stimulation logs, questionnaires, EMG/gait files, lab results) will be stored on secure, password-protected University of Kentucky servers and/or UK-approved encrypted databases. Data will be labeled with a unique study ID; direct identifiers (name, MRN, DOB, contact info) will be stored separately from research datasets. Only authorized study personnel will have access, and access will be role-based.

Some study sessions and procedures may be videotaped and/or photographed for research and documentation purposes. When applicable, facial features and other identifying information will be blurred or otherwise de-identified to protect participant privacy, and all recordings will be stored securely with access limited to authorized study personnel.

Paper records:
Any paper documents (e.g., signed consent forms, paper questionnaires) will be kept in locked filing cabinets within Dr. Sachdeva's PM&R research lab, a secure UK-affiliated facility.

Biological specimens:
Blood samples will be processed and stored as coded specimens labeled only with a study ID. Samples will be stored in secure, access-controlled freezers in Dr. Rahul Sachdeva's lab, located at the Biomedical Biological Sciences Research Building (BBSRB), Dr. Samaan's freezer located in room 218 in the Seaton Center, and/or the CNS Metabolism COBRE Metabolomics Core as required for metabolomic assays. A master key linking study IDs to participant identities will be stored separately on a secure UK server accessible only to the PI and designated staff.
Confidentiality protections:
• Study IDs will be used on all data/specimens.
• The linkage key will be stored separately and securely.
• Electronic data will be encrypted in transit and at rest where supported.

• Only minimum necessary PHI will be accessed for eligibility/safety.
• Results will be reported in aggregate; no individual will be identifiable in publications or presentations.
Access will be limited to personnel who need it to conduct the study:
• PI and Co-Investigators (UK-affiliated): access to coded research data for oversight, safety, and analysis.
• Research staff/trainees listed on IRB: access to coded data for scheduling, stimulation delivery, assessments, and data entry.
• Metabolomics Core staff (UK-affiliated): access to coded plasma/serum samples only, to run approved assays.
• Biostatistical consultant: access to de-identified or coded datasets for analysis.
No one outside the approved study team will have access to identifiers.

Data/specimen management after study completion
Retention: Study data and coded specimens will be retained per UK and sponsor/ORI retention policies (typically at least 6 years after study closeout), unless a longer period is required for regulatory reasons.
De-identification for future use/sharing: After primary analyses are complete, datasets intended for sharing will have all direct identifiers removed and will be released only in de-identified form through public repositories, consistent with proposal's open-science plan.
Specimens: Remaining coded specimens may be retained for future HSP-related biomarker work. Any future use will involve only coded/de-identified samples and will require IRB approval consistent with the consent's future-use language. If specimens are exhausted or no longer needed, they will be destroyed using UK-approved biohazard disposal procedures in compliance with institutional policy.

Sharing data/specimens with non-UK collaborators
Consistent with the proposal, the study will share:
1. De-identified study data through appropriate public repositories after completion of primary analyses.
2. Anonymized/coded plasma aliquots with Drexel University for Big-tau biomarker discovery.

☑ **UK IRB policies state that IRB-related research records must be retained for a minimum of 6 years after study closure. Check this item to confirm that you will retain all IRB-related records for a minimum of 6 years after study closure.**

Payment

Describe the incentives (monetary or other) being offered to subjects for their participation. If monetary compensation is offered, indicate the amount and describe the terms and schedule of payment. Please review this guidance for more information on payments to subjects, including restrictions and expectations.

Participants may receive up to $725 to take part in this study. Participants will receive $100 for completing assessments conducted during the four study visits and $25 for each additional stimulation visit, as well as for the consent visit if the participant chooses to complete consent in person. If a participant withdraws early, compensation will be prorated, such that they will be paid for the study visits or portions of the study they have completed.

Participants will receive compensation in the form of a check/gift card. Payment will be provided upon completion of the study, or on a prorated basis if a participant withdraws early, and is expected to be distributed within 6-8 weeks of the study completion/withdrawal.

If participants choose to withdraw early, their payment will be prorated, meaning they will be paid for the visits or parts of the study that they completed.
With a few exceptions, study payments are considered taxable income reportable to the Internal Revenue Service (IRS). A form 1099 will be sent to them if their total payments for research participation are $600 or more in a calendar year.

Costs to Subjects

Include a list of services and/or tests that will not be paid for by the sponsor and/or the study (e.g., MRI, HIV). Keep in mind that a subject will not know what is "standard" – and thus not covered by the sponsor/study – unless you tell them.

Participants and/or their insurance company, Medicare, or Medicaid will be responsible for the costs of all care and treatment that participants would normally receive for any conditions they may have. These are costs that are considered medically necessary and will be part of the care they receive, even if they do not take part in this study.
The University of Kentucky may not be allowed to bill participants' insurance company, Medicare, or Medicaid for the medical procedures done strictly for research. The research procedures in this study include the spinal cord stimulation sessions (tSCS), mobility and strength assessments, research questionnaires, and laboratory testing done only for research. Therefore, this research will be paid by the University of Kentucky and will not be billed to the participant or their insurance.
If the participant receives routine medical care during the study that is not part of the research, those costs may be billed to the participant or their insurance. Participants' insurers, Medicare, or Medicaid, may agree to pay for the costs. However, a co-payment or deductible may be needed from the participant. The amount of this co-payment or deductible may be costly.

Data and Safety Monitoring

The IRB requires review and approval of data and safety monitoring plans for greater than minimal risk research or NIH-funded/FDA-regulated clinical investigations.

- If you are conducting greater than minimal risk research, or your clinical investigation is NIH-funded, describe your Data and Safety Monitoring Plan (DSMP). Click here for additional guidance on developing a Data and Safety Monitoring Plan.

- If this is a non-sponsored investigator-initiated protocol considered greater than minimal risk research, and if you are planning on using a Data and Safety Monitoring Board (DSMB) as part of your DSMP, click here for additional guidance for information to include with your IRB application.

This investigator-initiated clinical study is considered greater than minimal risk due to the repeated delivery of noninvasive spinal cord stimulation, mobility testing with fall risk, and serial blood draws. Safety oversight will be conducted by the PI, Dr. Rahul Sachdeva and co-investigators, with additional independent monitoring as described below.

Safety Objectives
The DSMP is designed to:
1. Detect, document, and manage adverse events (AEs) and serious adverse events (SAEs) promptly.
2. Ensure stimulation parameters remain safe and tolerable across participants with diverse mobility abilities, including wheelchair users.
3. Identify safety trends early and implement corrective actions.
4. Ensure timely reporting to the IRB and sponsor.
1. Safety Monitoring Procedures
1.1 Visit-Level Safety Monitoring
Safety will be monitored on every study visit:
Before each stimulation session (Weeks 1-8):
• Review of new symptoms, falls, hospitalizations, medication changes, or health events since prior visit.
• Confirmation that no new exclusion criteria have emerged (e.g., implanted device, pregnancy).
• Skin inspection at planned electrode sites.
During stimulation:
• Continuous observation for discomfort, autonomic symptoms (e.g., dizziness, nausea, sweating), abnormal muscle responses, or participant distress.
• Real-time adjustment of stimulation intensity if needed.
• Application of gel at the stimulation site
• Skin temperature monitoring adjacent to the cathode (to reduce burn risk).
After stimulation:
• Repeat skin inspection and participant symptom check (pain, fatigue, spasticity changes, unusual sensations).
• Documentation of any AE.
At major assessment visits (Weeks 1, 4, 8, and 16):
• Record vital signs as indicated by protocol.
• Review interval medical changes and capture any AEs/SAEs.
1.2 Mobility / Fall Risk Safety
• Ambulatory tests will be supervised by trained staff using gait belts/spotting as needed.
• Participants may use their usual devices (walker, cane, braces) or wheelchair.
• Rest breaks will be provided as needed.
• Any fall or near-fall will be documented as an AE.
1.3 Blood Draw Safety
• Blood draws will be performed by trained personnel using sterile technique.
• Standard monitoring for bruising, fainting, bleeding, or infection.
• Any complication will be recorded as an AE.

2. Adverse Event Definitions and Documentation
2.1 Definitions
• Adverse Event (AE): Any unfavorable or unintended sign, symptom, or medical occurrence temporally associated with participation, whether or not related to the study.
• Serious Adverse Event (SAE): Any AE that results in death, is life-threatening, requires hospitalization or prolongs hospitalization, causes persistent/significant disability, or requires intervention to prevent these outcomes.
2.2 Expected AEs
Expected risks include mild skin redness/irritation, tingling or discomfort during stimulation, transient muscle fatigue or increased spasticity, dizziness during testing, fall risk during ambulatory tasks, and routine venipuncture risks (pain, bruising).
2.3 Recording and Grading
• All AEs and SAEs will be recorded in case report forms at each visit.
• Events will be graded by severity (mild, moderate, severe) and relationship to study (definitely, probably, possibly, unlikely, unrelated).
• Date of onset, duration, management, and resolution will be documented.

3. Stopping Rules and Safety-Based Withdrawal
3.1 Immediate Session-Level Stopping Rules
Stimulation will be reduced or stopped immediately if:
• Participants report intolerable discomfort, burning, sharp pain, or distress.
• Persistent abnormal autonomic symptoms occur (e.g., sustained dizziness, nausea).
• Skin temperature elevation or visible skin injury is detected.
• Staff determine continuation is unsafe.
3.2 Participant Withdrawal Criteria
Participants will be withdrawn from stimulation (and possibly the study) if any of the following occur:
• Superficial burn or clinically significant skin injury at electrode sites.
• New implanted electronic devices or medical diagnosis increasing stimulation risk.

• Pregnancy.
• SAE judged related or possibly related to tSCS or testing.
• PI/medical supervisor determines risk outweighs benefit.

4. Independent Safety Review (Monitoring Mechanism)
As per the UK-IRB guidelines, the pilot nature and small sample size, it appears a full DSMB is not required, However, a DSMP is in place as mentioned above. Moreover, if required, independent oversight will be implemented through a Medical Monitor / Safety Reviewer who is not involved in day-to-day conduct of the study (e.g., a PM&R or Neurology faculty member).
Medical Monitor responsibilities:
• Review all AEs/SAEs quarterly or after every 5 enrolled participants (whichever comes first).
• Provide written recommendations on whether study should continue unchanged, be modified, paused, or terminated for safety.
• Be available for ad hoc review of any SAE.
5. Reporting Plan
• SAEs and unanticipated problems will be reported to the UK IRB/ORI per institutional policy, typically within 24-48 hours of investigator awareness.
• Non-serious AEs will be summarized at continuing review and reported sooner if they indicate increased risk.

6. Data Quality and Integrity Monitoring
• Data will be entered into secure UK servers/databases using study IDs.
• Double-checks or automated validation rules will be used for key outcomes (mobility, MAS, SPRS, blood sample labels).
• Any protocol deviations will be logged, reviewed by the PI, and reported to IRB/sponsor when required.

7. Risk-Benefit Reassessment
Dr. Rahul Sachdeva, PI, and the study team will periodically review:
• AE frequency/severity trends
• participant tolerability to stimulation
• fall or mobility safety incidents
• any indication that risk profile exceeds what was anticipated

If risk increases beyond acceptable levels, enrollment will pause and the IRB and sponsor will be notified with a corrective action plan.

Back to Top

Future Use and Sharing of Material (e.g., Data/Specimens/Information)

If the material collected for this study will be used by members of the research team or shared with other researchers for future studies, please address the following:

- list the biological specimens and/or information that will be kept
- briefly describe the types, categories and/or purposes of the future research
- describe any risks of the additional use
- describe privacy/confidentiality protections that will be put into place
- describe the period of time specimens/information may be used
- describe procedures for sharing specimens/information with secondary researchers
- describe the process for, and limitations to, withdrawal of specimens/data

The study team will retain the following coded (study-ID labeled) materials:
• Blood-derived specimens: coded plasma/serum aliquots remaining after primary metabolomic/biomarker analyses.
• De-identified/coded research data: motor and mobility outcomes (10MWT or wheelchair equivalent, 6MWT or equivalent, Sit-to-Stand test), spasticity measures (MAS), clinician ratings (SPRS), questionnaires (HSP-SNAP), NBSS and NBDS questionnaires, stimulation parameters/tolerability logs, and mechanistic datasets (3D gait/motion capture and surface EMG where applicable).

Direct identifiers (name, MRN, DOB, contact info) will not be kept with these research materials.

Stored coded specimens and de-identified data may be used for future studies aimed at:
• Understanding biological mechanisms and biomarkers of HSP, including metabolomic, lipidomic, and protein biomarker work (e.g., Big-tau and related markers).
• Exploring predictors of response to neuromodulation and refining personalized tSCS protocols for HSP.
• Secondary analysis linking biological markers with motor, spasticity, and functional outcomes.
• Sharing de-identified datasets to support replication, meta-analyses, and development of larger controlled trials in HSP.
Future use will be limited to HSP/neuromotor/rehabilitation-relevant questions under IRB oversight.

Risks of additional use
Risks of future use are primarily related to privacy:
• Although identifiers will be removed, there is a small risk of loss of confidentiality or re-identification, especially for rare conditions.
• If genetic or biomarker analyses are performed, results could theoretically reveal sensitive health information; however, no individual results will be returned for clinical use.
No additional physical risk is expected because future research uses already-collected materials.

To protect privacy:

• All retained materials will be labeled only with a study ID.
• The code key linking IDs to identities will be stored separately on secure UK servers accessible only to the PI/designated staff.
• Data and specimens will be stored in password-protected databases/locked freezers in UK-approved research space.
• Any shared data/specimens will be de-identified or coded, and recipients will be prohibited from attempting re-identification.

Coded specimens and de-identified datasets may be stored and used for an indefinite period, consistent with the goal of enabling future HSP-related research and open-science data sharing.

Procedures for sharing with secondary researchers
• De-identified datasets may be shared through public repositories or with qualified investigators after IRB approval, consistent with open-science plan.
• Coded plasma aliquots may be shared with external collaborators only under an approved Material Transfer Agreement (MTA) and IRB authorization. One planned transfer is to Drexel University for Big-tau biomarker discovery using anonymized plasma.
• Secondary researchers must agree to use materials only for approved aims, maintain secure storage, and not share onward without permission.

Withdrawal process and limitations
Participants may request withdrawal of stored specimens/data at any time by contacting the Dr. Rahul Sachdeva, PI, in writing.
• If materials are still coded and identifiable via the key, they will be destroyed and not used for future studies.
• If materials/data have already been de-identified and shared or placed in a repository, withdrawal may not be possible because the research team can no longer link them to the participant.

This limitation will be clearly stated in the consent.

Are you recruiting or expect to enroll **Non-English Speaking Subjects or Subjects from a Foreign Culture**? (does not include short form use for incidentally encountered non-English subjects)
○ Yes ◉ No

Non-English Speaking Subjects or Subjects from a Foreign Culture

Recruitment and Consent:

Describe how information about the study will be communicated to potential subjects appropriate for their culture, and if necessary, how new information about the research may be relayed to subjects during the study.
When recruiting Non-English-speaking subjects, provide a consent document in the subject’s primary language. After saving this section, attach both the English and translated consent documents in the “Informed Consent" section.

Cultural and Language Consultants:

The PI is required to identify someone who is willing to serve as the cultural consultant to the IRB.

- This person should be familiar with the culture of the subject population and/or be able to verify that translated documents are the equivalent of the English version of documents submitted.
- The consultant should not be involved with the study or have any interest in its IRB approval.
- Please include the name, address, telephone number, and email of the person who agrees to be the cultural consultant for your study.
- ORI staff will facilitate the review process with your consultant. Please do not ask them to review your protocol separately.

For more details, see the IRB Application Instructions on Research Involving Non-English Speaking Subjects or Subjects from a Foreign Culture.

Local Requirements:

If you will conduct research at an international location, identify and describe:

- relevant local regulations
- data privacy regulations
- applicable laws
- ethics review requirements for human subject protection

Please provide links or sources where possible. If the project has been or will be reviewed by a local ethics review board, attach a copy in the “Additional Information/Materials” section. You may also consult the current edition of the International Compilation of Human Research Standards

Does your study involve **HIV/AIDS research and/or screening for other reportable diseases (e.g., Hepatitis C, etc…)**?
○ Yes ◉ No

HIV/AIDS Research

If you have questions about what constitutes a reportable disease and/or condition in the state of Kentucky, see ORI's summary sheet: "Reporting Requirements for Diseases and Conditions in Kentucky" [PDF].

HIV/AIDS Research: There are additional IRB requirements for designing and implementing the research and for obtaining informed consent. Describe additional safeguards to minimize risk to subjects in the space provided below.

For additional information, visit the online IRB Survival Handbook to download a copy of the "Medical IRB's requirements for Protection of Human Subjects in Research Involving HIV Testing" [D65.0000] [PDF], and visit the Office for Human Research Protections web site for statements on AIDS research, or contact the Office of Research Integrity at 859-257-9428.

PI-Initiated FDA-Regulated Research

Is this an investigator-initiated study that:

1) involves testing a Nonsignificant Risk (NSR) Device, or
2) is being conducted under an investigator-held Investigational New Drug (IND) or Investigational Device Exemption (IDE)?
○ Yes ◉ No

PI-Sponsored FDA-Regulated Research

If the answer above is yes, then the investigator assumes the regulatory responsibilities of both the investigator and sponsor. The Office of Research Integrity provides a summary list of sponsor IND regulatory requirements for drug trials [PDF], IDE regulatory requirements for SR device trials [PDF], and abbreviated regulatory requirements for NSR device trials [PDF]. For detailed descriptions see FDA Responsibilities for Device Study Sponsors or FDA Responsibilities for IND Drug Study Sponsor-Investigators.

- Describe the experience/knowledge/training (if any) of the investigator serving as a sponsor (e.g., previously held an IND/IDE); and
- Indicate if any sponsor obligations have been transferred to a commercial sponsor, contract research organization (CRO), contract monitor, or other entity (provide details or attach FDA 1571).

IRB policy requires mandatory training for investigators who are also FDA-regulated sponsors (see Sponsor-Investigator FAQs). A sponsor-investigator must complete the applicable Office of Research Integrity web based training, (drug or device) before final IRB approval is granted.

Has the sponsor-investigator completed the mandatory PI-sponsor training prior to this submission?
○ Yes ○ No
If the sponsor-investigator has completed equivalent sponsor-investigator training, submit documentation of the content for the IRB's consideration.

Attachments

HIPAA

0 unresolved comment(s)

Is HIPAA applicable? (•) Yes ( ) No

(Visit ORI's Health Insurance Portability and Accountability Act (HIPAA) web page to determine if your research falls under the HIPAA Privacy Regulation.)

I have attached a HIPAA Waiver of Authorization. ( ) Yes (•) No

Attachments

STUDY DRUG INFORMATION

0 unresolved comment(s)

Drugs are articles intended for use in the diagnosis, cure, mitigation, treatment, or prevention of disease and articles (other than food) intended to affect the structure or any function of the body of man or other animals.

The term drug may include:

- FDA approved drugs,
- unapproved use of approved drugs,
- investigational drugs or biologics,
- other compounds or products intended to affect structure or function of the body, and/or
- complementary and alternative medicine products such as dietary supplements, substances generally recognized as safe (GRAS) when used to diagnose, cure mitigate, treat or prevent disease, or clinical studies of e-cigarettes examining a potential therapeutic purpose.

Does this protocol involve a drug including an FDA approved drug; unapproved use of an FDA approved drug; and/or an investigational drug?

○ Yes ◉ No

If yes, complete the questions below. Additional study drug guidance.

LIST EACH DRUG INVOLVED IN STUDY IN THE SPACE BELOW

Drug Name:

Note: Inpatient studies are required by Hospital Policy to utilize Investigational Drug Service (IDS) pharmacies (Oncology or Non-Oncology). Use of IDS is highly recommended, but optional for outpatient studies. Outpatient studies not using IDS services are subject to periodic inspection by the IDS for compliance with drug accountability good clinical practices.

Indicate where study drug(s) will be housed and managed:

☐ Investigational Drug Service (IDS) UK Hospital

Other Location:

Is the study being conducted under a valid Investigational New Drug (IND) application?

○ Yes ○ No

If Yes, list IND #(s) and complete the following:

IND Submitted/Held by:

Sponsor: ☐	Held By:
Investigator: ☐	Held By:
Other: ☐	Held By:

☐ Checkmark if the study is being conducted under FDA's Expanded Access Program (e.g., Treatment IND) or if this is an Individual Patient Expanded Access IND (FDA Form 3926).

See FDA's Expanded Access Program Information for Individual Patient Expanded Access INDs, and attach the following:

- FDA Form 3926;
- FDA expanded access approval or correspondence;
- Confirmation of agreement from manufacturer or entity authorized to provide access to the product.

For guidance and reporting requirements at the conclusion of treatment see the Expanded Access SOP.

Complete and attach the required Study Drug Form picking "Study Drug Form" for the document type. Any applicable drug documentation (e.g., Investigator Brochure; approved labeling; publication; FDA correspondence, etc.) should be attached using "Other Drug Documentation" for the document type.

Attachments

STUDY DEVICE INFORMATION

0 unresolved comment(s)

Medical devices are intended for use in the diagnosis of disease or other conditions, or in the cure, mitigation, treatment, or prevention of disease, in man or other animals, or intended to affect the structure or any function of the body of man or other animals.

A DEVICE may be a:

- component, part, accessory;
- assay, reagent, or in-vitro diagnostic device;
- software, digital health, or mobile medical app;
- other instrument if intended to affect the structure or function of the body, diagnose, cure, mitigate, treat or prevent disease; or
- a homemade device developed by an investigator or other non-commercial entity and not approved for marketing by FDA.

For additional information, helpful resources, and definitions, see ORI's Use of Any Device Being Tested in Research web page.

Does this protocol involve testing (collecting safety or efficacy data) of a medical device including an FDA approved device, unapproved use of an approved device, humanitarian use device, and/or an investigational device?

(•) Yes ( ) No

[Note: If a marketed device(s) is only being used to elicit or measure a physiologic response or clinical outcome, AND, NO data will be collected on or about the device itself, you may answer "no" above, save and exit this section, (Examples: a chemo drug study uses an MRI to measure tumor growth but does NOT assess how effective the MRI is at making the measurement; an exercise study uses a heart monitor to measure athletic performance but no safety or efficacy information will be collected about the device itself, nor will the data collected be used for comparative purposes against any other similar device).]

If you answered yes above, please complete the following questions.

LIST EACH DEVICE BEING TESTED IN STUDY IN THE SPACE BELOW

Device Name:

SCONE system (SpineX Inc., Northridge, CA, USA)/ ARCEX (Onward Medical, Boston, USA).

The study is evaluating the safety, feasibility, and preliminary efficacy of transcutaneous spinal cord stimulation (tSCS) delivered with a SpineX/ ARCEX neuromodulation device in individuals with HSP. This constitutes a collection of safety/efficacy data on a medical device.

Is the study being conducted under a valid Investigational Device Exemption (IDE), Humanitarian Device Exemption (HDE) or Compassionate Use?

( ) Yes (•) No

If Yes, complete the following:
IDE or HDE #(s)

IDE/HDE Submitted/Held by:

Sponsor: ☐ Held By:

Investigator: ☐ Held By:

Other: ☐ Held By:

☐ Check if this is a Treatment IDE or Compassionate Use under the Food and Drug Administration (FDA) Expanded Access program.

For Individual or Small Group Expanded Access, see FDA's Early Expanded Access Program Information, and attach the following:

- FDA expanded access approval or sponsor's authorization;
- An independent assessment from an uninvolved physician, if available;
- Confirmation of agreement from manufacturer or entity authorized to provide access to the product.

For guidance and reporting requirements at the conclusion of treatment see the Medical Device SOP.

Does the intended use of any research device being tested (not clinically observed) in this study meet the regulatory definition [FDA's PDF] of Significant Risk (SR) device?

- ○ Yes. Device(s) being tested in this study presents a potential for serious risk to the health, safety, or welfare of a subject and (1) is intended as an implant; or (2) is used in supporting or sustaining human life; or (3) is of substantial importance in diagnosing, curing, mitigating or treating disease, or otherwise prevents impairment of human health; or (4) otherwise presents a potential for serious risk to the health, safety, or welfare of a subject.
- ◉ No. All devices being tested in this study do not present a potential for serious risk to the health, safety, or welfare of subjects/participants.

Complete and attach the required Study Device Form, picking the "Study Device Form" for the document type. Any applicable device documentation (e.g., Manufacturer information; patient information packet; approved labeling; FDA correspondence, etc.) should be attached using "Other Device Documentation" for the document type.

Attachments

Attach Type	File Name
Other Device Documentation	030-0310 (Rev C) - SCONE IFU_251223_214933.pdf
Other Device Documentation	ARC-EX-info.pdf
Other Device Documentation	IFU_Professional_ARC-EX-EXIFU01PROEN-v5.pdf
Study Device Form	study-device-attachment_SCONE.pdf
Study Device Form	study-device-attachment_ARCex.pdf

RESEARCH SITES

0 unresolved comment(s)

To complete this section, ensure the responses are accurate then click "SAVE".

A) Check all the applicable sites listed below at which the research will be conducted. If none apply, you do not need to check any boxes.

UK Sites

- ☑ UK Classroom(s)/Lab(s)
- ☐ UK Clinics in Lexington
- ☐ UK Clinics outside of Lexington
- ☐ UK Healthcare Good Samaritan Hospital
- ☐ UK Hospital

Schools/Education Institutions

- ☐ Fayette Co. School Systems *
- ☐ Other State/Regional School Systems
- ☐ Institutions of Higher Education (other than UK)

***Fayette Co. School systems, as well as other non-UK sites, have additional requirements that must be addressed. See ORI's IRB Application Instructions - Off-site Research web page for details.**

Other Medical Facilities

- ☐ Bluegrass Regional Mental Health Retardation Board
- ☑ Cardinal Hill Hospital
- ☐ Eastern State Hospital
- ☐ Norton Healthcare
- ☐ Nursing Homes
- ☐ Shriner's Children's Hospital
- ☐ Veterans Affairs Medical Center
- ☐ Other Hospitals and Med. Centers

- ☐ Correctional Facilities
- ☐ Home Health Agencies
- ☐ International Sites

Research activities conducted at performance sites that are not owned or operated by the University of Kentucky (UK) or at sites that do not fall under the UK IRB's authority, are subject to special procedures for coordination of research review. Additional information is required (see IRB Application Instructions - Off-Site Research web page), including:

- A letter of support and local context is required from non-UK sites. See *Letters of Support and Local Context* on the IRB Application Instructions - Off-Site Research web page for more information.
- Supportive documentation, including letters of support, can be attached below. When attaching reliance documents, please ensure that you select the correct 'Document Type' from the drop-down menu. See below for the "**Document Types**" in bold, followed by examples of reliance documents for each type:
  - **- Individual Investigator Agreement (IIA)**
    - A completed Individual Investigator Agreement

- IRB Approval (Non-UK)
- A Letter of Approval from a Non-UK IRB

- IRB Authorization Agreement (IAA)
- A SMART IRB Agreement
- An OHRP Agreement
- A DoD Agreement
- An IREx Reliance Notification
- Any Reliance Agreement

- Letter of Support & Local Context
- A Letter of Support from an organization at which some research activities are occurring
- Communications Plan
- Local Context Form

Please reach out to IRBReliance@uky.edu if you have any questions or concerns.

- NOTE: If the non-UK sites or non-UK personnel are engaged in the research, there are additional federal and university requirements which need to be completed for their participation. For instance, the other site(s) may need to complete their own IRB review, or a cooperative review arrangement may need to be established with non-UK sites.
- Questions about the participation of non-UK sites/personnel should be discussed with the ORI staff at (859) 257-9428.

List all other non-UK owned/operated locations where the research will be conducted:

Plasma aliquot will be shipped to Drexel University for the identification of Big tau as a potential biomarker for hereditary spastic paraplegia (HSP) under the direction of Dr. Emanuela Piermarini. All samples will be de-identified before shipment. Dr. Piermarini and her research team will have no interaction with study participants and will not have access to identifiable participant information.

Describe the role of any non-UK site(s) or non-UK personnel who will be participating in your research.

Dr. Rich Cohn, Biostatistician, will serve as an independent consultant and will assist with data analysis. He will have no interaction with study participants and will not have access to identifiable participant information.

Please describe the plan for the management of reporting unanticipated problems, noncompliance, and submission of protocol modifications and interim results from the non-UK sites:

Activities conducted by non-University of Kentucky collaborators involve only the analysis of de-identified data or de-identified biospecimens and do not include any interaction with study participants or access to identifiable information. Dr. Rich Cohn will assist solely with statistical analysis of de-identified datasets, and Drexel University will receive only de-identified plasma samples for biomarker analysis. As such, the risk of unanticipated problems, noncompliance, or protocol deviations at non-UK sites is minimal. Any unexpected issues related to data integrity or protocol conduct that could impact the study will be promptly communicated to the University of Kentucky Principal Investigator and reported to the UK IRB in accordance with institutional requirements.

Attachments

B) If your research involves collaboration with any sites and/or personnel outside the University of Kentucky, then it is considered multisite research and IRB reliance issues will need to be addressed. This may include national multi-center trials as well local studies involving sites/personnel external to UK. If you would like to request that the University of Kentucky IRB (UK IRB) serve as the lead IRB for your study, or if you would like the UK IRB to defer review to another IRB, please contact the IRBReliance@uky.edu.

RESEARCH ATTRIBUTES

0 unresolved comment(s)

Instructions: For various reasons, it is necessary to determine whether your research activities meet the definition of clinical research and/or a clinical trial. Your responses to the next series of questions will make that determination. For more details on the definitions, go to ORI's clinical research vs. clinical trial web page or visit NIH's decision tree for the NIH Clinical Trial definition.

Contact the Clinical Research Support Office (CRSO) if your study provides clinical services (e.g., labs, biopsies, tissue samples, physical exams, PT, counseling) regardless of payer (grant, federal, UK, industry), utilizes UKHC space, or meets the NIH definition of a clinical trial (thereby requiring registry with CT.gov) as your study will need to be entered in OnCore to ensure appropriate regulatory tracking and billing. Visit CRSO FAQs for more information; requests for CCTS/CRSO services can be submitted via their service request form. For other questions, you can contact the CRSO Director, Jessica Heskel.

My research activities include one or more of the following:

Patient-oriented research regarding mechanisms of human disease, therapeutic interventions, clinical studies, or development of new technologies

(•) Yes ( ) No

Material of human origin (such as tissues, specimens, and cognitive phenomena) for which an investigator (or colleague) directly interacts with human subjects

(•) Yes ( ) No

Epidemiologic or Behavioral Studies

(•) Yes ( ) No

Outcomes Research or Health Services Research

(•) Yes ( ) No

Does your research study involve one or more human subjects prospectively assigned into one or more interventions (which may include placebo or other control) to evaluate the effects of those interventions on health-related biomedical or behavioral outcomes? **If yes, your study meets the NIH definition of a clinical trial.**

(•) Yes ( ) No

Indicate the items below that apply to your research. Depending on the items applicable to your research, you may be required to complete additional forms or meet additional requirements. Contact the ORI (859-257-9428) if you have questions about additional requirements.

[ ] Not applicable

Check All That Apply

- [ ] Academic Degree/Required Research
- [ ] Alcohol/Drug/Substance Abuse Research
- [ ] Biological Specimen Bank Creation (for sharing)
- [ ] Cancer Research
- [ ] CCTS-Center for Clinical & Translational Science
- [ ] Certificate of Confidentiality
- [x] Collection of Biological Specimens for banking and use
- [ ] Community-Based Participatory Research
- [ ] Deception
- [ ] Educational/Student Records (e.g., GPA, test scores)
- [ ] Emergency Use (Single Patient)
- [ ] Gene Transfer

For additional requirements and information:

- Cancer Research (MCC PRMC)
- Certificate of Confidentiality (look up "Confidentiality/Privacy...")
- CCTS (Center for Clinical and Translational Science)
- Clinical Research (look up "What is the definition of....)
- Clinical Trial
- Collection of Biological Specimens for Banking (look up "Banks, Repositories, Registries...")
- Collection of Biological Specimens (look up "Repositories, Registries, Specimen/Tissue Banks...")
- Community-Based Participatory Research (look up "Community-Engaged...")
- Data & Safety Monitoring Board (DSMB)

  *For Medical IRB: Service Request Form for CCTS DSMB

- Data & Safety Monitoring Plan
- Deception*

☑ Genetic Research
☐ NIH Genomic Data Sharing (GDS) (databases such as GWAS, dbGaP, GenBank)
☐ Treatment with Human Cells, Tissues, and Cellular and Tissue Based Products
☐ Individual Expanded Access or Compassionate Use
☐ International Research
☐ Planned Emergency Research Involving Exception from Informed Consent
☐ Recombinant DNA
☐ Registry or data repository creation
☐ Stem Cell Research
☐ Suicide Ideation or Behavior Research
☐ Survey Research
☐ Transplants
☐ Use, storage and disposal of radioactive material and radiation producing devices
☐ Vaccine Trials

*For deception research, also go to the E-IRB Application Informed Consent section, checkmark and complete "Request for Waiver of Informed Consent Process"

- Emergency Use (Single Patient) [attach Emergency Use Checklist] (PDF)
- Genetic Research (look up "Banks, Repositories, ...Genetic/Genomic Data Sharing...")
- Gene Transfer

*For gene transfer research, also go to the E-IRB Application Other Review Committees section, and checkmark Institutional Biosafety Committee

- International Research (look up "International & Non-English Speaking")
- NIH Genomic Data Sharing (GDS) Policy (PDF)
- Planned Emergency Research Involving Exception to Informed Consent*

*For Planned Emergency Research Involving Waiver of Informed Consent, also go to the E-IRB Application Informed Consent section, checkmark and complete "Request for Waiver of Informed Consent Process"

- Use, storage and disposal of radioactive material and radiation producing devices

FUNDING/SUPPORT

0 unresolved comment(s)

If the research is being submitted to, supported by, or conducted in cooperation with an external or internal agency or funding program, indicate below all the categories that apply.

☐ Not applicable

Check All That Apply

☐ Grant application pending
☐ (HHS) Dept. of Health & Human Services
☐ (NIH) National Institutes of Health
☐ (CDC) Centers for Disease Control & Prevention
☐ (HRSA) Health Resources and Services Administration
☐ (SAMHSA) Substance Abuse and Mental Health Services Administration
☐ (DoJ) Department of Justice or Bureau of Prisons
☐ (DoE) Department of Energy
☐ (EPA) Environmental Protection Agency
☐ Federal Agencies Other Than Those Listed Here
☐ Industry (Other than Pharmaceutical Companies)
☐ Internal Grant Program w/ proposal
☐ Internal Grant Program w/o proposal
☐ National Science Foundation
☐ Other Institutions of Higher Education
☐ Pharmaceutical Company
☑ Private Foundation/Association
☐ U.S. Department of Education
☐ State

Click applicable listing(s) for additional requirements and information:

- (HHS) Dept. of Health & Human Services
- (NIH) National Institutes of Health
- (CDC) Centers for Disease Control & Prevention
- (HRSA) Health Resources & Services Administration
- (SAMHSA) Substance Abuse & Mental Health Services Administration
- Industry (Other than Pharmaceutical Companies) [IRB Fee Info-look up "Does the IRB Charge a Fee..."]
- National Science Foundation
- (DoEd) U.S. Department of Education
- (DoJ) Department of Justice or Bureau of Prisons
- (DoE) Department of Energy Summary and Department of Energy Identifiable Information Compliance Checklist
- (EPA) Environmental Protection Agency

Other:

Specify the funding source and/or cooperating organization(s) (e.g., National Cancer Institute, Ford Foundation, Eli Lilly & Company, South Western Oncology Group, Bureau of Prisons, etc.):

The Spastic Paraplegia Foundation

Add Related Grants

If applicable, please search for and select the OSPA Account number or Electronic Internal Approval Form (eIAF) # (notif #) associated with this IRB application using the "Add Related Grants" button.
If required by your funding agency, upload your grant using the "Grant/Contract Attachments" button.

Add Related Grants

Grant/Contract Attachments

The research involves use of Department of Defense (DoD) funding, military personnel, DoD facilities, or other DoD resources. (See DoD SOP and DoD Summary for details)

○ Yes ◉ No

Using the “attachments” button (below), attach applicable materials addressing the specific processes described in the DoD SOP.

DOD SOP Attachments

Additional Certification: (If your project is federally funded, your funding agency may request an Assurance/ Certification/Declaration of Exemption form.) Check the following if needed:

☐ Protection of Human Subjects Assurance/Certification/Declaration of Exemption (Formerly Optional Form – 310)

Assurance/Certification Attachments

OTHER REVIEW COMMITTEES

0 unresolved comment(s)

If you check any of the below committees, additional materials may be required with your application submission.

Does your research fall under the purview of any of the other review committees listed below? [*If yes, check all that apply and attach applicable materials using the attachment button at the bottom of your screen.*]

◉ Yes ○ No

Additional Information

- [x] Institutional Biosafety Committee
- [ ] Radiation Safety Committee
- [ ] Radioactive Drug Research Committee
- [ ] Markey Cancer Center (MCC) Protocol Review and Monitoring Committee (PRMC)
- [ ] Graduate Medical Education Committee (GME)
- [ ] Office of Medical Education (OME)

- Institutional Biosafety Committee (IBC) - Attach required IBC materials
- Radiation Safety Committee (RSC) - For applicability, see instructions
- Radioactive Drug Research Committee (RDRC)
- Markey Cancer Center (MCC) Protocol Review and Monitoring Committee (PRMC)** - Attach MCC PRMC materials, if any, per instructions.
- Office of Medical Education (OME)
- Graduate Medical Education Committee (GME)

Attachments

**** If your study involves cancer research, be sure to select "Cancer Research" in the "Research Attributes" section.** ORI will send your research protocol to the Markey Cancer Center (MCC) Protocol Review and Monitoring Committee (PRMC). The MCC PRMC is responsible for determining whether the study meets the National Cancer Institute (NCI) definition of a clinical trial and for issuing documentation to you (the investigator) which confirms either that PRMC approval has been obtained or that PRMC review is not required. Your IRB application will be processed and reviewed independently from the PRMC review.

ADDITIONAL INFORMATION/MATERIALS

0 unresolved comment(s)

Do you want specific information inserted into your approval letter? ◯ Yes ◉ No

Approval Letter Details:

If you wish to have specific language included in your approval letter (e.g., serial #, internal tracking identifier, etc…), type that language in the box below exactly as it should appear in the letter. The text you enter will automatically appear at the top of all approval letters, identical to how you typed it, until you update it. Don't include instructions or questions to ORI staff as those will appear in your approval letter. **If these details need to be changed for any reason, you are responsible for updating the content of this field.**

Additional Materials:

If you have other materials you would like to include for the IRB's consideration, check all that apply and attach the corresponding documents using the Attachments button below.

☐ Detailed protocol
☐ Dept. of Health & Human Services (DHHS) approved protocol (such as NIH sponsored Cooperative Group Clinical Trial)
☐ Other Documents

Protocol/Other Attachments

Attach Type	File Name
Other	IRB Requested Revisions.pdf
Other	HSP_Collection_Form.pdf
Other	Clean_6MWT_Wheelchair_Endurance_CRF_HSP_tSCS.pdf
Other	Highlighted_6MWT_Wheelchair_Endurance_CRF_HSP_tSCS.pdf
Other	Clean_10MWT_CRF_HSP_tSCS.pdf
Other	Clean_10MWT_Wheelchair_Speed_CRF_HSP_tSCS.pdf
Other	Highlighted_10MWT_CRF_HSP_tSCS.pdf
Other	Highlighted_10MWT_Wheelchair_Speed_CRF_HSP_tSCS.pdf
Other	Clean_HSP-SNAP.pdf
Other	Highlighted_HSP-SNAP.pdf
Other	Clean_MAS_CRF_HSP_tSCS.pdf
Other	Highlighted_MAS_CRF_HSP_tSCS.pdf
Other	Clean_NBDS score.pdf
Other	Highlighted_NBDS score.pdf
Other	Clean_NBSS English.pdf
Other	Highlighted_NBSS English.pdf
Other	Clean_SPRS_e1.pdf
Other	Highlighted_SPRS_e1.pdf
Protocol	HSP_tSCS_Blood_SOP_Sachdeva.pdf

NOTE: Instructions for Dept. of Health & Human Services (DHHS)-approved protocol]

If you have password protected documents, that feature should be disabled prior to uploading to ensure access for IRB review.

To view the materials currently attached to your application, click “All Attachments” on the left menu bar.

SIGNATURES (ASSURANCES)

0 unresolved comment(s)

Introduction

All IRB applications require additional assurances by a Department Chairperson or equivalent (DA), and when applicable, a Faculty Advisor or equivalent (FA). This signifies the acceptance of certain responsibilities and that the science is meritorious and deserving of conduct in humans. The person assigned as DA *should not* also be listed in the Study Personnel section, and the individual assigned as FA *should* be listed in the Study Personnel section.

For a list of responsibilities reflected by signing the Assurance Statement, refer to "What does the Department Chairperson's Assurance Statement on the IRB application mean?"

For a detailed illustration of how to complete this section, please review the short online video tutorial "Signatures (Assurance) Section - How to Complete." Otherwise, follow the steps below.

Required Signatures:

Individuals chosen as signees may remove the application from their Inbox without signing the Assurance Statement by clicking "Return to PI" with a comment about why it is being returned (e.g., specific edits are deemed necessary).

The PI, and personnel chosen as a contact, will receive an email notification that edits are needed, and can find the draft application in both the "Draft" folder and the "Signatures Status" folder located in the menu in the left margin of the default Inbox page. The researcher does not have a 'reply' option to the signee's comments and must make the requested edits directly in the application, or communicate outside the E-IRB system as to why not. Once the response is finalized, the researcher must re-visit the "Assurances Required" section to click the "Return to Signee" button for their re-consideration; the signee will receive an email notification at that time.

Hover your mouse cursor here for additional instructions.

First Name	Last Name	Role	Department	Signee Return Comment	Date Signed	
Susan	McDowell	Department Authorization	Rehabilitation Medicine		12/23/2025 04:09 PM	View/Sign
Rahul	Sachdeva	Principal Investigator	Physical Medicine & Rehab - Research Div		12/23/2025 02:49 PM	View/Sign

Department Authorization

☑ This is to certify that I have reviewed this research protocol and that I attest to the scientific validity and importance of this study; to the qualifications of the investigator(s) to conduct the project and their time available for the project; that facilities, equipment, and personnel are adequate to conduct the research; and that continued guidance will be provided as appropriate. When the principal investigator assumes a sponsor function, the investigator has been notified of the additional regulatory requirements of the sponsor and by signing the principal investigator Assurance Statement, confirms he/she can comply with them.

*If the Principal Investigator is also the Chairperson of the department, the Vice Chairperson or equivalent should complete the "Department Authorization".

**IF APPLICABLE FOR RELIANCE: I attest that the principal investigator has been notified of the regulatory requirements of both the Reviewing and Relying IRBs, according to the information provided in the E-IRB application. The attached Reliance Assurance Statement, signed by the principal investigator, confirms that he/she can comply with both sets of IRB requirements.

Principal Investigator's Assurance Statement

I understand the University of Kentucky's policies concerning research involving human subjects and I agree:

1. To comply with all IRB policies, decisions, conditions, and requirements;
2. To accept responsibility for the scientific and ethical conduct of this research study;
3. To obtain prior approval from the Institutional Review Board before amending or altering the research protocol or implementing changes in the approved consent/assent form;
4. To report to the IRB in accord with IRB/IBC policy, any adverse event(s) and/or unanticipated problem(s) involving risks to subjects;
5. To complete, on request by the IRB for Full and Expedited studies, the Continuation/Final Review Forms;
6. To notify the Office of Sponsored Projects Administration (OSPA) and/or the IRB (when applicable) of the development of any financial interest not already disclosed;
7. Each individual listed as study personnel in this application has received the mandatory human research protections education (e.g., CITI);
8. Each individual listed as study personnel in this application possesses the necessary experience for conducting research activities in the role described for this research study.
9. To recognize and accept additional regulatory responsibilities if serving as both a sponsor and investigator for FDA regulated research.

☑ Furthermore, by checking this box, I also attest that:

- I have appropriate facilities and resources for conducting the study;
- I am aware of and take full responsibility for the accuracy of all materials submitted to the IRB for review;
- If applying for an exemption, I also certify that the only involvement of human subjects in this research study will be in the categories specified in the Protocol Type: Exemption Categories section.
- If applying for an Abbreviated Application (AA) to rely on an external IRB, I understand that certain items above (1, 3, 4, 7-8) may not apply, or may be altered due to external institutional/IRB policies. I document my agreement with the Principal Investigator Reliance Assurance Statement by digitally signing this application.

*You will be able to "sign" your assurance after you have sent your application for signatures (use Submission section). Once all Assurance Statement signatures have been acquired, return to this section to submit your application to ORI.

SUBMISSION INFORMATION

0 unresolved comment(s)

Each Section/Subsection in the menu on the left must have a checkmark beside it (except this Submission section) indicating the Section/Subsection has been completed. Otherwise your submission for IRB review and approval cannot be sent to the Office of Research Integrity/IRB.

If applicable, remember to update the Approval Letter Details text box under the Additional Information section

If your materials require review at a convened IRB meeting which you will be asked to attend, it will be scheduled on the next available agenda and you will receive a message to notify you of the date.

If you are making a change to an attachment, you need to delete the attachment, upload a highlighted version that contains the changes (use Document Type of "Highlighted Changes"), and a version that contains the changes without any highlights (use the appropriate Document Type for the item(s)). Do **not** delete approved attachments that are still in use.

Your protocol has been submitted.

Download all

	Document Type	File Loaded	Document Description	File Size	Modified By	Mod Date
	ApprovalLetter	ApprovalLetter.pdf		0.083	scbe223	1/21/2026 3:19:18 PM
	Stamped Consent Form	Clean_Informed-Consent-form_SCS-HSP_Sachdeva_Rev1.pdf		0.484	scbe223	1/21/2026 3:19:18 PM
	InformedConsent_HighlightedChanges	Highlighted_Informed-Consent-form_SCS-HSP_Sachdeva_Rev1.pdf	Revised file with track changes (highlighted file)	0.420	scbe223	1/20/2026 3:34:46 PM
	AddInfoProduct	Highlighted_SPRS_e1.pdf	highlighted (in yellow) SPRS	0.310	vkpa225	1/16/2026 4:36:48 PM
	AddInfoProduct	Clean_SPRS_e1.pdf	clean file for SPRS	0.297	vkpa225	1/16/2026 4:35:53 PM
	AddInfoProduct	Highlighted_NBSS English.pdf	highlighted (in yellow) NDSS Score	0.118	vkpa225	1/16/2026 4:34:07 PM
	AddInfoProduct	Clean_NBSS English.pdf	clean file for NBSS Score	0.202	vkpa225	1/16/2026 4:33:43 PM
	AddInfoProduct	Highlighted_NBDS score.pdf	highlighted (in yellow) NBDS Score	0.088	vkpa225	1/16/2026 4:31:21 PM
	AddInfoProduct	Clean_NBDS score.pdf	clean file for NBDS Score	0.107	vkpa225	1/16/2026 4:30:59 PM
	AddInfoProduct	Highlighted_MAS_CRF_HSP_tSCS.pdf	highlighted (in yellow) MAS	0.157	vkpa225	1/16/2026 4:28:24 PM
	AddInfoProduct	Clean_MAS_CRF_HSP_tSCS.pdf	Clean file for MAS	0.141	vkpa225	1/16/2026 4:28:02 PM
	AddInfoProduct	Highlighted_HSP-SNAP.pdf	highlighted (in yellow) HSP-SNAP revised	0.221	vkpa225	1/16/2026 4:21:21 PM
	AddInfoProduct	Clean_HSP-SNAP.pdf	Clean file for HSP-SNAP	0.219	vkpa225	1/16/2026 4:20:35 PM
	AddInfoProduct	Highlighted_10MWT_Wheelchair_Speed_CRF_HSP_tSCS.pdf	highlighted (in yellow) 10 MWT_wheelchair test	0.174	vkpa225	1/16/2026 4:17:23 PM
	AddInfoProduct	Highlighted_10MWT_CRF_HSP_tSCS.pdf	highlighted (in yellow) 10 MWT	0.144	vkpa225	1/16/2026 4:15:19 PM
	AddInfoProduct	Clean_10MWT_Wheelchair_Speed_CRF_HSP_tSCS.pdf	Clean file for wheelchair Speed (alternate for 10 MWT)	0.183	vkpa225	1/16/2026 4:13:35 PM
	AddInfoProduct	Clean_10MWT_CRF_HSP_tSCS.pdf	Clean file for 10 MWT	0.145	vkpa225	1/16/2026 4:12:43 PM
	AddInfoProduct	Highlighted_6MWT_Wheelchair_Endurance_CRF_HSP_tSCS.pdf	highlighted (in yellow) 6MWT	0.194	vkpa225	1/16/2026 4:07:22 PM
	AddInfoProduct	Clean_6MWT_Wheelchair_Endurance_CRF_HSP_tSCS.pdf	clean file for 6MWT	0.196	vkpa225	1/16/2026 4:04:29 PM
	AddInfoProduct	HSP_Collection_Form.pdf	New form to capture data related to mobility, pain, gait test and blood collection	0.201	vkpa225	1/16/2026 4:00:10 PM
	ResearchProcedures	Clean_Visit by Visit procedures_SCS-HSP_Sachdeva_rev-1.pdf	clean version of Visit to Visit procedures	0.119	vkpa225	1/16/2026 3:57:32 PM
	ResearchProcedures	Highlighted Visit by Visit procedures_SCS-HSP_Sachdeva_rev-1.pdf	revised Visit by Visit procedures with track changes (highlighted)	0.143	vkpa225	1/16/2026 3:52:29 PM
	Informed ConsentHIPAA Combined Form	Clean_Informed-Consent-form_SCS-HSP_Sachdeva_Rev1.pdf	Clean file after revision (without track changes)	0.363	vkpa225	1/16/2026 3:23:56 PM
	AddInfoProduct	IRB Requested Revisions.pdf	IRB Requested Revisions	0.241	scbe223	1/15/2026 2:33:09 PM
	Advertising	HSP Flyer.pdf	Flyer for advertising	1.107	vkpa225	12/23/2025 3:15:42 PM
	AddInfoProtocol	HSP_tSCS_Blood_SOP_Sachdeva.pdf	Blood collection protocol	0.130	vkpa225	12/23/2025 1:42:26 PM

	OtherDeviceDocumentation	IFU_Professional_ARC-EX-EXIFU01PROEN-v5.pdf	Supporting doc for ArcEX	6.125	vkpa225	12/23/2025 1:37:35 PM
	OtherDeviceDocumentation	ARC-EX-info.pdf	Manufacturer Information for USE_ArcEx	0.486	vkpa225	12/23/2025 1:36:51 PM
	OtherDeviceDocumentation	030-0310 (Rev C) - SCONE IFU_251223_214933.pdf	Manufacturer Information for USE_SCONE device	3.057	vkpa225	12/23/2025 1:36:00 PM
	StudyDevice	study-device-attachment_ARCex.pdf	ARCex device	0.642	vkpa225	12/23/2025 1:31:04 PM
	StudyDevice	study-device-attachment_SCONE.pdf	SCONE device	0.632	vkpa225	12/23/2025 1:29:50 PM
	Advertising	Recruitment and Advertising materials.pdf		0.087	vkpa225	12/23/2025 10:39:59 AM

Click link to sort Changed Date

Informed Consent ElectronicConsent changed by vkpa225 on 1/16/2026 10:28:49 AM

~~N~~Y

Informed Consent InformedConsentProcess changed by vkpa225 on 1/16/2026 10:28:49 AM

Each prospective participant will be given adequate time to read the informed consent document and discuss the study with the research team. A member of the study team will review the consent form with the participant, answer all study-related questions, and confirm understanding of study procedures, risks, benefits, and the voluntary nature of participation. Written informed consent will be obtained prior to performing any study-specific procedures. After the consent form is signed, a copy of the signed informed consent document will be provided to the participant. ¶
¶
Participants may complete the informed consent process in one of two ways: ¶
(1) by attending an in-person visit, during which the consent form will be reviewed and signed, or ¶
(2) by receiving the IRB-approved consent form via email. Participants who receive the consent form electronically will be given sufficient time to review the document, ask questions of the Principal Investigator or study personnel, and may return the signed consent form via email or bring the signed copy to their first baseline assessment visit (visit 2). This option is intended to reduce the number of required study visits for participants who live at a greater distance from the study site. ¶
Written informed consent will be obtained prior to the initiation of any study-specific procedures. After the consent form is signed, a copy of the signed informed consent document will be provided to the participant. ¶
¶
To minimize the possibility of coercion or undue influence, only individuals explicitly designated in the E-IRB "Study Personnel" section as authorized to obtain informed consent will conduct the consent process. ¶
Specifically, consent will be obtained only by: ¶
Dr. Rahul Sachdeva ¶
Dr. Sara Salles ¶
Dr. Zakari Dymock ¶
Dr. Susan McDowell ¶
Dr. Vivek Pandey ¶
Ms. Makyra Ross ¶
¶
The consent discussion will occur in a private setting (or other appropriate confidential location), and participants will be reminded that participation is voluntary, they may decline to participate or withdraw at any time without penalty or loss of benefits to which they are otherwise entitled, and they are free to ask questions at any time. ¶
Participants will be provided the Principal Investigator's contact information during the consent process and will also be informed they may contact the University of Kentucky Office of Research Integrity (ORI) at 859-257-9428 or toll free at 1-866- 400-9428 with any concerns.

Research Attributes GeneticResearch changed by vkpa225 on 1/16/2026 12:49:57 PM

Y

Research Description CostsToSubjects changed by vkpa225 on 1/16/2026 12:36:57 PM

Participants and/or their insurance company, Medicare, or Medicaid will be responsible for the costs of all care and treatment that participants would normally receive for any conditions they may have. These are costs that are considered medically necessary and will be part of the care they receive, even if they do not take part in this study. ¶
The University of Kentucky may not be allowed to bill participants' insurance company, Medicare, or Medicaid for the medical procedures done strictly for research. The research procedures in this study include the spinal cord stimulation sessions (tSCS), mobility and strength assessments, research questionnaires, and laboratory testing done only for research. Therefore, this research ~~only costs~~ will be paid by the University of Kentucky and will not be billed to the participant or their insurance. ¶
If the participant receives routine medical care during the study that is not part of the research, those costs may be billed to the participant or their insurance. ~~p~~Participants' insurers, Medicare, or Medicaid, may agree to pay for the costs. However, a co-payment or deductible may be needed from the participant. The amount of this co-payment or deductible may be costly.

Research Description DataCollection changed by vkpa225 on 1/16/2026 12:35:05 PM

1. In-person clinical and functional assessments ¶
• Method: Participants will complete standardized physical and mobility/functional tests at the Human Performance Laboratory/Biodynamics Lab at baseline (Week 1), mid-intervention (week 4), post-intervention (week 8), and follow-up (week 16), with brief safety checks at each stimulation visit. ¶
• Rationale: These measures quantify motor function, mobility, strength, and spasticity to evaluate feasibility, safety, and preliminary efficacy of tSCS in HSP across diverse mobility levels (ambulatory and wheelchair users). ¶
¶
2. Participant-reported questionnaires ¶
• Method: Surveys administered in Weeks 1, 4, 8, and 16. ¶
• Rationale: Captures symptoms and functional impact of HSP from the participant's perspective, complementing clinician-rated and performance outcomes. ¶
¶
3. Biomechanical / neurophysiological recordings (as applicable to mobility level) ¶
• Method: 3D gait/movement analysis and surface EMG collected during mobility tasks at assessment visits. ¶
• Rationale: Provides objective mechanistic/physiologic markers of motor control and changes in neuromuscular activation patterns after tSCS. For wheelchair users, equivalent movement tasks will be used. ¶
¶
4. Venous blood specimens ¶
• Method: Blood draws (~10mL) at Weeks 1, 4, 8, and 16; samples processed and de-identified for laboratory analyses. ¶
• Rationale: Enables metabolomic/lipid profiling and biomarker assays (including Big-tau and related markers) to explore biological correlates of tSCS response and disease state. ¶
¶
All data to be collected: ¶
1. In-person assessments ¶
• Demographics collected at baseline: age, sex, race/ethnicity, disease duration, genetic subtype (if known), phenotype (pure vs. complex), assistive device use/mobility status. ¶
• Mobility/function outcomes: ¶
o 10-Meter Walk Test or wheelchair speed equivalent ¶
o 6-Minute Walk Test or wheelchair endurance equivalent ¶
o Sit-to-Stand test ¶
• Strength measures: ¶
o Lower-limb dynamometry or standardized manual muscle testing ¶
• Spasticity measures: ¶
o Modified Ashworth Scale (MAS) ¶
o Clonus (if present) ¶
• Clinician-rated disease severity: ¶
o Spastic Paraplegia Rating Scale (SPRS) ¶
• Session-level safety/comfort logs: ¶
o adverse events, skin checks, discomfort ratings, stimulation tolerability. ¶
2. Questionnaires ¶
• HSP-SNAP (symptom frequency/severity) ¶
• • NBSS and NBDS assessments for lower urinary tract and bowel function assessment ¶
• Falls history and mobility-confidence items if included in CRFs. ¶
3. Biomechanics / EMG ¶
• 3D kinematics/kinetics (joint angles, spatiotemporal gait parameters) for ambulatory participants ¶
• Equivalent seated mobility/movement kinematics for wheelchair users as applicable ¶
• Surface EMG from lower-limb muscles during tasks (timing, amplitude, co-contraction indices). ¶
4. Blood specimens ¶
• Metabolomic and lipid profiling outputs (de-identified) ¶
• Protein/biomarker assays including Big-tau (de-identified) ¶
• Any genetic-ally related research analyses performed on de-identified samples.

Research Description DataCollection changed by vkpa225 on 1/16/2026 12:20:02 PM

1. In-person clinical and functional assessments ¶
• Method: Participants will complete standardized physical and mobility/functional tests at the ~~UK PM&R Research~~Human Performance Laboratory/Biodynamics Lab at baseline (Week 1), mid-intervention (week 4), post-intervention (week 8), and follow-up (week 16), with brief safety checks at each stimulation visit. ¶
• Rationale: These measures quantify motor function, mobility, strength, and spasticity to evaluate feasibility, safety, and preliminary efficacy of tSCS in HSP across diverse mobility levels (ambulatory and wheelchair users). ¶
¶
2. Participant-reported questionnaires ¶
• Method: Surveys administered in Weeks 1, 4, 8, and 16. ¶
• Rationale: Captures symptoms and functional impact of HSP from the participant's perspective, complementing clinician-rated and performance outcomes. ¶
¶
3. Biomechanical / neurophysiological recordings (as applicable to mobility level) ¶
• Method: 3D gait/movement analysis and surface EMG collected during mobility tasks at assessment visits. ¶
• Rationale: Provides objective mechanistic/physiologic markers of motor control and changes in neuromuscular activation patterns after tSCS. For wheelchair users, equivalent movement tasks will be used. ¶
¶
4. Venous blood specimens ¶
• Method: Blood draws (~10mL) at Weeks 1, 4, 8, and 16; samples processed and de-identified for laboratory analyses. ¶
• Rationale: Enables metabolomic/lipid profiling and biomarker assays (including Big-tau and related markers) to explore biological correlates of tSCS response and disease state. ¶
¶
All data to be collected: ¶
1. In-person assessments ¶
• Demographics collected at baseline: age, sex, race/ethnicity, disease duration, genetic subtype (if known), phenotype (pure vs. complex), assistive device use/mobility status. ¶
• Mobility/function outcomes: ¶
o 10-Meter Walk Test or wheelchair speed equivalent ¶
o 6-Minute Walk Test or wheelchair endurance equivalent ¶
• Strength measures: ¶
o Lower-limb dynamometry or standardized manual muscle testing ¶
• Spasticity measures: ¶
o Modified Ashworth Scale (MAS) ¶
o Clonus (if present) ¶
• Clinician-rated disease severity: ¶
o Spastic Paraplegia Rating Scale (SPRS) ¶
• Session-level safety/comfort logs: ¶
o adverse events, skin checks, discomfort ratings, stimulation tolerability. ¶
2. Questionnaires ¶
• HSP-SNAP (symptom frequency/severity) ¶
• • NBSS and NBDS assessments for lower urinary tract and bowel function assessment ¶
• Falls history and mobility-confidence items if included in CRFs. ¶
3. Biomechanics / EMG ¶
• 3D kinematics/kinetics (joint angles, spatiotemporal gait parameters) for ambulatory participants ¶
• Equivalent seated mobility/movement kinematics for wheelchair users as applicable ¶
• Surface EMG from lower-limb muscles during tasks (timing, amplitude, co-contraction indices). ¶
4. Blood specimens ¶
• Metabolomic and lipid profiling outputs (de-identified) ¶
• Protein/biomarker assays including Big-tau (de-identified) ¶
• Any genetic-related research analyses performed on de-identified samples.

Research Description FutureUseAndSharing changed by vkpa225 on 1/16/2026 12:36:57 PM

The study team will retain the following coded (study-ID labeled) materials: ¶
• Blood-derived specimens: coded plasma/serum aliquots remaining after primary metabolomic/biomarker analyses. ¶
• De-identified/coded research data: motor and mobility outcomes (10MWT or wheelchair equivalent, 6MWT or equivalent, Sit-to-Stand test), spasticity measures (MAS), clinician ratings (SPRS), questionnaires (HSP-SNAP), NBSS and NBDS questionnaires, stimulation parameters/tolerability logs, and mechanistic datasets (3D gait/motion capture and surface EMG where applicable). ¶
¶
Direct identifiers (name, MRN, DOB, contact info) will not be kept with these research materials. ¶
¶
Stored coded specimens and de-identified data may be used for future studies aimed at: ¶
• Understanding biological mechanisms and biomarkers of HSP, including metabolomic, lipidomic, and protein biomarker work (e.g., Big-tau and related markers). ¶
• Exploring predictors of response to neuromodulation and refining personalized tSCS protocols for HSP. ¶
• Secondary analysis linking biological markers with motor, spasticity, and functional outcomes. ¶
• Sharing de-identified datasets to support replication, meta-analyses, and development of larger controlled trials in HSP. ¶
Future use will be limited to HSP/neuromotor/rehabilitation-relevant questions under IRB oversight. ¶
¶
Risks of additional use ¶
Risks of future use are primarily related to privacy: ¶
• Although identifiers will be removed, there is a small risk of loss of confidentiality or re-identification, especially for rare conditions. ¶
• If genetic or biomarker analyses are performed, results could theoretically reveal sensitive health information; however, no individual results will be returned for clinical use. ¶
No additional physical risk is expected because future research uses already-collected materials. ¶
¶
To protect privacy: ¶
• All retained materials will be labeled only with a study ID. ¶
• The code key linking IDs to identities will be stored separately on secure UK servers accessible only to the PI/designated staff. ¶
• Data and specimens will be stored in password-protected databases/locked freezers in UK-approved research space. ¶
• Any shared data/specimens will be de-identified or coded, and recipients will be prohibited from attempting re-identification. ¶
¶
Coded specimens and de-identified datasets may be stored and used for an indefinite period, consistent with the goal of enabling future HSP-related research and open-science data sharing. ¶
¶
Procedures for sharing with secondary researchers ¶
• De-identified datasets may be shared through public repositories or with qualified investigators after IRB approval, consistent with open-science plan. ¶
• Coded plasma aliquots may be shared with external collaborators only under an approved Material Transfer Agreement (MTA) and IRB authorization. One planned transfer is to Drexel University for Big-tau biomarker discovery using anonymized plasma. ¶
• Secondary researchers must agree to use materials only for approved aims, maintain secure storage, and not share onward without permission. ¶
¶
Withdrawal process and limitations ¶
Participants may request withdrawal of stored specimens/data at any time by contacting the Dr. Rahul Sachdeva, PI, in writing. ¶
• If materials are still coded and identifiable via the key, they will be destroyed and not used for future studies. ¶
• If materials/data have already been de-identified and shared or placed in a repository, withdrawal may not be possible because the research team can no longer link them to the participant. ¶
¶

This limitation will be clearly stated in the consent.
Research Description Payment changed by vkpa225 on 1/16/2026 2:46:20 PM
Participants may receive up to $725 to take part in this study. Participants will receive $100 for completing assessments conducted during the four study visits ~~(with or without stimulation sessions)~~ and $25 for each additional stimulation visit, as well as for the consent visit if the participant chooses to complete consent in person. If a participant withdraws early, compensation will be prorated, such that they will be paid for the study visits or portions of the study they have completed. ¶
¶
Participants will receive compensation in the form of a check/gift card. Payment will be provided upon completion of the study, or on a prorated basis if a participant withdraws early, and is expected to be distributed within 6-8 weeks of the study completion/withdrawal. ¶
¶
If participants choose to withdraw early, their payment will be prorated, meaning they will be paid for the visits or parts of the study that they completed. ¶
With a few exceptions, study payments are considered taxable income reportable to the Internal Revenue Service (IRS). A form 1099 will be sent to them if their total payments for research participation are $600 or more in a calendar year.
Research Description Payment changed by vkpa225 on 1/16/2026 12:35:05 PM
Participants ~~will receive $725 to take part in this study.~~ may receive $725 to take part in this study. Participants will receive $100 for completing assessments conducted during the four study visits (with or without stimulation sessions) and $25 for each additional stimulation visit, as well as for the consent visit if the participant chooses to complete consent in person. If a participant withdraws early, compensation will be prorated, such that they will be paid for the study visits or portions of the study they have completed. ¶
¶
Participants will receive compensation in the form of a check/gift card. Payment will be provided upon completion of the study, or on a prorated basis if a participant withdraws early, and is expected to be distributed within 6-8 weeks of the study completion/withdrawal. ¶
¶
If participants choose to withdraw early, their payment will be pro-rated, meaning they will be paid for the visits or parts of the study that they completed. ¶
With a few exceptions, study payments are considered taxable income reportable to the Internal Revenue Service (IRS). A form 1099 will be sent to them if their total payments for research participation are $600 or more in a calendar year.
Research Description ResearchMaterialsRecordsPrivacy changed by vkpa225 on 1/16/2026 12:35:05 PM
Research data (electronic): ¶
All identifiable research data (e.g., consent forms, screening logs, contact information) and coded study data (assessments, stimulation logs, questionnaires, EMG/gait files, lab results) will be stored on secure, password-protected University of Kentucky servers and/or UK-approved encrypted databases. Data will be labeled with a unique study ID; direct identifiers (name, MRN, DOB, contact info) will be stored separately from research datasets. Only authorized study personnel will have access, and access will be role-based. ¶
¶
Some study sessions and procedures may be videotaped and/or photographed for research and documentation purposes. When applicable, facial features and other identifying information will be blurred or otherwise de-identified to protect participant privacy, and all recordings will be stored securely with access limited to authorized study personnel. ¶
¶
Paper records: ¶
Any paper documents (e.g., signed consent forms, paper questionnaires) will be kept in locked filing cabinets within Dr. Sachdeva's PM&R research lab ~~at Cardinal Hill Rehabilitation Hospital (CHRH)~~, a secure UK-affiliated facility. ¶
¶
Biological specimens: ¶
Blood samples will be processed and stored as coded specimens labeled only with a study ID. Samples will be stored in secured, access-controlled freezers in Dr. Rahul Sachdeva's lab ~~at CHRH, Dr. Samaan's lab at the Human Performance laboratory~~, located at the Biomedical Biological Sciences Research Building (BBSRB), Dr. Samaan's freezer located in room 218 in the Seaton Center, and/or the CNS Metabolism COBRE Metabolomics Core as required for metabolomic assays. A master key linking study IDs to participant identities will be stored separately on a secure UK server accessible only to the PI and designated staff. ¶
Confidentiality protections: ¶
• Study IDs will be used on all data/specimens. ¶
• The linkage key will be stored separately and securely. ¶
• Electronic data will be encrypted in transit and at rest where supported. ¶
• Only minimum necessary PHI will be accessed for eligibility/safety. ¶
• Results will be reported in aggregate; no individual will be identifiable in publications or presentations. ¶
Access will be limited to personnel who need it to conduct the study: ¶
• PI and Co-Investigators (UK-affiliated): access to coded research data for oversight, safety, and analysis. ¶
• Research staff/trainees listed on IRB: access to coded data for scheduling, stimulation delivery, assessments, and data entry. ¶
• Metabolomics Core staff (UK-affiliated): access to coded plasma/serum samples only, to run approved assays. ¶
• Biostatistical consultant: access to de-identified or coded datasets for analysis. ¶
No one outside the approved study team will have access to identifiers. ¶
¶
Data/specimen management after study completion ¶
Retention: Study data and coded specimens will be retained per UK and sponsor/ORI retention policies (typically at least 6 years after study closeout), unless a longer period is required for regulatory reasons. ¶
De-identification for future use/sharing: After primary analyses are complete, datasets intended for sharing will have all direct identifiers removed and will be released only in de-identified form through public repositories, consistent with proposal's open-science plan. ¶
Specimens: Remaining coded specimens may be retained for future HSP-related biomarker work. Any future use will involve only coded/de-identified samples and will require IRB approval consistent with the consent's future-use language. If specimens are exhausted or no longer needed, they will be destroyed using UK-approved biohazard disposal procedures in compliance with institutional policy. ¶
¶
Sharing data/specimens with non-UK collaborators ¶
Consistent with the proposal, the study will share: ¶
1. De-identified study data through appropriate public repositories after completion of primary analyses. ¶
2. Anonymized/coded plasma aliquots with Drexel University for Big-tau biomarker discovery.
Research Description ResearchProcedures changed by vkpa225 on 1/16/2026 2:46:20 PM
Participants will take part in a structured, supervised research program at Cardinal Hill Rehabilitation Hospital (CHRH) – Physical Medicine & Rehabilitation (PM&R) Research Lab (The PM&R department and laboratory space is leased by the University of Kentucky and located within CHRH; however, CHRH itself will not be involved in the conduct of this study in any other capacity) and the Human Performance Laboratory/Biodynamics Lab at the UK Campus. After a baseline assessment at the Human Performance Laboratory at the UK Campus, participants will attend twice-weekly sessions of noninvasive transcutaneous spinal cord stimulation (tSCS) for 8 weeks at the PM&R Lab. Assessments at Week 1, 4, 8, and 16 will be conducted at the Human Performance Laboratory/Biodynamics lab at the UK Campus. Each stimulation visit will be monitored by trained research and clinical staff to ensure comfort and safety. Participants will receive stimulation while seated or (if able) during light mobility tasks. The study concludes with an end-of-treatment assessment and a single follow-up assessment visit 8 weeks later. ¶
¶
Participants will participate in a structured, supervised research program at the ~~C~~PM&R Lab and the Human Performance Laboratory/Biodynamics Lab on the UK Campus. After a baseline assessment at the Human Performance Laboratory at the UK Campus, participants will attend twice-weekly sessions of noninvasive transcutaneous spinal cord stimulation (tSCS) for 8 weeks at the PM&R Research Lab located in CHRH. Assessments at Week 1, 4, 8 and 16 will be conducted at the Human Performance Laboratory/Biodynamics Lab at the UK Campus. Each stimulation visit will be monitored by trained research and clinical staff to ensure comfort and safety. Participants will receive stimulation while seated or (if able) during light mobility tasks. The study concludes with an end-of-treatment assessment and a single follow-up assessment visit 8 weeks later. ¶
¶
Participants will be expected to: ¶
• Attend all scheduled lab visits over the 16-week study period. ¶
• Complete baseline, post-treatment, and follow-up assessments of motor function, mobility, strength, and spasticity. ¶
• Participate in 16 stimulation sessions over 8 weeks (2 sessions per week). ¶
• Complete questionnaires about symptoms and daily function. ¶
• Provide blood samples at four timepoints (Week 1, 4, 8, and 16). ¶
• Inform the study team about any changes in health, medications, or adverse events during the study. ¶
Testing and stimulation procedures will be adapted to each participant's mobility level, including people who walk with or without assistive devices and people who use wheelchairs. ¶
¶
Each participant will be in the study for about 16 weeks total: ¶
• The stimulation intervention will be administered over an 8-week (2-month) period. Each stimulation session will last up to 1 hour, for a total of approximately 16 hours of stimulation across the study. ¶
• 8 additional weeks without stimulation, ending with one follow-up assessment visit ¶
¶
Outline the schedule and timing of study procedures. ¶
A. Consent and Screening Visit (Week -1) ¶
On their first visit, the research team will review the consent form with participants, confirm eligibility for the study, and may conduct basic assessments related to walking or mobility (completed in the participant's usual manner, including the use of a mobility device or wheelchair, if applicable). ¶
If participants prefer, the consent process may be completed remotely by emailing the IRB-approved consent form in advance. In such cases, participants will be given adequate time to review the document, and any questions will be addressed by the Principal Investigator or study personnel via phone, email, or Zoom, according to the participant's preference. ¶
¶
B. Stimulation Period and assessments (Weeks 1-8) ¶
Before the study commences, clinicians and the study team will perform baseline assessments that may take up to 4 hours (only 4 sessions with the assessment throughout the study; Week 1, 4, 8, and 16), and may include: ¶
1. Walking or mobility tests (completed in participants' usual way, including with a mobility device or wheelchair if applicable) ¶
2. Muscle strength testing ¶
3. Spasticity evaluations ¶
4. Symptom questionnaires ¶
5. Collect the first blood sample at baseline assessment ¶
¶
Participants will attend two visits per week for 16 total stimulation sessions. Each session lasts about 1.5 hours. During each session: ¶
a. Electrodes will be placed on the skin over the lower back. ~~Electrode placement may differ slightly depending on each individual and the tSCS device that is used.~~ ¶
b. Participants will receive transcutaneous spinal cord stimulation (tSCS) at a comfortable, non-painful level. Participants may feel tingling or tapping sensations under the electrodes. ¶
c. Participants may be asked to perform simple movements, seated or standing, depending on their mobility abilities. ¶
d. Provide blood samples on first visit for stimulation during week 1, mid-study at week 4, and last visit for stimulation at week 8. ¶
e. At Week 4 and at the end of the 8-week stimulation period, participants will: repeat walking or mobility tests, strength and spasticity assessments, and complete questionnaires as described above during week 1. ¶
¶
C. Follow-Up Assessment Visit (Week 16) ¶
About 8 weeks after the last stimulation session, participants will return for a final follow-up visit. Participants will repeat the same assessments, walking or mobility tests, strength testing, spasticity measures, questionnaires, and provide the fourth (last) blood draw. This helps researchers learn whether any changes continued after stimulation ended. ¶
¶
Procedures to be carried out with each participant ¶
¶
1. Transcutaneous Spinal Cord Stimulation (tSCS) ¶
Transcutaneous Spinal Cord Stimulation (tSCS) will be delivered using a non-invasive spinal neuromodulation system (TESCoN or SCONE, SpineX Inc., Northridge, California, USA/ ARCEX (Onward Medical, USA). Two self-adhesive round electrodes placed at the midline over the lower thoracic (T11–T12) and/or upper lumbar vertebral levels (L1-L2) will serve as the cathodes, and two rectangular self-adhesive electrodes placed symmetrically over the iliac crests will serve as anodes. Electrode placement ~~may differ slightly depending~~will remain the same regardless o~~n~~f t~~SCS~~he device ~~that is~~ used. Stimulation will be delivered as high-frequency pulsed current using frequencies within a predefined range (typically 1-90 Hz; with a carrier frequency to make stimulation more comfortable), and session parameters will be adjusted per participant and per visit. Current amplitude will begin at ~10 mA and be increased in small steps to a comfortable, tolerable level below strong muscle contraction or until responses plateau. Skin at electrode sites will be inspected, cleaned, and prepared to reduce impedance before each session. We will use a conducting gel to protect the skin during stimulation. A skin-temperature probe will be positioned adjacent to the cathode to monitor local temperature and reduce the risk of skin irritation or burning. All stimulation sessions will be conducted at the UK PM&R Research Lab, except the 4 sessions coupled with mobility assessments that will be conducted in the Human Performance Laboratory/Biodynamics lab at the UK Campus under the supervision of trained study personnel, with real-time monitoring for discomfort, autonomic symptoms, or adverse events. Participants may complete seated, standing, or mobility tasks during stimulation, depending on their functional level, including those who ambulate with or without assistive devices and those who use wheelchairs. ¶
¶
2. Motor and Mobility Assessments ¶
Assessments will be adapted to participants' mobility level. Ambulatory participants will complete standard walking tests; wheelchair users will complete equivalent seated mobility tasks. ¶
a) 10-Meter Walk Test (or equivalent wheelchair speed test): 10MWT assesses walking speed over a 10-meter walkway at a comfortable and maximum safe pace. Timing occurs between 2 and 8 meters to exclude acceleration/deceleration, with two trials averaged per speed. Assistive devices are allowed. The 10MWT is quick, reliable, and sensitive to gait changes in HSP. Although no formal MCID exists for HSP, small gait speed changes reflect meaningful motor function improvements. ¶
b) 6-Minute Walk Test (or equivalent wheelchair endurance test): evaluates walking endurance by measuring the total distance walked over six minutes. Patients are instructed to walk as far as possible at their own pace, with rests allowed if needed. Distance measurements will be collected using an ActiGraph wearable activity monitor. The total distance reflects functional capacity and stamina, which are often affected in HSP due to progressive spasticity and weakness. ¶
c) Sit-to-stand test: Participants will be asked to perform a sit-to-stand task, whereby they will be told to stand up and sit down onto a stool at their own self-selected pace. ¶
d) Spastic Paraplegia Rating Scale (SPRS) clinician rating: SPRS is a reliable clinician-administered 13-item scale specific to HSP assessing gait, spasticity, strength, and sphincter function. Scores reflect disease severity and progression. ¶
e) HSP-SNAP patient-reported symptom questionnaire: HSP-SNAP is a patient-reported questionnaire rating frequency and severity of HSP symptoms such as stiffness, weakness, imbalance, and pain.55 It captures subjective symptom burden and treatment impact. ¶
f) 3D gait or movement analysis and surface EMG (when applicable): 3D Gait Analysis will be performed using a 14-camera motion capture system, wireless EMG system, and 3 ground force plates. We will track walking speed using timing gates. Wireless EMG sensors will be placed on the vastus lateralis, rectus femoris, and vastus medialis to assess muscle activation during the gait trials. The outcomes will include walking velocity, stance time, joint kinematics (hip, knee, and ankle) and joint kinetics. ¶
¶
All study participants will be asked to wear standardized sneakers during testing to reduce the impact of footwear on gait mechanics. Standardized sneakers will be provided ~~by the UK Biodynamics Lab~~. ¶
¶
All study participants will be asked to wear shorts and a tank top or sports bra for biomechanical testing. This attire will allow the research team to accurately visualize and palpate the anatomical landmarks needed to accurately track segment motion during biomechanical testing. ¶
¶
3. Strength and Spasticity Testing ¶
a) Muscle Strength: Bilateral isometric knee strength will be obtained with three, 5-sec maximal voluntary isometric contractions (MVIC) separated by one-minute rest. EMG is recorded concurrently for normalization with gait data. Knee pain during contractions is recorded on an 11-point visual analog scale. ¶
b) Modified Ashworth Scale (MAS): It's the standard clinical scale to rate muscle spasticity (tightness) in HSP. It measures muscle spasticity by assessing resistance to passive muscle stretch through the range of motion and grades the muscle tone on a 0-4 scale based on the resistance felt. ¶
MAS 0-4 scale: ¶
0: No increased muscle tone ¶
1: Slight increase in tone; catch and release at the end of the range ¶
1+: Slight increase; catch followed by minimal resistance through < half the range ¶
2: More marked increase through most of the range, but the limb still moves easily ¶
3: Considerable increase in tone; passive movement is difficult ¶
4: Limb is rigid in flexion or extension ¶
¶
4. Lower Urinary Tract (LUT) and Bowel Function: will be quantified as an exploratory outcome using NBSS and NBDS questionnaires. NBSS assesses urinary frequency, urgency, incontinence, and catheter use, while NBDS examines bowel movement frequency, constipation, and incontinence. ¶
¶
o Lower Urinary Tract (LUT) Assessments ¶
a) Neurogenic Bladder Symptom Score (NBSS): comprises 23 questions covering 3 domains, including incontinence, storage & voiding, and specific consequences, as well as one question on QoL. ¶
b) The Incontinence-QoL (I-QoL): comprises 10 questions covering 3 domains, including avoidance and limiting behaviour, psychosocial impacts, and social embarrassment, which will be summarised as a total score. All scores, for each domain and a total, will be transformed into a continuous scale value. ¶
o Bowel Dysfunction Assessments ¶
¶
a) Neurogenic bowel dysfunction score (NBD Score) The NBD score is a measure of both constipation and fecal incontinence and was developed for and validated in the SCI population This questionnaire comprises 10 questions focusing on defecation (i.e., frequency, duration, and clinical symptoms), constipation (i.e., use of aiding medication and digital stimulation), fecal incontinence (i.e., frequency, aiding medication, and flatus; and peri-anal skin problems. The consequential NBD score relates to four different neurogenic bowel dysfunction severity levels. ¶
5. Blood Collection and Metabolic/protein profiling ¶
Approximately 10 mL of venous blood samples will be collected at Weeks 1, 4, 8, and 16. Samples will be processed for metabolomic/lipid profiling and biomarker assays (including Big-tau). Targeted Metabolomic Profiling: Individuals with HSP also suffer from significant metabolic dysfunctions, notably in lipid metabolism. Many HSP genes encode proteins essential for lipid metabolism, with disturbances leading to impaired myelination, altered membrane trafficking, and axonal degeneration. Fasting plasma samples collected during the visits 2, 9, 17 and 18 will be analyzed for ~1100 lipid and polar metabolites across pathways including glycolysis, the tricarboxylic acid cycle, amino acid metabolism, and nucleotide biosynthesis [at our CNS Metabolism Center of Biomedical Research Excellence (COBRE) Metabolomics Core]. One plasma aliquot will be shipped to Drexel University for identification of Big-tau as a HSP biomarker (Dr. Emanuela Piermarini). Some analyses may involve genetic-related testing on de-identified samples. Individual research results will not be returned unless an important incidental finding is confirmed and IRB-approved. ¶
6. Statistical Analysis Plan ¶
Descriptive statistics will summarize feasibility (recruitment rate, retention, session adherence) and safety (adverse events). Efficacy outcomes will be analyzed using within-subject comparisons across timepoints (Week 1 vs Week 4 vs Week 8 vs Week 16). Depending on distribution, repeated-measures ANOVA or non-parametric Friedman tests will be used. Effect sizes (Cohen's d or Kendall's W) with 95% CIs will be reported to inform future powered trials. Missing data will be handled using mixed models or last-observation-carried-forward for sensitivity analyses. For Aim 1a, immediate effects of a single tSCS session will be assessed using paired t-tests. For Aim 1b, longitudinal changes across baseline, 8 weeks, and 16 weeks will be evaluated using linear mixed effects models to account for within-subject correlations and missing data. Primary and secondary outcomes (e.g., 10MWT, SPRS, MAS) will be treated as continuous variables. MAS, while ordinal, will be analyzed as continuous in the primary model with sensitivity analyses using nonparametric or ordinal methods. Post hoc comparisons will adjust for multiple testing. For Aim 2, which is exploratory and hypothesis-generating, univariate logistic regression will identify candidate predictors of motor response (defined as =0.1 m/s improvement in 10MWT or =1-point reduction in MAS). Candidate baseline predictors will include demographic and clinical variables such as age, sex, disease duration, genetic subtype, clinical phenotype (pure vs. complex), baseline gait speed, baseline MAS, lower-limb strength, and mobility status/assistive device use. Predictor-response associations will first be screened with univariate logistic regression. A multivariable logistic regression model will then be built using either backward stepwise selection guided by Akaike Information Criterion (AIC) or LASSO regularization, depending on sample size and collinearity. Model performance will be summarized by discrimination (AUROC) and calibration (Hosmer–Lemeshow test and calibration plots). Internal validation will be performed using 5-fold cross-validation and bootstrap resampling to assess stability and optimism. Finally, coefficients from the final model will be translated into a preliminary clinical scoring tool to stratify likelihood of response, acknowledging that external validation in a larger cohort will be required. Analyses will be conducted with a = 0.05, emphasizing estimation over significance testing. ¶
¶

This study does not involve deception. Participants will be fully informed about the purpose, procedures, risks, and voluntary nature of the study during the consent process. Therefore, no deception-related debriefing is required. ¶
¶
Standard/Routine Clinical Care (not part of the study) ¶
Participants will continue their usual clinical care for HSP throughout participation. Routine care may include, but is not limited to: ¶
• Ongoing neurology/rehabilitation follow-up visits ¶
• Physical therapy or occupational therapy prescribed as part of clinical care ¶
• Standard medications for spasticity or symptoms (see medication rules below) ¶
• Use of assistive devices such as braces, canes, walkers, or wheelchairs ¶
• Other clinically indicated evaluations or treatments determined by their treating physicians ¶
These routine care procedures are not required by the research study and will occur whether or not the individual participates. ¶
¶
Research-Specific Procedures (performed only for this project) ¶
All of the following are conducted specifically for research and are not standard care for HSP: ¶
1. tSCS sessions (16 sessions over 8 weeks). ¶
2. Research assessments in Weeks 1, 4, 8, and 16, including: ¶
• walking tests or equivalent wheelchair mobility tests ¶
• endurance testing (6-minute walk or equivalent) ¶
• lower-limb strength testing ¶
• spasticity evaluation (Modified Ashworth Scale) ¶
• clinician rating (Spastic Paraplegia Rating Scale; SPRS) ¶
• participant symptom questionnaires (HSP-SNAP) ¶
• questionnaires to assess bladder and bowel function ¶
• optional 3D gait/movement and surface EMG testing, when applicable ¶
3. Research blood draws (4 total) at Weeks 1, 4, 8, and 16 for metabolomics/biomarkers/genetic-related analyses on de-identified samples. ¶
4. Additional monitoring for adverse events related to stimulation and study testing. ¶
¶
Medication Rules During Participation ¶
Permitted Medications ¶
Participants may continue stable standard-of-care medications, including: ¶
• antispasticity agents such as baclofen, tizanidine, dantrolene, diazepam/clonazepam ¶
• botulinum toxin injections, if already part of routine care ¶
• medications for bladder symptoms, pain, sleep, mood, or other chronic conditions ¶
• vitamins/supplements, if stable ¶
Requirement: All neurologic/spasticity-related medications must be stable for at least 4 weeks before enrollment and participants are asked to avoid dose changes during the 8-week stimulation period, unless medically necessary. ¶
¶
Forbidden / Restricted Medications or Interventions ¶
Participants will be excluded or paused if they initiate or substantially change treatments that could confound outcomes or increase risk, including: ¶
• starting a new antispasticity medication or making major dose changes during the 8-week stimulation phase (unless required for safety/medical care) ¶
• starting or changing neuromodulation therapies, including any implanted or external electrical stimulation targeting the spine or lower limbs ¶
• enrolling in another interventional clinical trial that could affect mobility/spasticity during participation ¶
• new implantation of electrical medical devices (e.g., pacemakers, intrathecal pumps) during the study ¶
If a medically necessary medication change occurs, the research team will document it and determine whether the participant can safely continue, pause, or withdraw, depending on timing and magnitude of the change. ¶
¶
Visit-by-visit listing all procedures that will take place: See attached file
Research Description ResearchProcedures changed by vkpa225 on 1/16/2026 12:20:02 PM
Participants will take part in a structured, supervised research program at Cardinal Hill Rehabilitation Hospital (CHRH) – Physical Medicine & Rehabilitation (PM&R) Research Lab (The PM&R department and laboratory space is leased by the University of Kentucky and located within CHRH; however, CHRH itself will not be involved in the conduct of this study in any other capacity) and the Human Performance Laboratory/Biodynamics Lab at the UK Campus. After a baseline assessment at the Human Performance Laboratory at the UK Campus, participants will attend twice-weekly sessions of noninvasive transcutaneous spinal cord stimulation (tSCS) for 8 weeks at the PM&R Lab. Assessments at Week 1, 4, 8, and 16 will be conducted at the Human Performance Laboratory/Biodynamics lab at the UK Campus. Each stimulation visit will be monitored by trained research and clinical staff to ensure comfort and safety. Participants will receive stimulation while seated or (if able) during light mobility tasks. The study concludes with an end-of-treatment assessment and a single follow-up assessment visit 8 weeks later. ¶
¶
Participants will participate in a structured, supervised research program at the CPM&R Lab and the Human Performance Laboratory/Biodynamics Lab on the UK Campus. After a baseline assessment at the Human Performance Laboratory at the UK Campus, participants will attend twice-weekly sessions of noninvasive transcutaneous spinal cord stimulation (tSCS) for 8 weeks at the PM&R Research Lab located in CHRH. Assessments at Week 1, 4, 8 and 16 will be conducted at the Human Performance Laboratory/Biodynamics Lab at the UK Campus. Each stimulation visit will be monitored by trained research and clinical staff to ensure comfort and safety. Participants will receive stimulation while seated or (if able) during light mobility tasks. The study concludes with an end-of-treatment assessment and a single follow-up assessment visit 8 weeks later. ¶
¶
Participants will be expected to: ¶
• Attend all scheduled lab visits over the 16-week study period. ¶
• Complete baseline, post-treatment, and follow-up assessments of motor function, mobility, strength, and spasticity. ¶
• Participate in 16 stimulation sessions over 8 weeks (2 sessions per week). ¶
• Complete questionnaires about symptoms and daily function. ¶
• Provide blood samples at four timepoints (Week 1, 4, 8, and 16). ¶
• Inform the study team about any changes in health, medications, or adverse events during the study. ¶
Testing and stimulation procedures will be adapted to each participant's mobility level, including people who walk with or without assistive devices and people who use wheelchairs. ¶
¶
Each participant will be in the study for about 16 weeks total: ¶
• The stimulation intervention will be administered over an 8-week (2-month) period. Each stimulation session will last up to 1 hour, for a total of approximately 16 hours of stimulation across the study. ¶
• 8 additional weeks without stimulation, ending with one follow-up assessment visit ¶
¶
Outline the schedule and timing of study procedures. ¶
A. Consent and Screening Visit (Week -1) ¶
On their first visit, the research team will review the consent form with participants, confirm eligibility for the study, and may conduct basic assessments related to walking or mobility (completed in the participant's usual manner, including the use of a mobility device or wheelchair, if applicable). ¶
If participants prefer, the consent process may be completed remotely by emailing the IRB-approved consent form in advance. In such cases, participants will be given adequate time to review the document, and any questions will be addressed by the Principal Investigator or study personnel via phone, email, or Zoom, according to the participant's preference. ¶
¶
B. Stimulation Period and assessments (Weeks 1-8) ¶
Before the study commences, clinicians and the study team will perform baseline assessments that may take up to 4 hours (only 4 sessions with the assessment throughout the study; Week 1, 4, 8, and 16), and may include: ¶
1. Walking or mobility tests (completed in participants' usual way, including with a mobility device or wheelchair if applicable) ¶
2. Muscle strength testing ¶
3. Spasticity evaluations ¶
4. Symptom questionnaires ¶
5. Collect the first blood sample at baseline assessment ¶
¶
Participants will attend two visits per week for 16 total stimulation sessions. Each session lasts about 1.5 hours. During each session: ¶
a. Electrodes will be placed on the skin over the lower back. Electrode placement may differ slightly depending on each individual and the tSCS device that is used. ¶
b. Participants will receive transcutaneous spinal cord stimulation (tSCS) at a comfortable, non-painful level. Participants may feel tingling or tapping sensations under the electrodes. ¶
c. Participants may be asked to perform simple movements, seated or standing, depending on their mobility abilities. ¶
d. Provide blood samples on first visit for stimulation during week 1, mid-study at week 4, and last visit for stimulation at week 8. ¶
e. At Week 4 and at the end of the 8-week stimulation period, participants will: repeat walking or mobility tests, strength and spasticity assessments, and complete questionnaires as described above during week 1. ¶
¶
C. Follow-Up Assessment Visit (Week 16) ¶
About 8 weeks after the last stimulation session, participants will return for a final follow-up visit. Participants will repeat the same assessments, walking or mobility tests, strength testing, spasticity measures, questionnaires, and provide the fourth (last) blood draw. This helps researchers learn whether any changes continued after stimulation ended. ¶
¶
Procedures to be carried out with each participant ¶
¶
1. Transcutaneous Spinal Cord Stimulation (tSCS) ¶
Transcutaneous Spinal Cord Stimulation (tSCS) will be delivered using a non-invasive spinal neuromodulation system (TESCoN or SCONE, SpineX Inc., Northridge, California, USA/ ARCEX (Onward Medical, USA). Two self-adhesive round electrodes placed at the midline over the lower thoracic (T11–T12) and/or upper lumbar vertebral levels (L1-L2) will serve as the cathodes, and two rectangular self-adhesive electrodes placed symmetrically over the iliac crests will serve as anodes. Electrode placement may differ slightly depending on tSCS device that is used. Stimulation will be delivered as high-frequency pulsed current using frequencies within a predefined range (typically 1-90 Hz; with a carrier frequency to make stimulation more comfortable), and session parameters will be adjusted per participant and per visit. Current amplitude will begin at ~10 mA and be increased in small steps to a comfortable, tolerable level below strong muscle contraction or until responses plateau. Skin at electrode sites will be inspected, cleaned, and prepared to reduce impedance before each session. We will use a conducting gel to protect the skin during stimulation. A skin-temperature probe will be positioned adjacent to the cathode to monitor local temperature and reduce risk of skin irritation or burning. All stimulation sessions will be conducted at the UK PM&R Research Lab, except the 4 sessions coupled with mobility assessments that will be conducted in the Human Performance Laboratory/Biodynamics lab at the UK Campus under the supervision of trained study personnel, with real-time monitoring for discomfort, autonomic symptoms, or adverse events. Participants may complete seated, standing, or mobility tasks during stimulation, depending on their functional level, including those who ambulate with or without assistive devices and those who use wheelchairs. ¶
¶
2. Motor and Mobility Assessments ¶
Assessments will be adapted to participants' mobility level. Ambulatory participants will complete standard walking tests; wheelchair users will complete equivalent seated mobility tasks. ¶
a) 10-Meter Walk Test (or equivalent wheelchair speed test): 10MWT assesses walking speed over a 10-meter walkway at a comfortable and maximum safe pace. Timing occurs between 2 and 8 meters to exclude acceleration/deceleration, with two trials averaged per speed. Assistive devices are allowed. The 10MWT is quick, reliable, and sensitive to gait changes in HSP. Although no formal MCID exists for HSP, small gait speed changes reflect meaningful motor function improvements. ¶
b) 6-Minute Walk Test (or equivalent wheelchair endurance test): evaluates walking endurance by measuring the total distance walked over six minutes. Patients are instructed to walk as far as possible at their own pace, with rests allowed if needed. Distance measurements will be collected using an ActiGraph wearable activity monitor. The total distance reflects functional capacity and stamina, which are often affected in HSP due to progressive spasticity and weakness. ¶
c) Sit-to-stand test: Participants will be asked to perform a sit-to-stand task, whereby they will be told to stand up and sit down onto a stool at their own self-selected pace. ¶
d) Spastic Paraplegia Rating Scale (SPRS) clinician rating: SPRS is a reliable clinician-administered 13-item scale specific to HSP assessing gait, spasticity, strength, and sphincter function. Scores reflect disease severity and progression. ¶
e) HSP-SNAP patient-reported symptom questionnaire: HSP-SNAP is a patient-reported questionnaire rating frequency and severity of HSP symptoms such as stiffness, weakness, imbalance, and pain.55 It captures subjective symptom burden and treatment impact. ¶
f) 3D gait or movement analysis and surface EMG (when applicable): 3D Gait Analysis will be performed using a 14-camera motion capture system, wireless EMG system, and 3 ground force plates. We will track walking speed using timing gates. Wireless EMG sensors will be placed on the vastus lateralis, rectus femoris, and vastus medialis to assess muscle activation during the gait trials. The outcomes will include walking velocity, stance time, joint kinematics (hip, knee and ankle) and joint kinetics. ¶
¶
All study participants will be asked to wear standardized sneakers during testing to reduce the impact of footwear on gait mechanics. Standardized sneakers will be provided by the UK Biodynamics Lab. ¶
¶
All study participants will be asked to wear shorts and a tank top or sports bra for biomechanical testing. This attire will allow the research team to accurately visualize and palpate the anatomical landmarks needed to accurately track segment motion during biomechanical testing. ¶
¶
3. Strength and Spasticity Testing ¶
a) Muscle Strength: Bilateral isometric knee strength will be obtained with three, 5-sec maximal voluntary isometric contractions (MVIC) separated by one-minute rest. EMG is recorded concurrently for normalization with gait data. Knee pain during contractions is recorded on an 11-point visual analog scale. ¶
b) Modified Ashworth Scale (MAS): It's the standard clinical scale to rate muscle spasticity (tightness) in HSP. It measures muscle spasticity by assessing resistance to passive muscle stretch through the range of motion and grades the muscle tone on a 0-4 scale based on the resistance felt. ¶
MAS 0-4 scale: ¶
0: No increased muscle tone ¶
1: Slight increase in tone; catch and release at the end of the range ¶
1+: Slight increase; catch followed by minimal resistance through < half the range ¶
2: More marked increase through most of the range, but the limb still moves easily ¶
3: Considerable increase in tone; passive movement is difficult ¶
4: Limb is rigid in flexion or extension ¶
¶
4. Lower Urinary Tract (LUT) and Bowel Function: will be quantified as an exploratory outcome using NBSS and NBDS questionnaires. NBSS assesses urinary frequency, urgency, incontinence, and catheter use, while NBDS examines bowel movement frequency, constipation, and incontinence. ¶
¶
o Lower Urinary Tract (LUT) Assessments ¶
a) Neurogenic Bladder Symptom Score (NBSS): comprises 23 questions covering 3 domains, including incontinence, storage & voiding, and specific consequences, as well as one question on QoL. ¶
b) The Incontinence-QoL (I-QoL): comprises 10 questions covering 3 domains, including avoidance and limiting behaviour, psychosocial impacts, and social embarrassment, which will be summarised as a total score. All scores, for each domain and a total, will be transformed into a continuous scale value. ¶
o Bowel Dysfunction Assessments ¶
¶
a) Neurogenic bowel dysfunction score (NBD Score) The NBD score is a measure of both constipation and fecal incontinence and was developed for and validated in the SCI population This questionnaire comprises 10 questions focusing on defecation (i.e., frequency, duration, and clinical symptoms), constipation (i.e., use of aiding medication and digital stimulation), fecal incontinence (i.e., frequency, aiding medication, and flatus; and peri-anal skin problems. The consequential NBD score relates to four different neurogenic bowel dysfunction severity levels. ¶
5. Blood Collection and Metabolic/protein profiling ¶
Approximately 10 mL of venous blood samples will be collected at Weeks 1, 4, 8, and 16. Samples will be processed for metabolomic/lipid profiling and biomarker assays (including Big-tau). Targeted Metabolomic Profiling: Individuals with HSP also suffer from significant metabolic dysfunctions, notably in lipid metabolism. Many HSP genes encode proteins essential for lipid metabolism, with disturbances leading to impaired myelination, altered membrane trafficking, and axonal degeneration. Fasting plasma samples collected during the visits 2, 9, 17 and 18 will be analyzed for ~1100 lipid and polar metabolites across pathways including glycolysis, the tricarboxylic acid cycle, amino acid metabolism, and nucleotide biosynthesis [at our CNS Metabolism Center of Biomedical Research Excellence (COBRE) Metabolomics Core]. One plasma aliquot will be shipped to Drexel University for identification of Big-tau as a HSP biomarker (Dr. Emanuela Piermarini). Some analyses may involve genetic-related testing on de-identified samples. Individual research results will not be returned unless an important incidental finding is confirmed and IRB-approved. ¶
6. Statistical Analysis Plan ¶
Descriptive statistics will summarize feasibility (recruitment rate, retention, session adherence) and safety (adverse events). Efficacy outcomes will be analyzed using within-subject comparisons across timepoints (Week 1 vs Week 4 vs Week 8 vs Week 16). Depending on distribution, repeated-measures ANOVA or non-parametric Friedman tests will be used. Effect sizes (Cohen's d or Kendall's W) with 95% CIs will be reported to inform future powered trials. Missing data will be handled using mixed models or last-observation-carried-forward for sensitivity analyses. For Aim 1a, immediate effects of a single tSCS session will be assessed using paired t-tests. For Aim 1b, longitudinal changes across baseline, 8 weeks, and 16 weeks will be evaluated using linear mixed effects models to account for within-subject correlations and missing data. Primary and secondary outcomes (e.g., 10MWT, SPRS, MAS) will be treated as continuous variables. MAS, while ordinal, will be analyzed as continuous in the primary model with sensitivity analyses using nonparametric or ordinal methods. Post hoc comparisons will adjust for multiple testing. For Aim 2, which is exploratory and hypothesis-generating, univariate logistic regression will identify candidate predictors of motor response (defined as =0.1 m/s improvement in 10MWT or =1-point reduction in MAS). Candidate baseline predictors will include demographic and clinical variables such as age, sex, disease duration, genetic subtype, clinical phenotype (pure vs. complex), baseline gait speed, baseline MAS, lower-limb strength, and mobility status/assistive device use. Predictor-response associations will first be screened with univariate logistic regression. A multivariable logistic regression model will then be built using either backward stepwise selection guided by Akaike Information Criterion (AIC) or LASSO regularization, depending on sample size and collinearity. Model performance will be summarized by discrimination (AUROC) and calibration (Hosmer–Lemeshow test and calibration plots). Internal validation will be performed using 5-fold cross-validation and bootstrap resampling to assess stability and optimism. Finally, coefficients from the final model will be translated into a preliminary clinical scoring tool to stratify likelihood of response, acknowledging that external validation in a larger cohort will be required. Analyses will be conducted with a = 0.05, emphasizing estimation over significance testing. ¶
¶
This study does not involve deception. Participants will be fully informed about the purpose, procedures, risks, and voluntary nature of the study during the consent process. Therefore, no deception-related debriefing is required. ¶
¶
Standard/Routine Clinical Care (not part of the study) ¶
Participants will continue their usual clinical care for HSP throughout participation. Routine care may include, but is not limited to: ¶
• ~~o~~Ongoing neurology/rehabilitation follow-up visits ¶
• ~~p~~Physical therapy or occupational therapy prescribed as part of clinical care ¶
• ~~s~~Standard medications for spasticity or symptoms (see medication rules below) ¶
• ~~u~~Use of assistive devices such as braces, canes, walkers, or wheelchairs ¶
• ~~o~~Other clinically indicated evaluations or treatments determined by their treating physicians ¶
These routine care procedures are not required by the research study and will occur whether or not the individual participates. ¶

¶
Research-Specific Procedures (performed only for this project) ¶
All of the following are conducted specifically for research and are not standard care for HSP: ¶
1. tSCS sessions (16 sessions over 8 weeks). ¶
2. Research assessments in Weeks 1, 4, 8, and 16, including: ¶
• walking tests or equivalent wheelchair mobility tests ¶
• endurance testing (6-minute walk or equivalent) ¶
• lower-limb strength testing ¶
• spasticity evaluation (Modified Ashworth Scale) ¶
• clinician rating (Spastic Paraplegia Rating Scale; SPRS) ¶
• participant symptom questionnaires (HSP-SNAP) ¶
• questionnaires to assess bladder and bowel function ¶
• optional 3D gait/movement and surface EMG testing, when applicable ¶
3. Research blood draws (4 total) at Weeks 1, 4, 8, and 16 for metabolomics/biomarkers/genetic-related analyses on de-identified samples. ¶
4. Additional monitoring for adverse events related to stimulation and study testing. ¶
¶
Medication Rules During Participation ¶
Permitted Medications ¶
Participants may continue stable standard-of-care medications, including: ¶
• antispasticity agents such as baclofen, tizanidine, dantrolene, diazepam/clonazepam ¶
• botulinum toxin injections, if already part of routine care ¶
• medications for bladder symptoms, pain, sleep, mood, or other chronic conditions ¶
• vitamins/supplements, if stable ¶
Requirement: All neurologic/spasticity-related medications must be stable for at least 4 weeks before enrollment and participants are asked to avoid dose changes during the 8-week stimulation period, unless medically necessary. ¶
¶
Forbidden / Restricted Medications or Interventions ¶
Participants will be excluded or paused if they initiate or substantially change treatments that could confound outcomes or increase risk, including: ¶
• starting a new antispasticity medication or making major dose changes during the 8-week stimulation phase (unless required for safety/medical care) ¶
• starting or changing neuromodulation therapies, including any implanted or external electrical stimulation targeting the spine or lower limbs ¶
• enrolling in another interventional clinical trial that could affect mobility/spasticity during participation ¶
• new implantation of electrical medical devices (e.g., pacemakers, intrathecal pumps) during the study ¶
If a medically necessary medication change occurs, the research team will document it and determine whether the participant can safely continue, pause, or withdraw, depending on timing and magnitude of the change. ¶
¶
Visit-by-visit listing all procedures that will take place: See attached file
Research Description ResearchProcedures changed by vkpa225 on 1/16/2026 12:12:15 PM
Participants will take part in a structured, supervised research program at Cardinal Hill Rehabilitation Hospital (CHRH) – Physical Medicine & Rehabilitation (PM&R) Research Lab (The PM&R department and laboratory space is leased by the University of Kentucky and located within CHRH; however, CHRH itself will not be involved in the conduct of this study in any other capacity) and the Human Performance Laboratory/Biodynamics Lab at the UK Campus. After a baseline assessment at the Human Performance Laboratory at the UK Campus, participants will attend twice-weekly sessions of noninvasive transcutaneous spinal cord stimulation (tSCS) for 8 weeks at the PM&R Lab. Assessments at Week 1, 4, 8, and 16 will be conducted at the Human Performance Laboratory/Biodynamics lab at the UK Campus. Each stimulation visit will be monitored by trained research and clinical staff to ensure comfort and safety. Participants will receive stimulation while seated or (if able) during light mobility tasks. The study concludes with an end-of-treatment assessment and a single follow-up assessment visit 8 weeks later. ¶
¶
Participants will participate in a structured, supervised research program at the CPM&R Lab and the Human Performance Laboratory/Biodynamics Lab on the UK Campus. After a baseline assessment at the Human Performance Laboratory at the UK Campus, participants will attend twice-weekly sessions of noninvasive transcutaneous spinal cord stimulation (tSCS) for 8 weeks at the PM&R Research Lab located in CHRH. Assessments at Week 1, 4, 8 and 16 will be conducted at the Human Performance Laboratory/Biodynamics Lab at the UK Campus. Each stimulation visit will be monitored by trained research and clinical staff to ensure comfort and safety. Participants will receive stimulation while seated or (if able) during light mobility tasks. The study concludes with an end-of-treatment assessment and a single follow-up assessment visit 8 weeks later. ¶
¶
Participants will be expected to: ¶
• Attend all scheduled lab visits over the 16-week study period. ¶
• Complete baseline, post-treatment, and follow-up assessments of motor function, mobility, strength, and spasticity. ¶
• Participate in 16 stimulation sessions over 8 weeks (2 sessions per week). ¶
• Complete questionnaires about symptoms and daily function. ¶
• Provide blood samples at four timepoints (Week 1, 4, 8, and 16). ¶
• Inform the study team about any changes in health, medications, or adverse events during the study. ¶
Testing and stimulation procedures will be adapted to each participant's mobility level, including people who walk with or without assistive devices and people who use wheelchairs. ¶
¶
Each participant will be in the study for about 16 weeks total: ¶
• The stimulation intervention will be administered over an 8-week (2-month) period. Each stimulation session will last up to 1 hour, for a total of approximately 16 hours of stimulation across the study. ¶
• 8 additional weeks without stimulation, ending with one follow-up assessment visit ¶
¶
Outline the schedule and timing of study procedures. ¶
A. Consent and Screening Visit (Week -1) ¶
On their first visit, the research team will review the consent form with participants, confirm eligibility for the study, and may conduct basic assessments related to walking or mobility (completed in the participant's usual manner, including the use of a mobility device or wheelchair, if applicable). ¶
If participants prefer, the consent process may be completed remotely by emailing the IRB-approved consent form in advance. In such cases, participants will be given adequate time to review the document, and any questions will be addressed by the Principal Investigator or study personnel via phone, email, or Zoom, according to the participant's preference. ¶
¶
B. Stimulation Period and assessments (Weeks 1-8) ¶
Before the study commences, clinicians and the study team will perform baseline assessments that may take up to 4 hours (only 4 sessions with the assessment throughout the study; Week 1, 4, 8, and 16), and may include: ¶
1. Walking or mobility tests (completed in participants' usual way, including with a mobility device or wheelchair if applicable) ¶
2. Muscle strength testing ¶
3. Spasticity evaluations ¶
4. Symptom questionnaires ¶
5. Collect the first blood sample at baseline assessment ¶
¶
Participants will attend two visits per week for 16 total stimulation sessions. Each session lasts about 1.5 hours. During each session: ¶
a. Electrodes will be placed on the skin over the lower back. Electrode placement may differ slightly depending on each individual and the tSCS device that is used. ¶
b. Participants will receive transcutaneous spinal cord stimulation (tSCS) at a comfortable, non-painful level. Participants may feel tingling or tapping sensations under the electrodes. ¶
c. Participants may be asked to perform simple movements, seated or standing, depending on their mobility abilities. ¶
d. Provide blood samples on first visit for stimulation during week 1, mid-study at week 4, and last visit for stimulation at week 8. ¶
e. At Week 4 and at the end of the 8-week stimulation period, participants will: repeat walking or mobility tests, strength and spasticity assessments, and complete questionnaires as described above during week 1. ¶
¶
C. Follow-Up Assessment Visit (Week 16) ¶
About 8 weeks after the last stimulation session, participants will return for a final follow-up visit. Participants will repeat the same assessments, walking or mobility tests, strength testing, spasticity measures, questionnaires, and provide the fourth (last) blood draw. This helps researchers learn whether any changes continued after stimulation ended. ¶
¶
Procedures to be carried out with each participant ¶
¶
1. Transcutaneous Spinal Cord Stimulation (tSCS) ¶
Transcutaneous Spinal Cord Stimulation (tSCS) will be delivered using a non-invasive spinal neuromodulation system (TESCoN or SCONE, SpineX Inc., Northridge, California, USA/ ARCEX (Onward Medical, USA). Two self-adhesive round electrodes placed at the midline over the lower thoracic (T11–T12) and/or upper lumbar vertebral levels (L1-L2) will serve as the cathodes, and two rectangular self-adhesive electrodes placed symmetrically over the iliac crests will serve as anodes. Electrode placement may differ slightly depending on tSCS device that is used. Stimulation will be delivered as high-frequency pulsed current using frequencies within a predefined range (typically 1–-90 Hz; with a carrier frequency to make stimulation more comfortable), and session parameters will be adjusted per participant and per visit. Current amplitude will begin at ~10 mA and be increased in small steps to a comfortable, tolerable level below strong muscle contraction or until responses plateau. Skin at electrode sites will be inspected, cleaned, and prepared to reduce impedance before each session. We will use a conducting gel to protect the skin during stimulation. A skin-temperature probe will be positioned adjacent to the cathode to monitor local temperature and reduce risk of skin irritation or burning. All stimulation sessions will be conducted at the UK PM&R Research Lab, except the 4 sessions coupled with mobility assessments that will be conducted in the Human Performance Laboratory/Biodynamics lab at the UK Campus under the supervision of trained study personnel, with real-time monitoring for discomfort, autonomic symptoms, or adverse events. Participants may complete seated, standing, or mobility tasks during stimulation, depending on their functional level, including those who ambulate with or without assistive devices and those who use wheelchairs. ¶
¶
2. Motor and Mobility Assessments ¶
Assessments will be adapted to participants' mobility level. Ambulatory participants will complete standard walking tests; wheelchair users will complete equivalent seated mobility tasks. ¶
a) 10-Meter Walk Test (or equivalent wheelchair speed test): 10MWT assesses walking speed over a 10-meter walkway at a comfortable and maximum safe pace. 53 Timing occurs between 2 and 8 meters to exclude acceleration/deceleration, with two trials averaged per speed. Assistive devices are allowed. The 10MWT is quick, reliable, and sensitive to gait changes in HSP. Although no formal MCID exists for HSP, small gait speed changes reflect meaningful motor function improvements. ¶
b) 6-Minute Walk Test (or equivalent wheelchair endurance test): evaluates walking endurance by measuring the total distance walked over six minutes.53 Patients are instructed to walk as far as possible at their own pace, with rests allowed if needed. Distance measurements will be collected using an ActiGraph wearable activity monitor. The total distance reflects functional capacity and stamina, which are often affected in HSP due to progressive spasticity and weakness. ¶
c) Sit-to-stand test: Participants will be asked to perform a sit-to-stand task, whereby they will be told to stand up and sit down onto a stool at their own self-selected pace. ¶
d) Spastic Paraplegia Rating Scale (SPRS) clinician rating: SPRS is a reliable clinician-administered 13-item scale specific to HSP assessing gait, spasticity, strength, and sphincter function. Scores reflect disease severity and progression.54 ¶
de) HSP-SNAP patient-reported symptom questionnaire: HSP-SNAP is a patient-reported questionnaire rating frequency and severity of HSP symptoms such as stiffness, weakness, imbalance, and pain.55 It captures subjective symptom burden and treatment impact. ¶
ef) 3D gait or movement analysis and surface EMG (when applicable): 3D Gait Analysis will be performed using a 14-camera motion capture system, wireless EMG system, and 3 ground force plates.56,57 We will track walking speed using timing gates. Wireless EMG sensors will be placed on the vastus lateralis, rectus femoris, and vastus medialis to assess muscle activation during the gait trials. The outcomes will include walking velocity, stance time, joint kinematics (hip, knee and ankle) and joint kinetics. ¶
¶
All study participants will be asked to wear standardized sneakers during testing to reduce the impact of footwear on gait mechanics. Standardized sneakers will be provided by the UK Biodynamics Lab. ¶
¶
All study participants will be asked to wear shorts and a tank top or sports bra for biomechanical testing. This attire will allow the research team to accurately visualize and palpate the anatomical landmarks needed to accurately track segment motion during biomechanical testing. ¶
¶
3. Strength and Spasticity Testing ¶
a) Muscle Strength: Bilateral isometric knee strength will be obtained with three, 5-sec maximal voluntary isometric contractions (MVIC) separated by one-minute rest.56 EMG is recorded concurrently for normalization with gait data. Knee pain during contractions is recorded on an 11-point visual analog scale. ¶
b) Modified Ashworth Scale (MAS): It's the standard clinical scale to rate muscle spasticity (tightness) in HSP. It measures muscle spasticity by assessing resistance to passive muscle stretch through the range of motion and grades the muscle tone on a 0-4 scale based on the resistance felt.51 ¶
MAS 0-4 scale: ¶
0: No increased muscle tone ¶
1: Slight increase in tone; catch and release at the end of the range ¶
1+: Slight increase; catch followed by minimal resistance through < half the range ¶
2: More marked increase through most of the range, but the limb still moves easily ¶
3: Considerable increase in tone; passive movement is difficult ¶
4: Limb is rigid in flexion or extension ¶
¶
4. Lower Urinary Tract (LUT) and Bowel Function: will be quantified as an exploratory outcome using NBSS and NBDS questionnaires. NBSS assesses urinary frequency, urgency, incontinence, and catheter use,58 while NBDS examines bowel movement frequency, constipation, and incontinence.59 ¶
¶
o Lower Urinary Tract (LUT) Assessments ¶
a) Neurogenic Bladder Symptom Score (NBSS): comprises 23 questions covering 3 domains, including incontinence, storage & voiding, and specific consequences, as well as one question on QoL. ¶
b) The Incontinence-QoL (I-QoL): comprises 10 questions covering 3 domains, including avoidance and limiting behaviour, psychosocial impacts, and social embarrassment, which will be summarised as a total score. All scores, for each domain and a total, will be transformed into a continuous scale value. ¶
o Bowel Dysfunction Assessments ¶
¶
a) Neurogenic bowel dysfunction score (NBD Score) The NBD score is a measure of both constipation and fecal incontinence and was developed for and validated in the SCI population This questionnaire comprises 10 questions focusing on defecation (i.e., frequency, duration, and clinical symptoms), constipation (i.e., use of aiding medication and digital stimulation), fecal incontinence (i.e., frequency, aiding medication, and flatus; and peri-anal skin problems. The consequential NBD score relates to four different neurogenic bowel dysfunction severity levels. ¶
5. Blood Collection and Metabolic/protein profiling ¶
Approximately 10 mL of venous blood samples will be collected at Weeks 1, 4, 8, and 16. Samples will be processed for metabolomic/lipid profiling and biomarker assays (including Big-tau). Targeted Metabolomic Profiling: Individuals with HSP also suffer withfrom significant metabolic dysfunctions, notably in lipid metabolism.60 Many HSP genes encode proteins essential for lipid metabolism, with disturbances leading to impaired myelination, altered membrane trafficking, and axonal degeneration. Fasting plasma samples collected during the visits 2, 9, 17 and 18 will be analyzed for ~1100 lipid and polar metabolites across pathways including glycolysis, the tricarboxylic acid cycle, amino acid metabolism, and nucleotide biosynthesis [at our CNS Metabolism Center of Biomedical Research Excellence (COBRE) Metabolomics Core]. One plasma aliquot will be shipped to Drexel University for identification of Big-tau as a HSP biomarker (Dr. Emanuela Piermarini). Some analyses may involve genetic-related testing on de-identified samples. Individual research results will not be returned unless an important incidental finding is confirmed and IRB-approved. ¶
6. Statistical Analysis Plan ¶
Descriptive statistics will summarize feasibility (recruitment rate, retention, session adherence) and safety (adverse events). Efficacy outcomes will be analyzed using within-subject comparisons across timepoints (Week 1 vs Week 4 vs Week 8 vs Week 16). Depending on distribution, repeated-measures ANOVA or non-parametric Friedman tests will be used. Effect sizes (Cohen's d or Kendall's W) with 95% CIs will be reported to inform future powered trials. Missing data will be handled using mixed models or last-observation-carried-forward for sensitivity analyses. For Aim 1a, immediate effects of a single tSCS session will be assessed using paired t-tests. For Aim 1b, longitudinal changes across baseline, 8 weeks, and 16 weeks will be evaluated using linear mixed effects models to account for within-subject correlations and missing data. Primary and secondary outcomes (e.g., 10MWT, SPRS, MAS) will be treated as continuous variables. MAS, while ordinal, will be analyzed as continuous in the primary model with sensitivity analyses using nonparametric or ordinal methods. Post hoc comparisons will adjust for multiple testing. For Aim 2, which is exploratory and hypothesis-generating, univariate logistic regression will identify candidate predictors of motor response (defined as =0.1 m/s improvement in 10MWT or =1-point reduction in MAS). Candidate baseline predictors will include demographic and clinical variables such as age, sex, disease duration, genetic subtype, clinical phenotype (pure vs. complex), baseline gait speed, baseline MAS, lower-limb strength, and mobility status/assistive device use. Predictor-response associations will first be screened with univariate logistic regression. A multivariable logistic regression model will then be built using either backward stepwise selection guided by Akaike Information Criterion (AIC) or LASSO regularization, depending on sample size and collinearity. Model performance will be summarized by discrimination (AUROC) and calibration (Hosmer–Lemeshow test and calibration plots). Internal validation will be performed using 5-fold cross-validation and bootstrap resampling to assess stability and optimism. Finally, coefficients from the final model will be translated into a preliminary clinical scoring tool to stratify likelihood of response, acknowledging that external validation in a larger cohort will be required. Analyses will be conducted with a = 0.05, emphasizing estimation over significance testing. ¶
¶
This study does not involve deception. Participants will be fully informed about the purpose, procedures, risks, and voluntary nature of the study during the consent process. Therefore, no deception-related debriefing is required. ¶
¶
Standard/Routine Clinical Care (not part of the study) ¶
Participants will continue their usual clinical care for HSP throughout participation. Routine care may include, but is not limited to: ¶
• ongoing neurology/rehabilitation follow-up visits ¶
• physical therapy or occupational therapy prescribed as part of clinical care ¶
• standard medications for spasticity or symptoms (see medication rules below) ¶
• use of assistive devices such as braces, canes, walkers, or wheelchairs ¶
• other clinically indicated evaluations or treatments determined by their treating physicians ¶
These routine care procedures are not required by the research study and will occur whether or not the individual participates. ¶
¶
Research-Specific Procedures (performed only for this project) ¶
All of the following are conducted specifically for research and are not standard care for HSP: ¶
1. tSCS sessions (16 sessions over 8 weeks). ¶
2. Research assessments in Weeks 1, 4, 8, and 16, including: ¶
• walking tests or equivalent wheelchair mobility tests ¶
• endurance testing (6-minute walk or equivalent) ¶
• lower-limb strength testing ¶
• spasticity evaluation (Modified Ashworth Scale) ¶
• clinician rating (Spastic Paraplegia Rating Scale; SPRS) ¶

• participant symptom questionnaires (HSP-SNAP) ¶
• questionnaires to assess bladder and bowel function ¶
• optional 3D gait/movement and surface EMG testing, when applicable ¶
3. Research blood draws (4 total) at Weeks 1, 4, 8, and 16 for metabolomics/biomarkers/genetic-related analyses on de-identified samples. ¶
4. Additional monitoring for adverse events related to stimulation and study testing. ¶
¶
Medication Rules During Participation ¶
Permitted Medications ¶
Participants may continue stable standard-of-care medications, including: ¶
• antispasticity agents such as baclofen, tizanidine, dantrolene, diazepam/clonazepam ¶
• botulinum toxin injections, if already part of routine care ¶
• medications for bladder symptoms, pain, sleep, mood, or other chronic conditions ¶
• vitamins/supplements, if stable ¶
Requirement: All neurologic/spasticity-related medications must be stable for at least 4 weeks before enrollment and participants are asked to avoid dose changes during the 8-week stimulation period, unless medically necessary. ¶
¶
Forbidden / Restricted Medications or Interventions ¶
Participants will be excluded or paused if they initiate or substantially change treatments that could confound outcomes or increase risk, including: ¶
• starting a new antispasticity medication or making major dose changes during the 8-week stimulation phase (unless required for safety/medical care) ¶
• starting or changing neuromodulation therapies, including any implanted or external electrical stimulation targeting the spine or lower limbs ¶
• enrolling in another interventional clinical trial that could affect mobility/spasticity during participation ¶
• new implantation of electrical medical devices (e.g., pacemakers, intrathecal pumps) during the study ¶
If a medically necessary medication change occurs, the research team will document it and determine whether the participant can safely continue, pause, or withdraw, depending on timing and magnitude of the change. ¶
¶
Visit-by-visit listing all procedures that will take place: See attached file

Research Description ResearchProcedures changed by vkpa225 on 1/16/2026 12:06:22 PM

Participants will take part in a structured, supervised research program at Cardinal Hill Rehabilitation Hospital (CHRH) – Physical Medicine & Rehabilitation (PM&R) Research Lab (The PM&R department and laboratory space is leased by the University of Kentucky and located within CHRH; however, CHRH itself will not be involved in the conduct of this study in any other capacity) and the Human Performance Laboratory/Biodynamics Lab at the UK Campus. After a baseline assessment at the Human Performance Laboratory at the UK Campus, participants will attend twice-weekly sessions of noninvasive transcutaneous spinal cord stimulation (tSCS) for 8 weeks at the PM&R Lab. Assessments at Week 1, 4, 8, and 16 will be conducted at the Human Performance Laboratory/Biodynamics lab at the UK Campus. Each stimulation visit will be monitored by trained research and clinical staff to ensure comfort and safety. Participants will receive stimulation while seated or (if able) during light mobility tasks. The study concludes with an end-of-treatment assessment and a single follow-up assessment visit 8 weeks later. ¶
¶
Participants will participate in a structured, supervised research program at the CPM&R Lab and the Human Performance Laboratory/Biodynamics Lab on the UK Campus. After a baseline assessment at the Human Performance Laboratory at the UK Campus, participants will attend twice-weekly sessions of noninvasive transcutaneous spinal cord stimulation (tSCS) for 8 weeks at the PM&R Research Lab located in CHRH. Assessments at Week 1, 4, 8 and 16 will be conducted at the Human Performance Laboratory/Biodynamics Lab at the UK Campus. Each stimulation visit will be monitored by trained research and clinical staff to ensure comfort and safety. Participants will receive stimulation while seated or (if able) during light mobility tasks. The study concludes with an end-of-treatment assessment and a single follow-up assessment visit 8 weeks later. ¶
¶
Participants will be expected to: ¶
• Attend all scheduled lab visits over the 16-week study period. ¶
• Complete baseline, post-treatment, and follow-up assessments of motor function, mobility, strength, and spasticity. ¶
• Participate in 16 stimulation sessions over 8 weeks (2 sessions per week). ¶
• Complete questionnaires about symptoms and daily function. ¶
• Provide blood samples at four timepoints (Week 1, 4, 8, and 16). ¶
• Inform the study team about any changes in health, medications, or adverse events during the study. ¶
Testing and stimulation procedures will be adapted to each participant's mobility level, including people who walk with or without assistive devices and people who use wheelchairs. ¶
¶
Each participant will be in the study for about 16 weeks total: ¶
• The stimulation intervention will be administered over an 8-week (2-month) period. Each stimulation session will last up to 1 hour, for a total of approximately 16 hours of stimulation across the study. ¶
• 8 additional weeks without stimulation, ending with one follow-up assessment visit ¶
¶
Outline the schedule and timing of study procedures. ¶
A. Consent and Screening Visit (Week -1) ¶
On their first visit, the research team will review the consent form with participants, confirm eligibility for the study, and may conduct basic assessments related to walking or mobility (completed in the participant's usual manner, including the use of a mobility device or wheelchair, if applicable). ¶
If participants prefer, the consent process may be completed remotely by emailing the IRB-approved consent form in advance. In such cases, participants will be given adequate time to review the document, and any questions will be addressed by the Principal Investigator or study personnel via phone, email, or Zoom, according to the participant's preference. ¶
¶
B. Stimulation Period and assessments (Weeks 1-8) ¶
Before the study commences, clinicians and the study team will perform baseline assessments that may take up to 4 hours (only 4 sessions with the assessment throughout the study; Week 1, 4, 8, and 16), and may include: ¶
1. Walking or mobility tests (completed in participants' usual way, including with a mobility device or wheelchair if applicable) ¶
2. Muscle strength testing ¶
3. Spasticity evaluations ¶
4. Symptom questionnaires ¶
5. Collect the first blood sample at baseline assessment ¶
¶
Participants will attend two visits per week for 16 total stimulation sessions. Each session lasts about 1.5 hours. During each session: ¶
a. Electrodes will be placed on the skin over the lower back. ¶
?Electrode placement may differ slightly depending on each individual and the tSCS device that is used. ¶
b. Participants will receive transcutaneous spinal cord stimulation (tSCS) at a comfortable, non-painful level. Participants may feel tingling or tapping sensations under the electrodes. ¶
?c. Participants may be asked to perform simple movements, seated or standing, depending on their mobility abilities. ¶
?d. Provide blood samples on first visit for stimulation during week 1, mid-study at week 4, and last visit for stimulation at week 8. ¶
?e. At Week 4 and at the end of the 8-week stimulation period, participants will: repeat walking or mobility tests, strength and spasticity assessments, and complete questionnaires as described above during week 1. ¶
¶
C. Follow-Up Assessment Visit (Week 16) ¶
About 8 weeks after the last stimulation session, participants will return for a final follow-up visit. Participants will repeat the same assessments, walking or mobility tests, strength testing, spasticity measures, questionnaires, and provide the fourth (last) blood draw. This helps researchers learn whether any changes continued after stimulation ended. ¶
¶
Procedures to be carried out with each participant ¶
¶
1. Transcutaneous Spinal Cord Stimulation (tSCS) ¶
Transcutaneous Spinal Cord Stimulation (tSCS) will be delivered using a non-invasive spinal neuromodulation system (TESCoN or SCONE, SpineX Inc., Northridge, California, USA/ ARCEX (Onward Medical, USA). Two self-adhesive round electrodes placed at the midline over the lower thoracic (T11–T12) and/or upper lumbar vertebral levels (L1-L2) will serve as the cathodes, and two rectangular self-adhesive electrodes placed symmetrically over the iliac crests will serve as anodes. Stimulation will be delivered as high-frequency pulsed current using frequencies within a predefined range (typically 1–90 Hz; with a carrier frequency to make stimulation more comfortable), and session parameters will be adjusted per participant and per visit. Current amplitude will begin at ~10 mA and be increased in small steps to a comfortable, tolerable level below strong muscle contraction or until responses plateau. Skin at electrode sites will be inspected, cleaned, and prepared to reduce impedance before each session. We will use a conducting gel to protect the skin during stimulation. A skin-temperature probe will be positioned adjacent to the cathode to monitor local temperature and reduce risk of skin irritation or burning. All stimulation sessions will be conducted at the UK PM&R Research Lab except the 4 sessions coupled with mobility assessments that will be conducted in the Human Performance Laboratory at the UK Campus under supervision of trained study personnel, with real-time monitoring for discomfort, autonomic symptoms, or adverse events. Participants may complete seated, standing, or mobility tasks during stimulation depending on their functional level, including those who ambulate with or without assistive devices and those who use wheelchairs. ¶
¶
2. Motor and Mobility Assessments ¶
Assessments will be adapted to participants' mobility level. Ambulatory participants will complete standard walking tests; wheelchair users will complete equivalent seated mobility tasks. ¶
a) 10-Meter Walk Test (or equivalent wheelchair speed test): 10MWT assesses walking speed over a 10-meter walkway at comfortable and maximum safe pace. 53 Timing occurs between 2 and 8 meters to exclude acceleration/deceleration, with two trials averaged per speed. Assistive devices are allowed. The 10MWT is quick, reliable, and sensitive to gait changes in HSP. Although no formal MCID exists for HSP, small gait speed changes reflect meaningful motor function improvements. ¶
b) 6-Minute Walk Test (or equivalent wheelchair endurance test): evaluates walking endurance by measuring the total distance walked over six minutes.53 Patients are instructed to walk as far as possible at their own pace, with rests allowed if needed. The total distance reflects functional capacity and stamina, which are often affected in HSP due to progressive spasticity and weakness ¶
c) Spastic Paraplegia Rating Scale (SPRS) clinician rating: SPRS is a reliable clinician-administered 13-item scale specific to HSP assessing gait, spasticity, strength, and sphincter function. Scores reflect disease severity and progression.54 ¶
d) HSP-SNAP patient-reported symptom questionnaire: HSP-SNAP is a patient-reported questionnaire rating frequency and severity of HSP symptoms such as stiffness, weakness, imbalance, and pain.55 It captures subjective symptom burden and treatment impact. ¶
e) 3D gait or movement analysis and surface EMG (when applicable): 3D Gait Analysis will be performed using a 14-camera motion capture system, wireless EMG system and 3 ground force plates.56,57 We will track walking speed using timing gates. Wireless EMG sensors will be placed on the vastus lateralis, rectus femoris, and vastus medialis to assess muscle activation during the gait trials. The outcomes will include walking velocity, stance time, joint kinematics (hip, knee and ankle) and joint kinetics. ¶
¶
3. Strength and Spasticity Testing ¶
a) Muscle Strength: Bilateral isometric knee strength will be obtained with three, 5-sec maximal voluntary isometric contractions (MVIC) separated by one-minute rest.56 EMG is recorded concurrently for normalization with gait data. Knee pain during contractions is recorded on an 11-point visual analog scale. ¶
b) Modified Ashworth Scale (MAS): It's the standard clinical scale to rate muscle spasticity (tightness) in HSP. It measures muscle spasticity by assessing resistance to passive muscle stretch through the range of motion and grades the muscle tone on a 0-4 scale based on the resistance felt.51 ¶
MAS 0-4 scale: ¶
0: No increased muscle tone ¶
1: Slight increase in tone; catch and release at end of range ¶
1+: Slight increase; catch followed by minimal resistance through < half the range ¶
2: More marked increase through most of the range, but limb still moves easily ¶
3: Considerable increase in tone; passive movement is difficult ¶
4: Limb is rigid in flexion or extension ¶
¶
4. Lower Urinary Tract (LUT) and Bowel Function: will be quantified as an exploratory outcome using NBSS and NBDS questionnaires. NBSS assesses urinary frequency, urgency, incontinence, and catheter use,58 while NBDS examines bowel movement frequency, constipation, and incontinence.59 ¶
¶
o Lower Urinary Tract (LUT) Assessments ¶
a) Neurogenic Bladder Symptom Score (NBSS): comprises 23 questions covering 3 domains, including incontinence, storage & voiding, and specific consequences, as well as one question on QoL. ¶
b) The Incontinence-QoL (I-QoL): comprises 10 questions covering 3 domains, including avoidance and limiting behaviour, psychosocial impacts, and social embarrassment, which will be summarised as a total score. All scores, for each domain and a total, will be transformed into a continuous scale value. ¶
o Bowel Dysfunction Assessments ¶
¶
a) Neurogenic bowel dysfunction score (NBD Score) The NBD score is a measure of both constipation and fecal incontinence and was developed for and validated in the SCI population This questionnaire comprises 10 questions focusing on defecation (i.e., frequency, duration, and clinical symptoms), constipation (i.e., use of aiding medication and digital stimulation), fecal incontinence (i.e., frequency, aiding medication, and flatus; and peri-anal skin problems. The consequential NBD score relates to four different neurogenic bowel dysfunction severity levels. ¶
5. Blood Collection and Metabolic/protein profiling ¶
Approximately 10 mL of venous blood samples will be collected at Weeks 1, 4, 8, and 16. Samples will be processed for metabolomic/lipid profiling and biomarker assays (including Big-tau). Targeted Metabolomic Profiling: Individuals with HSP also suffer with significant metabolic dysfunctions, notably in lipid metabolism.60 Many HSP genes encode proteins essential for lipid metabolism, with disturbances leading to impaired myelination, altered membrane trafficking, and axonal degeneration. Fasting plasma samples collected during the visits 2, 9, 17 and 18 will be analyzed for ~1100 lipid and polar metabolites across pathways including glycolysis, the tricarboxylic acid cycle, amino acid metabolism, and nucleotide biosynthesis [at our CNS Metabolism Center of Biomedical Research Excellence (COBRE) Metabolomics Core]. One plasma aliquot will be shipped to Drexel University for identification of Big-tau as a HSP biomarker (Dr. Emanuela Piermarini). Some analyses may involve genetic-related testing on de-identified samples. Individual research results will not be returned unless an important incidental finding is confirmed and IRB-approved. ¶
6. Statistical Analysis Plan ¶
Descriptive statistics will summarize feasibility (recruitment rate, retention, session adherence) and safety (adverse events). Efficacy outcomes will be analyzed using within-subject comparisons across timepoints (Week 1 vs Week 4 vs Week 8 vs Week 16). Depending on distribution, repeated-measures ANOVA or non-parametric Friedman tests will be used. Effect sizes (Cohen's d or Kendall's W) with 95% CIs will be reported to inform future powered trials. Missing data will be handled using mixed models or last-observation-carried-forward for sensitivity analyses. For Aim 1a, immediate effects of a single tSCS session will be assessed using paired t-tests. For Aim 1b, longitudinal changes across baseline, 8 weeks, and 16 weeks will be evaluated using linear mixed effects models to account for within-subject correlations and missing data. Primary and secondary outcomes (e.g., 10MWT, SPRS, MAS) will be treated as continuous variables. MAS, while ordinal, will be analyzed as continuous in the primary model with sensitivity analyses using nonparametric or ordinal methods. Post hoc comparisons will adjust for multiple testing. For Aim 2, which is exploratory and hypothesis-generating, univariate logistic regression will identify candidate predictors of motor response (defined as =0.1 m/s improvement in 10MWT or =1-point reduction in MAS). Candidate baseline predictors will include demographic and clinical variables such as age, sex, disease duration, genetic subtype, clinical phenotype (pure vs. complex), baseline gait speed, baseline MAS, lower-limb strength, and mobility status/assistive device use. Predictor-response associations will first be screened with univariate logistic regression. A multivariable logistic regression model will then be built using either backward stepwise selection guided by Akaike Information Criterion (AIC) or LASSO regularization, depending on sample size and collinearity. Model performance will be summarized by discrimination (AUROC) and calibration (Hosmer–Lemeshow test and calibration plots). Internal validation will be performed using 5-fold cross-validation and bootstrap resampling to assess stability and optimism. Finally, coefficients from the final model will be translated into a preliminary clinical scoring tool to stratify likelihood of response, acknowledging that external validation in a larger cohort will be required. Analyses will be conducted with a = 0.05, emphasizing estimation over significance testing. ¶
¶
This study does not involve deception. Participants will be fully informed about the purpose, procedures, risks, and voluntary nature of the study during the consent process. Therefore, no deception-related debriefing is required. ¶
¶
Standard/Routine Clinical Care (not part of the study) ¶
Participants will continue their usual clinical care for HSP throughout participation. Routine care may include, but is not limited to: ¶
• ongoing neurology/rehabilitation follow-up visits ¶
• physical therapy or occupational therapy prescribed as part of clinical care ¶
• standard medications for spasticity or symptoms (see medication rules below) ¶
• use of assistive devices such as braces, canes, walkers, or wheelchairs ¶
• other clinically indicated evaluations or treatments determined by their treating physicians ¶
These routine care procedures are not required by the research study and will occur whether or not the individual participates. ¶
¶
Research-Specific Procedures (performed only for this project) ¶
All of the following are conducted specifically for research and are not standard care for HSP: ¶
1. tSCS sessions (16 sessions over 8 weeks). ¶
2. Research assessments in Weeks 1, 4, 8, and 16, including: ¶
• walking tests or equivalent wheelchair mobility tests ¶
• endurance testing (6-minute walk or equivalent) ¶
• lower-limb strength testing ¶
• spasticity evaluation (Modified Ashworth Scale) ¶
• clinician rating (Spastic Paraplegia Rating Scale; SPRS) ¶
• participant symptom questionnaires (HSP-SNAP) ¶
• questionnaires to assess bladder and bowel function ¶
• optional 3D gait/movement and surface EMG testing, when applicable ¶
3. Research blood draws (4 total) at Weeks 1, 4, 8, and 16 for metabolomics/biomarkers/genetic-related analyses on de-identified samples. ¶
4. Additional monitoring for adverse events related to stimulation and study testing. ¶
¶
Medication Rules During Participation ¶
Permitted Medications ¶
Participants may continue stable standard-of-care medications, including: ¶
• antispasticity agents such as baclofen, tizanidine, dantrolene, diazepam/clonazepam ¶
• botulinum toxin injections, if already part of routine care ¶
• medications for bladder symptoms, pain, sleep, mood, or other chronic conditions ¶
• vitamins/supplements, if stable ¶
Requirement: All neurologic/spasticity-related medications must be stable for at least 4 weeks before enrollment and participants are asked to avoid dose changes during the 8-week stimulation period, unless medically necessary. ¶

¶
Forbidden / Restricted Medications or Interventions ¶
Participants will be excluded or paused if they initiate or substantially change treatments that could confound outcomes or increase risk, including: ¶
• starting a new antispasticity medication or making major dose changes during the 8-week stimulation phase (unless required for safety/medical care) ¶
• starting or changing neuromodulation therapies, including any implanted or external electrical stimulation targeting the spine or lower limbs ¶
• enrolling in another interventional clinical trial that could affect mobility/spasticity during participation ¶
• new implantation of electrical medical devices (e.g., pacemakers, intrathecal pumps) during the study ¶
If a medically necessary medication change occurs, the research team will document it and determine whether the participant can safely continue, pause, or withdraw, depending on timing and magnitude of the change. ¶
¶
Visit-by-visit listing all procedures that will take place: See attached file
Research Description ResearchProcedures changed by vkpa225 on 1/16/2026 12:03:06 PM
Participants will take part in a structured, supervised research program at Cardinal Hill Rehabilitation Hospital (CHRH) – Physical Medicine & Rehabilitation (PM&R) Research Lab (The PM&R department and laboratory space is leased by the University of Kentucky and located within CHRH; however, CHRH itself will not be involved in the conduct of this study in any other capacity) and the Human Performance Laboratory/Biodynamics Lab at the UK Campus. After a baseline assessment at the Human Performance Laboratory at the UK Campus, participants will attend twice-weekly sessions of noninvasive transcutaneous spinal cord stimulation (tSCS) for 8 weeks at ~~CHRH – Physical Medicine & Rehabilitation Research~~the PM&R Lab. Assessments at Week 1, 4, 8, and 16 will be conducted at the Human Performance Laboratory/Biodynamics lab at the UK Campus. Each stimulation visit will be monitored by trained research and clinical staff to ensure comfort and safety. Participants will receive stimulation while seated or (if able) during light mobility tasks. The study concludes with an end-of-treatment assessment and a single follow-up assessment visit 8 weeks later. ¶
¶
Participants will ~~take part~~participate in a structured, supervised research program at ~~Cardinal Hill Rehabilitation Hospital (CHRH) – Physical Medicine & Rehabilitation Research~~the CPM&R Lab and the Human Performance Laboratory ~~at~~/Biodynamics Lab on the UK Campus. After a baseline assessment at the Human Performance Laboratory at the UK Campus, participants will attend twice-weekly sessions of noninvasive transcutaneous spinal cord stimulation (tSCS) for 8 weeks at ~~CHRH – Physical Medicine & Rehabilitation Research Lab~~the PM&R Research Lab located in CHRH. Assessments at Week 1, 4, 8 and 16 will be conducted at the Human Performance Laboratory/Biodynamics Lab at the UK Campus. Each stimulation visit will be monitored by trained research and clinical staff to ensure comfort and safety. Participants will receive stimulation while seated or (if able) during light mobility tasks. The study concludes with an end-of-treatment assessment and a single follow-up assessment visit 8 weeks later. ¶
¶
Participants will be expected to: ¶
• Attend all scheduled lab visits over the 16-week study period. ¶
• Complete baseline, post-treatment, and follow-up assessments of motor function, mobility, strength, and spasticity. ¶
• Participate in 16 stimulation sessions over 8 weeks (2 sessions per week). ¶
• Complete questionnaires about symptoms and daily function. ¶
• Provide blood samples at four timepoints (Week 1, 4, 8, and 16). ¶
• Inform the study team about any changes in health, medications, or adverse events during the study. ¶
Testing and stimulation procedures will be adapted to each participant's mobility level, including people who walk with or without assistive devices and people who use wheelchairs. ¶
¶
Each participant will be in the study for about 16 weeks total: ¶
• ~~8~~ The stimulation intervention will be administered over an 8-weeks (2--months) ~~of~~period. Each stimulation ~~intervention~~session will last up to 1 hour, for a total of approximately 16 hours of stimulation across the study. ¶
• 8 additional weeks without stimulation, ending with one follow-up assessment visit ¶
¶
Outline the schedule and timing of study procedures. ¶
A. ~~Screening and Basel~~Consent and Screenineg Visit (Week -1) ¶
On their first visit, the research team will review the consent form with ~~the~~ participants, ~~ask questions to confirm if they qua~~confirm eligibilifty for the study, and may ~~perform some~~conduct basic assessments related to walking or mobility ~~tests~~ (completed in the participants's usual ~~way~~manner, including ~~wi~~the use of a mobility device or wheelchair, if applicable). ¶
If participants prefer, the consent process may be completed remotely by emailing the IRB-approved consent form in advance. In such cases, participants will be given adequate time to review the document, and any questions will be addressed by the Principal Investigator or study personnel via phone, email, or Zoom, according to the participant's preference. ¶
¶
B. Stimulation Period and assessments (Weeks 1-8) ¶
Before the study commences, clinicians and the study team will perform baseline assessments that may take up to 4 hours (only 4 sessions with the assessment throughout the study; Week 1, 4, 8, and 16), and may include: ¶
~~?~~ 1. Walking or mobility tests (completed in participants' usual way, including with a mobility device or wheelchair if applicable) ¶
~~?~~ 2. Muscle strength testing ¶
~~?~~ 3. Spasticity evaluations ¶
~~?~~ 4. Symptom questionnaires ¶
~~?~~ 5. Collect the first blood sample at baseline assessment ¶
¶
Participants will attend two visits per week for 16 total stimulation sessions. Each session lasts about 1.5 hours. During each session: ¶
~~?~~ a. Electrodes will be placed on the skin over the lower back. ¶
? Participants will receive transcutaneous spinal cord stimulation (tSCS) at a comfortable, non-painful level. Participants may feel tingling or tapping sensations under the electrodes. ¶
? Participants may be asked to perform simple movements, seated or standing, depending on their mobility abilities. ¶
? Provide blood samples on first visit for stimulation during week 1, mid-study at week 4, and last visit for stimulation at week 8. ¶
? At Week 4 and at the end of the 8-week stimulation period, participants will: repeat walking or mobility tests, strength and spasticity assessments, and complete questionnaires as described above during week 1. ¶
C. Follow-Up Assessment Visit (Week 16) ¶
About 8 weeks after last stimulation session, participants will return for a final follow-up visit. Participants will repeat the same assessments, walking or mobility tests, strength testing, spasticity measures, questionnaires and provide the fourth blood draw. This helps researchers learn whether any changes continued after stimulation ended. ¶
¶
Procedures to be carried out with each participant ¶
¶
1. Transcutaneous Spinal Cord Stimulation (tSCS) ¶
Transcutaneous Spinal Cord Stimulation (tSCS) will be delivered using a non-invasive spinal neuromodulation system (TESCoN or SCONE, SpineX Inc., Northridge, California, USA/ ARCEX (Onward Medical, USA). Two self-adhesive round electrodes placed at the midline over the lower thoracic (T11–T12) and/or upper lumbar vertebral levels (L1-L2) will serve as the cathodes, and two rectangular self-adhesive electrodes placed symmetrically over the iliac crests will serve as anodes. Stimulation will be delivered as high-frequency pulsed current using frequencies within a predefined range (typically 1–90 Hz; with a carrier frequency to make stimulation more comfortable), and session parameters will be adjusted per participant and per visit. Current amplitude will begin at ~10 mA and be increased in small steps to a comfortable, tolerable level below strong muscle contraction or until responses plateau. Skin at electrode sites will be inspected, cleaned, and prepared to reduce impedance before each session. We will use a conducting gel to protect the skin during stimulation. A skin-temperature probe will be positioned adjacent to the cathode to monitor local temperature and reduce risk of skin irritation or burning. All stimulation sessions will be conducted at the UK PM&R Research Lab except the 4 sessions coupled with mobility assessments that will be conducted in the Human Performance Laboratory at the UK Campus under supervision of trained study personnel, with real-time monitoring for discomfort, autonomic symptoms, or adverse events. Participants may complete seated, standing, or mobility tasks during stimulation depending on their functional level, including those who ambulate with or without assistive devices and those who use wheelchairs. ¶
¶
2. Motor and Mobility Assessments ¶
Assessments will be adapted to participants' mobility level. Ambulatory participants will complete standard walking tests; wheelchair users will complete equivalent seated mobility tasks. ¶
a) 10-Meter Walk Test (or equivalent wheelchair speed test): 10MWT assesses walking speed over a 10-meter walkway at comfortable and maximum safe pace. 53 Timing occurs between 2 and 8 meters to exclude acceleration/deceleration, with two trials averaged per speed. Assistive devices are allowed. The 10MWT is quick, reliable, and sensitive to gait changes in HSP. Although no formal MCID exists for HSP, small gait speed changes reflect meaningful motor function improvements. ¶
b) 6-Minute Walk Test (or equivalent wheelchair endurance test): evaluates walking endurance by measuring the total distance walked over six minutes.53 Patients are instructed to walk as far as possible at their own pace, with rests allowed if needed. The total distance reflects functional capacity and stamina, which are often affected in HSP due to progressive spasticity and weakness ¶
c) Spastic Paraplegia Rating Scale (SPRS) clinician rating: SPRS is a reliable clinician-administered 13-item scale specific to HSP assessing gait, spasticity, strength, and sphincter function. Scores reflect disease severity and progression.54 ¶
d) HSP-SNAP patient-reported symptom questionnaire: HSP-SNAP is a patient-reported questionnaire rating frequency and severity of HSP symptoms such as stiffness, weakness, imbalance, and pain.55 It captures subjective symptom burden and treatment impact. ¶
e) 3D gait or movement analysis and surface EMG (when applicable): 3D Gait Analysis will be performed using a 14-camera motion capture system, wireless EMG system and 3 ground force plates.56,57 We will track walking speed using timing gates. Wireless EMG sensors will be placed on the vastus lateralis, rectus femoris, and vastus medialis to assess muscle activation during the gait trials. The outcomes will include walking velocity, stance time, joint kinematics (hip, knee and ankle) and joint kinetics. ¶
¶
3. Strength and Spasticity Testing ¶
a) Muscle Strength: Bilateral isometric knee strength will be obtained with three, 5-sec maximal voluntary isometric contractions (MVIC) separated by one-minute rest.56 EMG is recorded concurrently for normalization with gait data. Knee pain during contractions is recorded on an 11-point visual analog scale. ¶
b) Modified Ashworth Scale (MAS): It's the standard clinical scale to rate muscle spasticity (tightness) in HSP. It measures muscle spasticity by assessing resistance to passive muscle stretch through the range of motion and grades the muscle tone on a 0-4 scale based on the resistance felt.51 ¶
MAS 0-4 scale: ¶
0: No increased muscle tone ¶
1: Slight increase in tone; catch and release at end of range ¶
1+: Slight increase; catch followed by minimal resistance through < half the range ¶
2: More marked increase through most of the range, but limb still moves easily ¶
3: Considerable increase in tone; passive movement is difficult ¶
4: Limb is rigid in flexion or extension ¶
¶
4. Lower Urinary Tract (LUT) and Bowel Function: will be quantified as an exploratory outcome using NBSS and NBDS questionnaires. NBSS assesses urinary frequency, urgency, incontinence, and catheter use,58 while NBDS examines bowel movement frequency, constipation, and incontinence.59 ¶
¶
o Lower Urinary Tract (LUT) Assessments ¶
a) Neurogenic Bladder Symptom Score (NBSS): comprises 23 questions covering 3 domains, including incontinence, storage & voiding, and specific consequences, as well as one question on QoL. ¶
b) The Incontinence-QoL (I-QoL): comprises 10 questions covering 3 domains, including avoidance and limiting behaviour, psychosocial impacts, and social embarrassment, which will be summarised as a total score. All scores, for each domain and a total, will be transformed into a continuous scale value. ¶
o Bowel Dysfunction Assessments ¶
¶
a) Neurogenic bowel dysfunction score (NBD Score) The NBD score is a measure of both constipation and fecal incontinence and was developed for and validated in the SCI population This questionnaire comprises 10 questions focusing on defecation (i.e., frequency, duration, and clinical symptoms), constipation (i.e., use of aiding medication and digital stimulation), fecal incontinence (i.e., frequency, aiding medication, and flatus; and peri-anal skin problems. The consequential NBD score relates to four different neurogenic bowel dysfunction severity levels. ¶
5. Blood Collection and Metabolic/protein profiling ¶
Approximately 10 mL of venous blood samples will be collected at Weeks 1, 4, 8, and 16. Samples will be processed for metabolomic/lipid profiling and biomarker assays (including Big-tau). Targeted Metabolomic Profiling: Individuals with HSP also suffer with significant metabolic dysfunctions, notably in lipid metabolism.60 Many HSP genes encode proteins essential for lipid metabolism, with disturbances leading to impaired myelination, altered membrane trafficking, and axonal degeneration. Fasting plasma samples collected during the visits 2, 9, 17 and 18 will be analyzed for ~1100 lipid and polar metabolites across pathways including glycolysis, the tricarboxylic acid cycle, amino acid metabolism, and nucleotide biosynthesis [at our CNS Metabolism Center of Biomedical Research Excellence (COBRE) Metabolomics Core]. One plasma aliquot will be shipped to Drexel University for identification of Big-tau as a HSP biomarker (Dr. Emanuela Piermarini). Some analyses may involve genetic-related testing on de-identified samples. Individual research results will not be returned unless an important incidental finding is confirmed and IRB-approved. ¶
6. Statistical Analysis Plan ¶
Descriptive statistics will summarize feasibility (recruitment rate, retention, session adherence) and safety (adverse events). Efficacy outcomes will be analyzed using within-subject comparisons across timepoints (Week 1 vs Week 4 vs Week 8 vs Week 16). Depending on distribution, repeated-measures ANOVA or non-parametric Friedman tests will be used. Effect sizes (Cohen's d or Kendall's W) with 95% CIs will be reported to inform future powered trials. Missing data will be handled using mixed models or last-observation-carried-forward for sensitivity analyses. For Aim 1a, immediate effects of a single tSCS session will be assessed using paired t-tests. For Aim 1b, longitudinal changes across baseline, 8 weeks, and 16 weeks will be evaluated using linear mixed effects models to account for within-subject correlations and missing data. Primary and secondary outcomes (e.g., 10MWT, SPRS, MAS) will be treated as continuous variables. MAS, while ordinal, will be analyzed as continuous in the primary model with sensitivity analyses using nonparametric or ordinal methods. Post hoc comparisons will adjust for multiple testing. For Aim 2, which is exploratory and hypothesis-generating, univariate logistic regression will identify candidate predictors of motor response (defined as =0.1 m/s improvement in 10MWT or =1-point reduction in MAS). Candidate baseline predictors will include demographic and clinical variables such as age, sex, disease duration, genetic subtype, clinical phenotype (pure vs. complex), baseline gait speed, baseline MAS, lower-limb strength, and mobility status/assistive device use. Predictor-response associations will first be screened with univariate logistic regression. A multivariable logistic regression model will then be built using either backward stepwise selection guided by Akaike Information Criterion (AIC) or LASSO regularization, depending on sample size and collinearity. Model performance will be summarized by discrimination (AUROC) and calibration (Hosmer–Lemeshow test and calibration plots). Internal validation will be performed using 5-fold cross-validation and bootstrap resampling to assess stability and optimism. Finally, coefficients from the final model will be translated into a preliminary clinical scoring tool to stratify likelihood of response, acknowledging that external validation in a larger cohort will be required. Analyses will be conducted with a = 0.05, emphasizing estimation over significance testing. ¶
¶
This study does not involve deception. Participants will be fully informed about the purpose, procedures, risks, and voluntary nature of the study during the consent process. Therefore, no deception-related debriefing is required. ¶
¶
Standard/Routine Clinical Care (not part of the study) ¶
Participants will continue their usual clinical care for HSP throughout participation. Routine care may include, but is not limited to: ¶
• ongoing neurology/rehabilitation follow-up visits ¶
• physical therapy or occupational therapy prescribed as part of clinical care ¶
• standard medications for spasticity or symptoms (see medication rules below) ¶
• use of assistive devices such as braces, canes, walkers, or wheelchairs ¶
• other clinically indicated evaluations or treatments determined by their treating physicians ¶
These routine care procedures are not required by the research study and will occur whether or not the individual participates. ¶
¶
Research-Specific Procedures (performed only for this project) ¶
All of the following are conducted specifically for research and are not standard care for HSP: ¶
1. tSCS sessions (16 sessions over 8 weeks). ¶
2. Research assessments in Weeks 1, 4, 8, and 16, including: ¶
• walking tests or equivalent wheelchair mobility tests ¶
• endurance testing (6-minute walk or equivalent) ¶
• lower-limb strength testing ¶
• spasticity evaluation (Modified Ashworth Scale) ¶
• clinician rating (Spastic Paraplegia Rating Scale; SPRS) ¶
• participant symptom questionnaires (HSP-SNAP) ¶
• questionnaires to assess bladder and bowel function ¶
• optional 3D gait/movement and surface EMG testing, when applicable ¶
3. Research blood draws (4 total) at Weeks 1, 4, 8, and 16 for metabolomics/biomarkers/genetic-related analyses on de-identified samples. ¶
4. Additional monitoring for adverse events related to stimulation and study testing. ¶
¶
Medication Rules During Participation ¶
Permitted Medications ¶
Participants may continue stable standard-of-care medications, including: ¶
• antispasticity agents such as baclofen, tizanidine, dantrolene, diazepam/clonazepam ¶
• botulinum toxin injections, if already part of routine care ¶
• medications for bladder symptoms, pain, sleep, mood, or other chronic conditions ¶
• vitamins/supplements, if stable ¶
Requirement: All neurologic/spasticity-related medications must be stable for at least 4 weeks before enrollment and participants are asked to avoid dose changes during the 8-week stimulation period, unless medically necessary. ¶
¶
Forbidden / Restricted Medications or Interventions ¶
Participants will be excluded or paused if they initiate or substantially change treatments that could confound outcomes or increase risk, including: ¶
• starting a new antispasticity medication or making major dose changes during the 8-week stimulation phase (unless required for safety/medical care) ¶
• starting or changing neuromodulation therapies, including any implanted or external electrical stimulation targeting the spine or lower limbs ¶
• enrolling in another interventional clinical trial that could affect mobility/spasticity during participation ¶
• new implantation of electrical medical devices (e.g., pacemakers, intrathecal pumps) during the study ¶
If a medically necessary medication change occurs, the research team will document it and determine whether the participant can safely continue, pause, or withdraw, depending on timing and magnitude of the change. ¶
¶
Visit-by-visit listing all procedures that will take place: See attached file
Research Description Resources changed by vkpa225 on 1/16/2026 2:46:20 PM
The study team includes investigators and staff with complementary clinical, neuromodulation, biomechanics, metabolomics, and data-science expertise, all of whom are available within UK-PM&R research lab and the Human Performance Laboratory/Biodynamics lab at the UK Campus to support recruitment, intervention delivery, assessments, and analysis. The BioDynamics laboratories at the University of Kentucky will be utilized for the gait analyses and strength testing performed in this study. ¶
¶

Staffing and Personnel: ¶
Dr. Rahul Sachdeva (PI) – Assistant Professor, PM&R. Brings ~15 years of experience in neuromodulation and tSCS across neurological populations and will oversee all study activities and safety. ¶
Dr. Michael Abeeb Samaan (Co-Investigator) – Associate Professor and Co-Director of the Human Biodynamics Lab. Provides expertise in 3D gait analysis, EMG, and strength assessment. ¶
Dr. Sara Salles (Co-Investigator) – Professor/Clinician, PM&R. Supports recruitment from the established HSP clinical cohort and performs clinical scales (SPRS, MAS). ¶
Dr. Samirkumar Patel (Co-Investigator) – Assistant Professor and Co-Director of the CNS Metabolism COBRE Metabolomics Core. Leads metabolomics/biomarker workflows. ¶
Dr. Vivek Pandey (Co-Investigator) – Scientist II with ~11 years of experience in molecular biology, neurodegeneration, and data handling, and certified phlebotomist; will manage daily operations, including recruitment, tSCS delivery, biospecimen handling, and data analysis. ¶
Ms. Makyra Ross (Study personnel) – Graduate student with research experience in hereditary spastic paraplegia (HSP) studies conducted in Dr. Sachdeva's laboratory. Assists with daily operations including recruitment, tSCS delivery, biospecimen handling, and data analysis. ¶
Dr. Zakari Dymock (Study personnel) – SCI Physician and Assistant Professor, PM&R. Contributes to recruitment and administers validated clinical scales, including the SPRS and MAS. ¶
Dr. Susan McDowell (Study personnel) – Janet Galloway Carter Endowed Chairperson, Associate Professor, Physical Medicine and Rehabilitation, oversees recruitment of participants, clinical procedures and data analysis. ¶
Ms. Hannah Whitaker (Study personnel) – Clinical Coordinator and certified phlebotomist, will assist with biomechanical data collection and blood sampling/processing. ¶
Ms. Holly Elliott (Study personnel) – Research Engineer, will assist with data collection and processing of motion capture and muscle function testing data. ¶
Biostatistics support – A designated biostatistical consultant (Dr. Rich Cohn) ~~and an establis~~will support the~~d~~ analysis ~~plan ensure adequate expertise for data monitoring and analysis~~of anonymized data. ¶
¶
Primary site: Dr. Sachdeva's clinical research lab within the PM&R Department at Cardinal Hill Rehabilitation Hospital (CHRH) and Dr. Samaan's Human Performance Laboratory/Biodynamics Lab at the UK campus. ¶
Space and access: The lab is a 1,400-sq-ft dedicated clinical research space, located near the main entrance for easy access for participants using assistive devices or wheelchairs. ¶
Capabilities: The lab is equipped for comprehensive motor, autonomic, and metabolic assessments in individuals with HSP. ¶
• Computer or other technological resources, mobile or otherwise, required or created during the conduct of the research; ¶
• tSCS equipment: Noninvasive neuromodulation delivered with the SCONE system (SpineX Inc.)/ARCEX (Onward Medical), with device support and troubleshooting available through the manufacturer. ¶
• Biomechanics/EMG: Dr. Samaan's Lab provides advanced 3D motion capture, wireless EMG, and force-plate systems to support all proposed biomechanical outcomes. ¶
• Data systems: Research data will be captured on secure, password-protected UK servers/databases with coded identifiers and role-based access, consistent with institutional policy and HIPAA protections (details in confidentiality section). ¶
• Clinical oversight: Stimulation and assessment visits occur under the supervision of principal investigator, Dr. Rahul Sachdeva, trained research personnel, and medical supervisor, with safety screening at each visit. ¶
• Skin and stimulation safety: The team has established procedures such as using gel for safe parameter titration, temperature monitoring, and real-time intervention if discomfort or skin heating occurs. ¶
• Participant contact: The team has established phone/email/secure messaging systems for scheduling, reminders, and follow-ups. ¶
• Accessibility: CHRH/UK facilities are wheelchair-accessible and appropriate for participants with varying mobility limitations. ¶
• Language services (as needed): UK HealthCare interpretation/translation services are available for participants who require communication support in languages other than English (standard institutional resource).
Research Description Resources changed by vkpa225 on 1/16/2026 12:20:02 PM
The study team includes investigators and staff with complementary clinical, neuromodulation, biomechanics, metabolomics, and data-science expertise, all of whom are available within UK/~~CHRH~~-PM&R research lab and the Human Performance Laboratory/Biodynamics lab at the UK Campus to support recruitment, intervention delivery, assessments, and analysis. The BioDynamics ~~and BioMotion~~ laboratories at the University of Kentucky will be utilized for the gait analyses and strength testing performed in this study. ¶
¶
Staffing and Personnel: ¶
Dr. Rahul Sachdeva (PI) – Assistant Professor, PM&R. Brings ~15 years of experience in neuromodulation and tSCS across neurological populations and will oversee all study activities and safety. ¶
Dr. Michael Abeeb Samaan (Co-Investigator) – Associate Professor and Co-Director of the Human Biodynamics Lab. Provides expertise in 3D gait analysis, EMG, and strength assessment. ¶
Dr. Sara Salles (Co-Investigator) – Professor/Clinician, PM&R. Supports recruitment from the established HSP clinical cohort and performs clinical scales (SPRS, MAS). ¶
Dr. Samirkumar Patel (Co-Investigator) – Assistant Professor and Co-Director of the CNS Metabolism COBRE Metabolomics Core. Leads metabolomics/biomarker workflows. ¶
Dr. Vivek Pandey (Co-Investigator) – Scientist II with ~11 years of experience in molecular biology, neurodegeneration, and data handling, and certified phlebotomist; will manage daily operations, including recruitment, tSCS delivery, biospecimen handling, and data analysis. ¶
Ms. Makyra Ross (Study personnel) – Graduate student with research experience in hereditary spastic paraplegia (HSP) studies conducted in Dr. Sachdeva's laboratory. Assists with daily operations including recruitment, tSCS delivery, biospecimen handling, and data analysis. ¶
Dr. Zakari Dymock (Study personnel) – SCI Physician and Assistant Professor, PM&R. Contributes to recruitment and administers validated clinical scales, including the SPRS and MAS. ¶
Dr. Susan McDowell (Study personnel) – Janet Galloway Carter Endowed Chairperson, Associate Professor, Physical Medicine and Rehabilitation, oversees recruitment of participants, clinical procedures and data analysis. ¶
~~Additional trained lab staff (TBD) – Two trained research staff will assist with biomechanical data collection and blood sampling/processing~~Ms. Hannah Whitaker (Study personnel) – Clinical Coordinator and certified phlebotomist, will assist with biomechanical data collection and blood sampling/processing. ¶
Ms. Holly Elliott (Study personnel) – Research Engineer, will assist with data collection and processing of motion capture and muscle function testing data. ¶
Biostatistics support – A designated biostatistical consultant (Dr. Rich Cohn) and an established analysis plan ensure adequate expertise for data monitoring and analysis. ¶
¶
Primary site: Dr. Sachdeva's clinical research lab within the PM&R Department at Cardinal Hill Rehabilitation Hospital (CHRH) and Dr. Samaan's Human Performance Laboratory/Biodynamics Lab at the UK campus. ¶
Space and access: The lab is a 1,400-sq-ft dedicated clinical research space, located near the main entrance for easy access for participants using assistive devices or wheelchairs. ¶
Capabilities: The lab is equipped for comprehensive motor, autonomic, and metabolic assessments in individuals with HSP. ¶
• Computer or other technological resources, mobile or otherwise, required or created during the conduct of the research; ¶
• tSCS equipment: Noninvasive neuromodulation delivered with the SCONE system (SpineX Inc.)/ARCEX (Onward Medical), with device support and troubleshooting available through the manufacturer. ¶
• Biomechanics/EMG: Dr. Samaan's Lab provides advanced 3D motion capture, wireless EMG, and force-plate systems to support all proposed biomechanical outcomes. ¶
• Data systems: Research data will be captured on secure, password-protected UK servers/databases with coded identifiers and role-based access, consistent with institutional policy and HIPAA protections (details in confidentiality section). ¶
¶
• Clinical oversight: Stimulation and assessment visits occur under the supervision of principal investigator, Dr. Rahul Sachdeva, trained research personnel, and medical supervisor, with safety screening at each visit. ¶
• Skin and stimulation safety: The team has established procedures such as using gel for safe parameter titration, temperature monitoring, and real-time intervention if discomfort or skin heating occurs. ¶
• Participant contact: The team has established phone/email/secure messaging systems for scheduling, reminders, and follow-ups. ¶
• Accessibility: CHRH/UK facilities are wheelchair-accessible and appropriate for participants with varying mobility limitations. ¶
• Language services (as needed): UK HealthCare interpretation/translation services are available for participants who require communication support in languages other than English (standard institutional resource).
Research Description SubjectRecruitmentMethods changed by vkpa225 on 1/16/2026 11:53:03 AM
Potential participants will be identified using multiple complementary sources to ensure feasibility for this rare disease. These sources will include: ¶
1. Clinical records/databases within UK HealthCare/PM&R and Cardinal Hill Rehabilitation Hospital (CHRH), including an established HSP database of >40 individuals maintained through the CHRH catchment area and clinician collaborators. ¶
2. Clinician referral, including referrals from study co-investigators (e.g., Dr. Sara Salles) and other PM&R/Neurology providers who care for individuals with HSP. ¶
3. Community and foundation connections, including individuals who previously expressed interest following study team presentations to the Spastic Paraplegia Foundation (SPF) community (e.g., SPF Talks webinar). ¶
4. Online and community-based organizations, including HSP advocacy groups and registries, where permitted. ¶
¶
Importantly, recruitment will be inclusive of people with diverse mobility abilities, including those who walk with or without assistive devices and those who use wheelchairs for some or all mobility. ¶
¶
Yes. IRB-approved advertisements (flyers, email scripts, and brief messages) will be used and distributed through: ¶
1. CHRH/UK clinic postings in PM&R and affiliated neurology or rehabilitation spaces. ¶
2. Online channels such as lab/department websites and social media posts approved by IRB. ¶
3. Community-based organizations and registries, including SPF communication outlets and other HSP advocacy groups where allowed. ¶
4. Direct follow-up to prior interested individuals who contacted the lab after SPF Talks or related engagement. ¶
¶
For individuals identified or contacted through neutral or portal messaging, a single follow-up neutral message may be sent if there is no initial response. No further contact will be made if the individual does not respond. ¶
Potential participants will meet with the study team: ¶
¶
1. In person at the UK PM&R Research Lab located within CHRH for screening and informed consent, in a private setting. ¶
2. Remotely (phone/video) for pre-screening and initial questions when appropriate, prior to scheduling an in-person screening visit. ¶
Participants will be given adequate time to review consent documents, ask questions, and decide without pressure. ¶
¶
Because HSP is a rare condition, broad community outreach is essential. To promote equitable enrollment, the team will: ¶
1. Recruit through both clinical systems and community/foundation networks, which helps reach individuals who may not be seen frequently in tertiary centers. ¶
2. Use accessible, lay-language materials and inclusive imagery/language welcoming a wide range of functional abilities, including wheelchair users. ¶
3. Offer flexible scheduling and accessibility supports to reduce barriers for participants with transportation or mobility limitations. ¶
Enrollment will be monitored to ensure no group is unintentionally excluded. ¶
¶
To minimize undue influence: ¶
• All recruitment materials and conversations will emphasize that participation is voluntary and that choosing not to participate will not affect current or future care. ¶
• Initial contact with clinic-identified individuals will be made in a non-pressuring way (e.g., clinician introduction or neutral letter/portal message) and will invite the individual to contact the study team if interested. ¶
• No claims of guaranteed benefit will be made. Recruitment will describe potential benefits and risks in balanced terms. ¶
• compensation is offered and it will be presented as reimbursement for time/effort, not as a benefit, and will be prorated if a participant withdraws early.
Research Sites CardinalHillHospital changed by vkpa225 on 1/16/2026 2:48:28 PM
Y
Research Sites MultiSiteDesc changed by vkpa225 on 1/16/2026 1:00:49 PM
Activities conducted by non-University of Kentucky collaborators involve only the analysis of de-identified data or de-identified biospecimens and do not include any interaction with study participants or access to identifiable information. Dr. Rich Cohn will assist solely with statistical analysis of de-identified datasets, and Drexel University will receive only de-identified plasma samples for biomarker analysis. As such, the risk of unanticipated problems, noncompliance, or protocol deviations at non-UK sites is minimal. Any unexpected issues related to data integrity or protocol conduct that could impact the study will be promptly communicated to the University of Kentucky Principal Investigator and reported to the UK IRB in accordance with institutional requirements.
Research Sites Other changed by vkpa225 on 1/16/2026 12:59:34 PM
Plasma aliquot will be shipped to Drexel University for the identification of Big tau as a potential biomarker for hereditary spastic paraplegia (HSP) under the direction of Dr. Emanuela Piermarini. All samples will be de-identified before shipment. Dr. Piermarini and her research team will have no interaction with study participants and will not have access to identifiable participant information.
Research Sites Other2 changed by vkpa225 on 1/16/2026 12:59:34 PM
Dr. Rich Cohn, Biostatistician, will serve as an independent consultant and will assist with data analysis. He will have no interaction with study participants and will not have access to identifiable participant information.
Subject Demographics AppalachianPopulation changed by vkpa225 on 1/16/2026 10:19:45 AM
~~Y~~
Subject Demographics IfUnknownExplain changed by vkpa225 on 1/16/2026 10:19:45 AM
Demographic information will be updated at the continuation review
Subject Demographics StudyPopulation changed by vkpa225 on 1/16/2026 10:19:45 AM
This pilot study will target recruiting 15 individuals with genetically confirmed or clinically diagnosed HSP. ¶
Inclusion Criteria: ¶
• Age 18 years or above. ¶
• Clinical diagnosis of hereditary spastic paraplegia (genetic confirmation if available). ¶
• Stable medications for spasticity and other neurologic symptoms for =4 weeks prior to enrollment. ¶
• Able to participate in study visits and assessments with or without assistive devices. ¶
• If ambulatory: able to walk at least 10 meters with or without an assistive device. ¶
• If wheelchair user: able to perform seated mobility tasks and transfers required for assessments. ¶
• Capacity to provide informed consent and follow study procedures, with an ability to communicate and understand instructions in English. ¶
Exclusion Criteria: ¶
• Implanted electronic devices (e.g., pacemaker, deep brain stimulator, intrathecal pumps). ¶
• Severe cardiopulmonary disease that would make participation unsafe. ¶
• Open skin lesions or severe dermatologic conditions at electrode sites. ¶
• Pregnancy or plans to become pregnant during the intervention period. ¶
• Diagnosed with Primary Lateral Sclerosis (PLS) or another neurological condition that affects walking, such as stroke, multiple sclerosis (MS), or a recent surgery on legs. ¶
• Unable to participate in basic movement or mobility assessments, even with their usual mobility device (such as a wheelchair, walker, or cane). ¶
Note: People who use wheelchairs or other mobility aids can participate if they can complete the study's mobility assessments in their usual way. ¶
• Cognitive or psychiatric conditions that make it difficult to give informed consent or follow study instructions. ¶
• Diagnosed with Urinary Tract Infection (UTI), either acute or ongoing, before or at the time of study enrollment. ¶
• Diagnosed with epilepsy. ¶
• Participation in another interventional clinical trial that could affect mobility or spasticity during the study. ¶
• A recent change (within the last 4 weeks) in medications or treatments that affect spasticity or movement (for example: baclofen, tizanidine, botulinum toxin injections). ¶
• Expect to start or change treatments for spasticity or mobility during the study period. ¶
• Any condition judged by the investigator to pose excess risk or confound outcomes.

Study Personnel Changes:

Status	PPIdentity	ProtocolID	PersonID	RoleInProtocol	IsContact	LastName	FirstName	Email	DeptCode	RoomBuilding	SpeedSort	PhoneNum	DeptDesc	AuthorizedConsent	ResponsibilityInProject	Degree	Rank	StatusFlag	IsRemoved	ModBy	ModDate	SFI	IsPIRN	MiddleName
Inserted	1156589	109294	12714654	SP	N	Whitaker	Hannah	Hannah.Whitaker@uky.edu						N	Data Collection			P	N	vkpa225	1/16/2026 10:31:25 AM	N		
Inserted	1156590	109294	12362832	SP	N	Elliott	Holly	hstanze4240@uky.edu						N	Data Collection			P	N	vkpa225	1/16/2026 2:26:19 PM	N		

Protocol Type Comment by Samuel Bell - ORI to PI on 1/15/2026 2:29:01 PM
The IRB requested minor revisions. Please see the attached document labeled "IRB Requested Revisions" in the "additional information/materials" section of the protocol. This document is also accessible via "all attachments."

If you have any questions about the revisions, feel free to contact me at your convenience.

Sam Bell
samuel.bell@uky.edu
323-6744 OR message me on Teams

Informed Consent Comment by Samuel Bell - ORI to IRB/PI on 1/21/2026 3:18:03 PM
ORI changed tracked consent document type to "highlighted changes" so only the clean copy of the consent gets an IRB approval stamp.
